# Privacy-Aware Distributed Hypothesis Testing †

**DOI:** 10.3390/e22060665

**Published:** 2020-06-16

**Authors:** Sreejith Sreekumar, Asaf Cohen, Deniz Gündüz

**Affiliations:** 1 Department of Electrical and Computer Engineering , Cornell University, Ithaca, NY 14850, USA; 2 The School of Electrical and Computer Engineering, Ben-Gurion University of the Negev, Beer-Sheva 8410501, Israel; coasaf@bgu.ac.il; 3 Department of Electrical and Electronic Engineering, Imperial College London, London SW72AZ, UK; d.gunduz@imperial.ac.uk

**Keywords:** Hypothesis testing, privacy, testing against conditional independence, error exponent, equivocation, distortion, causal disclosure

## Abstract

A distributed binary hypothesis testing (HT) problem involving two parties, a remote observer and a detector, is studied. The remote observer has access to a discrete memoryless source, and communicates its observations to the detector via a rate-limited noiseless channel. The detector observes another discrete memoryless source, and performs a binary hypothesis test on the joint distribution of its own observations with those of the observer. While the goal of the observer is to maximize the type II error exponent of the test for a given type I error probability constraint, it also wants to keep a private part of its observations as oblivious to the detector as possible. Considering both equivocation and average distortion under a causal disclosure assumption as possible measures of privacy, the trade-off between the communication rate from the observer to the detector, the type II error exponent, and privacy is studied. For the general HT problem, we establish single-letter inner bounds on both the rate-error exponent-equivocation and rate-error exponent-distortion trade-offs. Subsequently, single-letter characterizations for both trade-offs are obtained (i) for testing against conditional independence of the observer’s observations from those of the detector, given some additional side information at the detector; and (ii) when the communication rate constraint over the channel is zero. Finally, we show by providing a counter-example where the strong converse which holds for distributed HT without a privacy constraint does not hold when a privacy constraint is imposed. This implies that in general, the rate-error exponent-equivocation and rate-error exponent-distortion trade-offs are not independent of the type I error probability constraint.

## 1. Introduction

Data inference and privacy are often contradicting objectives. In many multi-agent system, each agent/user reveals information about its data to a remote service, application or authority, which in turn, provides certain utility to the users based on their data. Many emerging networked systems can be thought of in this context, from social networks to smart grids and communication networks. While obtaining the promised utility is the main goal of the users, privacy of data that is shared is becoming increasingly important. Thus, it is critical that the users ensure a desired level of privacy for the sensitive information revealed, while maximizing the utility subject to this constraint.

In many distributed learning or distributed decision-making applications, typically the goal is to learn the joint probability distribution of data available at different locations. In some cases, there may be prior knowledge about the joint distribution, for example, that it belongs to a certain set of known probability distributions. In such a scenario, the nodes communicate their observations to the detector, which then applies hypothesis testing (HT) on the underlying joint distribution of the data based on its own observations and those received from other nodes. However, with the efficient data mining and machine learning algorithms available today, the detector can illegitimately infer some unintended private information from the data provided to it exclusively for HT purposes. Such threats are becoming increasingly imminent as large amounts of seemingly irrelevant yet sensitive data are collected from users, such as in medical research [1], social networks [2], online shopping [3] and smart grids [4]. Therefore, there is an inherent trade-off between the utility acquired by sharing data and the associated privacy leakage.

There are several practical scenarios where the above-mentioned trade-off arises. For example, consider the issue of consumer privacy in the context of online shopping. A consumer would like to share some information about his/her shopping behavior, e.g., shopping history and preferences, with the shopping portal to get better deals and recommendations on relevant products. The shopping portal would like to determine whether the consumer belongs to its target age group (e.g., below 30 years old) before sending special offers to this customer. Assuming that the shopping patterns of the users within and outside the target age groups are independent, the shopping portal performs a hypothesis test to check if the consumer’s shared data is correlated with the data of its own customers. If the consumer is indeed within the target age group, the shopping portal would like to gather more information about this potential customer, particular interests, more accurate age estimation, etc.; while the user is reluctant to provide any further information. Yet another relevant example is the issue of user privacy in the context of wearable Internet of Things (IoT) devices such as smart watches and fitness trackers, which collect information on routine daily activities, and often have a third-party cloud interface.

In this paper, we study distributed HT (DHT) with a privacy constraint, in which an *observer* communicates its observations to a *detector* over a noiseless rate-limited channel of rate *R* nats per observed sample. Using the data received from the observer, the detector performs binary HT on the joint distribution of its own observations and those of the observer. The performance of the HT is measured by the asymptotic exponential rate of decay of the type II error probability, known as the type II error exponent (or error exponent henceforth), for a given constraint on the type I error probability (definitions will be given below). While the goal is to maximize the performance of the HT, the observer also wants to maintain a certain level of privacy against the detector for some latent private data that is correlated with its observations. We are interested in characterizing the trade-off between the communication rate from the observer to the detector over the channel, error exponent achieved by the HT and the amount of information leakage of private data. A special case of HT known as *testing against conditional independence* (TACI) will be of particular interest. In TACI, the detector tests whether its own observations are independent of those at the observer, conditioned on additional side information available at the detector.

### 1.1. Background

Distributed HT without any privacy constraint has been studied extensively from an information- theoretic perspective in the past, although many open problems remain. The fundamental results for this problem are first established in [5], which includes a single-letter lower bound on the optimal error exponent and a *strong converse* result which states that the optimal error exponent is independent of the constraint on the type I error probability. Exact single-letter characterization of the optimal error exponent for the testing against independence (TAI) problem, i.e., TACI with no side information at the detector, is also obtained. The lower bound established in [5] is further improved in [6,7]. Strong converse is studied in the context of complete data compression and zero-rate compression in [6,8], respectively, where in the former, the observer communicates to the detector using a message set of size two, while in the latter using a message set whose size grows sub-exponentially with the number of observed samples. The TAI problem with multiple observers remains open (similar to several other distributed compression problems when a non-trivial fidelity criterion is involved); however, the optimal error exponent is obtained in [9] when the sources observed at different observers follow a certain Markov relation. The scenario in which, in addition to HT, the detector is also interested in obtaining a reconstruction of the observer’s source, is studied in [10]. The authors characterize the trade-off between the achievable error exponent and the average distortion between the observer’s observations and the detector’s reconstruction. The TACI is first studied in [11], where the optimality of a random binning-based encoding scheme is shown. The optimal error exponent for TACI over a noisy communication channel is established in [12]. Extension of this work to general HT over a noisy channel is considered in [13], where lower bounds on the optimal error exponent are obtained by using a separation-based scheme and also using hybrid coding for the communication between the observer and the detector. The TACI with a single observer and multiple detectors is studied in [14], where each detector tests for the conditional independence of its own observations from those of the observer. The general HT version of this problem over a noisy broadcast channel and DHT over a multiple access channel is explored in [15]. While all the above works consider the asymmetric objective of maximizing the error exponent under a constraint on the type I error probability, the trade-off between the exponential rate of decay of both the type I and type II error probabilities are considered in [16,17,18].

Data privacy has been a hot topic of research in the past decade, spanning across multiple disciplines in computer and computational sciences. Several practical schemes have been proposed that deal with the protection or violation of data privacy in different contexts, e.g., see [19,20,21,22,23,24]. More relevant for our work, HT under mutual information and maximal leakage privacy constraints have been studied in [25,26], respectively, where the observer uses a *memoryless privacy mechanism* to convey a noisy version of its observed data to the detector. The detector performs HT on the probability distribution of the observer’s data, and the optimal privacy mechanism that maximizes the error exponent while satisfying the privacy constraint is analyzed. Recently, a distributed version of this problem has been studied in [27], where the observer applies a privacy mechanism to its observed data prior to further coding for compression, and the goal at the detector is to perform a HT on the joint distribution of its own observations with those of the observer. In contrast with [25,26,27], we study *DHT with a privacy constraint*, but without considering a separate privacy mechanism at the observer. In Section 2, we will further discuss the differences between the system model considered here and that of [27].

It is important to note here that the data privacy problem is fundamentally different from that of data security against an eavesdropper or an adversary. In data security, sensitive data is to be protected against an external malicious agent distinct from the legitimate parties in the system. The techniques for guaranteeing data security usually involve either cryptographic methods in which the legitimate parties are assumed to have additional resources unavailable to the adversary (e.g., a shared private key) or the availability of better communication channel conditions (e.g., using wiretap codes). However, in data privacy problems, the sensitive data is to be protected from the same legitimate party that receives the messages and provides the utility; and hence, the above-mentioned techniques for guaranteeing data security are not applicable. Another model frequently used in the context of information-theoretic security assumes the availability of different side information at the legitimate receiver and the eavesdropper [28,29]. A DHT problem with security constraints formulated along these lines is studied in [30], where the authors propose an inner bound on the rate-error exponent-equivocation trade-off. While our model is related to that in [30] when the side information at the detector and eavesdropper coincide, there are some important differences which will be highlighted in Section 2.3.

Many different privacy measures have been considered in the literature to quantify the amount of private information leakage, such as k-anonymity [31], differential privacy (DP) [32], mutual information leakage [33,34,35], maximal leakage [36], and total variation distance [37] to count a few; see [38] for a detailed survey. Among these, mutual information between the private and revealed information (or, equivalently, the *equivocation* of private information given the revealed information) is perhaps the most commonly used measure in the information-theoretic studies of privacy. It is well known that a necessary and sufficient condition to guarantee statistical independence between two random variables is to have zero mutual information between them. Furthermore, the average information leakage measured using an arbitrary privacy measure is upper bounded by a constant multiplicative factor of that measured by mutual information [34]. It is also shown in [33] that a differentially private scheme is not necessarily private when the information leakage is measured by mutual information. This is done by constructing an example that is differentially private, yet the mutual information leakage is arbitrarily high. Mutual information-based measures have also been used in cryptographic security studies. For example, the notion of semantic security defined in [39] is shown to be equivalent to a measure based on mutual information in [40].

A rate-distortion approach to privacy is first explored by Yamamoto in [41] for a rate-constrained noiseless channel, where in addition to a distortion constraint for legitimate data, a minimum distortion requirement is enforced for the private part. Recently, there have been several works that have used distortion as a security or privacy metric in several different contexts, such as side-information privacy in discriminatory lossy source coding [42] and rate-distortion theory of secrecy systems [43,44]. More specifically, in [43], the distortion-based security measure is analyzed under a *causal disclosure assumption*, in which the data samples to be protected are causally revealed to the eavesdropper (excluding the current sample), yet the average distortion over the entire block has to satisfy a desired lower bound. This assumption ensures that distortion as a secrecy measure is more *robust* (see ([43], Section I-A)), and could in practice model scenarios in which the sensitive data to be protected is eventually available to the eavesdropper with some delay, but the protection of the current data sample is important. In this paper, we will consider both equivocation and average distortion under a causal disclosure assumption as measures of privacy. In [45], error exponent of a HT adversary is considered to be a privacy measure. This can be considered to be the opposite setting to ours, in the sense that while the goal here is to increase the error exponent under a privacy leakage constraint, the goal in [45] is to reduce the error exponent under a constraint on possible transformations that can be applied on the data.

It is instructive to compare the privacy measures considered in this paper with DP. Towards this, note that average distortion and equivocation (see Definitions 1 and 2) are “average case” privacy measures, while DP is a “worst case” measure that focuses on the statistical indistinguishability of neighboring datasets that differ in just one entry. Considering this aspect, it may appear that these privacy measures are unrelated. However, as shown in [46], there is an interesting connection between them. More specifically, the maximum conditional mutual information leakage between the revealed data *Y* and an entry in the dataset $X_{i}$ given all the other n−1 entries $X^{-i}=X^{n}\setminus \left\{X_{i}\right\}$, i.e., $I(Y;X_{i}|X^{-i})$, is sandwiched between the so-called ϵ- DP and (ϵ,δ)-DP in terms of the strength of the privacy measure, where the maximization is over all distributions $P_{X^{n}}$ on $X^{n}$ and entries i∈[1:n] ([46], Theorem 1). This implies that as a privacy measure, equivocation (equivalent to mutual information leakage) is weaker than ϵ- DP, and stronger than (ϵ,δ)-DP, at least for some probability distributions on the data. On the other hand, equivocation and average distortion are relatively well-behaved privacy measures compared to DP, and often result in clean and exact computable characterizations of the optimal trade-off for the problem at hand. Moreover, as already shown in [39,40,47,48], the trade-off resulting from “average” constraints turns out to be the same as that with more stricter constraints in many interesting cases. Hence, it is of interest to consider such average case privacy measures as a starting point for further investigation with stricter measures.

DP has been used extensively in privacy studies including those that involve learning and HT [49,50,51,52,53,54,55,56,57,58,59]. More relevant to the distributed HT problem at hand is the local differentially private model employed in [49,50,51,56], in which, depending on the privacy requirement, a certain amount of random noise is injected into the user’s data before further processing, while the utility is maximized subject to this constraint. Nevertheless, there are key differences between these models and ours. For example, in [49], the goal is to learn from differentially private “examples”, the underlying “concept” (model that maps examples to “labels”) such that the error probability in predicting the label for future examples is minimized, irrespective of the statistics of the examples. Hence, the utility in [49] is to learn an unknown model accurately, whereas our objective is to test between two known probability distributions. Furthermore, in our setting (unlike [49,50,51,56]), there is an additional requirement to satisfy in terms of the communication rate. These differences perhaps also make DP less suitable as a privacy measure in our model relative to equivocation and average distortion. On one hand, imposing a DP measure in our setting may be overly restrictive since there are only two probability distributions involved and DP is tailored for situations where the statistics of the underlying data is unknown. On the other hand, DP is also more unwieldy to analyze under a rate constraint compared to mutual information or average distortion.

The amount of private information leakage that can be tolerated depends on the specific application at hand. While it may be possible to tolerate a moderate amount of information leakage in applications like online shopping or social networks, it may no longer be the case in matters related to information sharing among government agencies or corporations. While it is obvious that maximum privacy can be attained by revealing no information, this typically comes at the cost of zero utility. On the other hand, maximum utility can be achieved by revealing all the information, but at the cost of minimum privacy. Characterizing the optimal trade-off between the utility and the minimum privacy leakage between these two extremes is a fundamental and challenging research problem.

### 1.2. Main Contributions

The main contributions of this work are as follows.

1. In Section 3, Theorem 1 (resp. Theorem 2), we establish a single-letter inner bound on the rate-error exponent-equivocation (resp. rate-error exponent-distortion) trade-off for DHT with a privacy constraint. The distortion and equivocation privacy constraints we consider, which is given in (6) and (7), respectively, are slightly stronger than what is usually considered in the literature (stated in (8) and (9), respectively).
2. Exact characterizations are obtained for some important special cases in Section 4. More specifically, a single-letter characterization of the optimal rate-error exponent-equivocation (resp. rate-error exponent-distortion) trade-off is established for:
  (a) TACI with a privacy constraint (for vanishing type I error probability constraint) in Section 4.1, Proposition 1 (resp. Proposition 2),
  (b) DHT with a privacy constraint for zero-rate compression in Section 4.2, Proposition 4 (resp. Proposition 3).
  Since the optimal trade-offs in Propositions 3 and 4 are independent of the constraint on the type I error probability, they are strong converse results in the context of HT.
3. Finally, in Section 5, we provide a counter-example showing that for a positive rate R>0, the strong converse result does not hold in general for TAI with a privacy constraint.

### 1.3. Organization

The organization of the paper is as follows. Basic notations are introduced in Section 2.1. The problem formulation and associated definitions are given in Section 2.2. Main results are presented in Section 3 to Section 5. The proofs of the results are presented either in the Appendix or immediately after the statement of the result. Finally, Section 6 concludes the paper with some open problems for future research.

## 2. Preliminaries

### 2.1. Notations

N, R and $R_{\geq 0}$ stand for the set of natural numbers, real numbers and non-negative real numbers, respectively. For $a\in R_{\geq 0}$, [a]:={i∈N,i≤a} and for a∈R, $a^{+}:=\max\left\{0,a\right\}$ (:= represents equality by definition). Calligraphic letters, e.g., A, denotes sets, while |A| and $A^{c}$ denotes its cardinality and complement, respectively. 1(·) denotes the indicator function, while O(·), o(·) and Ω(·) stands for the standard asymptotic notations of Big-O, Little-O and Big-Ω, respectively. For a real sequence ${\left\{a_{n}\right\}}_{n\in N}$ and b∈R, $a_{n}\overset{\left(n\right)}{\to }b$ represents $\lim_{n\to \infty }a_{n}=b$. Similar notations apply for asymptotic inequalities, e.g., $a_{n}\overset{\left(n\right)}{\geq }b$, means that $\lim_{n\to \infty }a_{n}\geq b$. Throughout this paper, the base of the logarithms is taken to be *e*, and whenever the range of the summation is not specified, it means summation over the entire support, e.g., $\sum_{u}$ denotes $\sum_{u\in U}$.

All the random variables (r.v.’s) considered in this paper are discrete with finite support unless specified otherwise. We denote r.v.’s, their realizations and support by upper case, lower case and calligraphic letters (e.g., *X*, *x* and X), respectively. The joint probability distribution of r.v.’s *X* and *Y* is denoted by $P_{XY}$, while their marginals are denoted by $P_{X}$ and $P_{Y}$. The set of all probability distributions with support X and X×Y are represented by P(X) and P(X×Y), respectively. For j,i∈N, the random vector $\left(X_{i},\ldots ,X_{j}\right)$, j≥i, is denoted by $X_{i}^{j}$, while $X^{j}$ stands for $\left(X_{1},\ldots ,X_{j}\right)$. Similar notation holds for the vector of realizations. X−Y−Z denotes a Markov chain relation between the r.v.’s *X*, *Y* and *Z*. $P_{P}\left(E\right)$ denotes the probability of event E with respect to the probability measure induced by distribution *P*, and $E_{P}\left[\cdot \right]$ denotes the corresponding expectation. The subscript *P* is omitted when the distribution involved is clear from the context. For two probability distributions *P* and *Q* defined on a common support, P<<Q denotes that *P* is absolutely continuous with respect to *Q*.

Following the notation in [60], for $P_{X}\in P\left(X\right)$ and δ≥0, the $P_{X}$-typical set is

$$\begin{array}{c} T_{{\left[P_{X}\right]}_{\delta }}^{n}:=\left\{x^{n}\in X^{n}:\;\left|P_{X}\left(x^{\prime }\right)-\frac{1}{n}\sum_{i=1}^{n}1\left(x_{i}=x^{\prime }\right)\right|\leq \delta ,\;\forall \;x^{\prime }\in X\right\}, \end{array}$$

and the $P_{X}$-type class (set of sequences of type or empirical distribution $P_{X}$) is $T_{P_{X}}^{n}:=T_{{\left[P_{X}\right]}_{0}}^{n}$. The set of all possible types of sequences of length *n* over an alphabet $X^{n}$ and the set of types in $T_{{\left[P_{X}\right]}_{\delta }}^{n}$ are denoted by $P^{n}\left(X\right)$ and $P_{n}\left(T_{{\left[P_{X}\right]}_{\delta }}^{n}\right)$, respectively. Similar notations apply for pairs and larger combinations of r.v.’s, e.g., $T_{{\left[P_{XY}\right]}_{\delta }}^{n}$, $T_{P_{XY}}^{n}$, $P^{n}\left(X\times Y\right)$ and $P_{n}\left(T_{{\left[P_{XY}\right]}_{\delta }}^{n}\right)$. The conditional $P_{Y|X}$ type class of a sequence $x^{n}\in X^{n}$ is

(1) $$\begin{array}{c} T_{P_{Y|X}}^{n}\left(x^{n}\right):=\left\{y^{n}:\left(x^{n},y^{n}\right)\in T_{P_{XY}}^{n}\right\}. \end{array}$$

The standard information-theoretic quantities like Kullback–Leibler (KL) divergence between distributions $P_{X}$ and $Q_{X}$, the entropy of *X* with distribution $P_{X}$, the conditional entropy of *X* given *Y* and the mutual information between *X* and *Y* with joint distribution $P_{XY}$, are denoted by $D(P_{X}||Q_{X})$, $H_{P_{X}}\left(X\right)$, $H_{P_{XY}}\left(X|Y\right)$ and $I_{P_{XY}}\left(X;Y\right)$, respectively. When the distribution of the r.v.’s involved are clear from the context, the last three quantities are denoted simply by H(X), H(X|Y) and I(X;Y), respectively. Given realizations $X^{n}=x^{n}$ and $Y^{n}=y^{n}$, $H_{e}\left(x^{n}|y^{n}\right)$ denotes the conditional empirical entropy given by

(2) $$\begin{array}{c} H_{e}\left(y^{n}|x^{n}\right):=H_{P_{\tilde{X}\tilde{Y}}}\left(\tilde{Y}|\tilde{X}\right), \end{array}$$

where $P_{\tilde{X}\tilde{Y}}$ denotes the joint type of $\left(x^{n},y^{n}\right)$. Finally, the total variation between probability distributions $P_{X}$ and $Q_{X}$ defined on the same support X is

$$\begin{array}{c} \left|\right|P_{X}-Q_{X}||:=\frac{1}{2}\sum_{x\in X}\left|P_{X}\left(x\right)-Q_{X}\left(x\right)\right|. \end{array}$$

### 2.2. Problem Formulation

**Figure 1:**
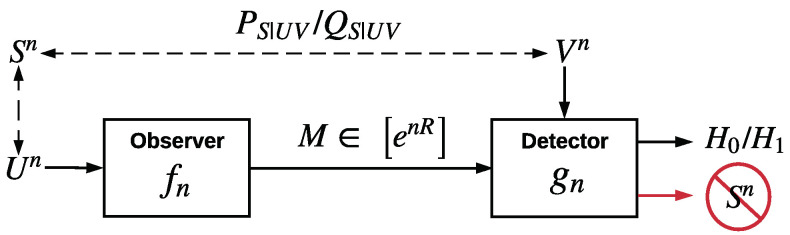
DHT with a privacy constraint.

Consider the HT setup illustrated in Figure 1, where $\left(U^{n},V^{n},S^{n}\right)$ denote *n* independent and identically distributed (i.i.d.) copies of triplet of r.v.’s (U,V,S). The observer observes $U^{n}$ and sends the message index *M* to the detector over an error-free channel, where $M∼f_{n}\left(\cdot |U^{n}\right)$ and $f_{n}:U^{n}\to P\left(M\right)$, $M=[e^{nR}]$. Given its own observation $V^{n}$, the detector performs a HT on the joint distribution of $U^{n}$ and $V^{n}$ with null hypothesis

$$\begin{array}{c} H_{0}:\left(U^{n},V^{n}\right)∼\prod_{i=1}^{n}P_{UV}, \end{array}$$

and alternate hypothesis

$$\begin{array}{c} H_{1}:\left(U^{n},V^{n}\right)∼\prod_{i=1}^{n}Q_{UV}. \end{array}$$

Let *H* and $\hat{H}$ denote the r.v.’s corresponding to the true hypothesis and the output of the HT, respectively, with support $H=\hat{H}=\left\{0,1\right\}$, where 0 denotes the null hypothesis and 1 the alternate hypothesis. Let $g_{n}:M\times V^{n}\to P\left(\hat{H}\right)$ denote the decision rule at the detector, which outputs $\hat{H}∼g_{n}\left(M,V^{n}\right)$. Then, the type I and type II error probabilities achieved by a $\left(f_{n},g_{n}\right)$ pair are given by

$$\begin{array}{c} \alpha_{n}\left(f_{n},g_{n}\right):=P\left(\hat{H}=1|H=0\right)=P_{\hat{H}}\left(1\right), \end{array}$$

and

$$\begin{array}{c} \beta_{n}\left(f_{n},g_{n}\right):=P\left(\hat{H}=0|H=1\right)=Q_{\hat{H}}\left(0\right), \end{array}$$

respectively, where

$$\begin{array}{cc} P_{\hat{H}}\left(1\right) & =\sum_{u^{n},m,v^{n}}\left[\prod_{i=1}^{n}P_{UV}\left(u_{i},v_{i}\right)\right]\;f_{n}\left(m|u^{n}\right)\;g_{n}\left(1|m,v^{n}\right), \end{array}$$

and

$$\begin{array}{cc} Q_{\hat{H}}\left(0\right) & =\sum_{u^{n},m,v^{n}}\left[\prod_{i=1}^{n}Q_{UV}\left(u_{i},v_{i}\right)\right]\;f_{n}\left(m|u^{n}\right)\;g_{n}\left(0|m,v^{n}\right). \end{array}$$

Let $P_{U^{n}V^{n}S^{n}M\hat{H}}$ and $Q_{U^{n}V^{n}S^{n}M\hat{H}}$ denote the joint distribution of $\left(U^{n},V^{n},S^{n},M,\hat{H}\right)$ under the null and alternate hypotheses, respectively. For a given type I error probability constraint ϵ, define the minimum type II error probability over all possible detectors as

(3) $$\begin{array}{cc} \; & {\bar{\beta }}_{n}\left(f_{n},\epsilon \right):=\underset{g_{n}}{\inf}\beta_{n}\left(f_{n},\;g_{n}\right),\\ \; & \mathrm{such}\;\mathrm{that}\alpha_{n}\left(f_{n},\;g_{n}\right)\leq \epsilon . \end{array}$$

The performance of HT is measured by the error exponent achieved by the test for a given constraint ϵ on the type I error probability, i.e., ${lim inf}_{n\to \infty }-\frac{1}{n}\log\left({\bar{\beta }}_{n}\left(f_{n},\epsilon \right)\right)$. Although the goal of the detector is to maximize the error exponent achieved for the HT, it is also curious about the latent r.v. $S^{n}$ that is correlated with $U^{n}$. $S^{n}$ is referred to as the *private* part of $U^{n}$, which is distributed i.i.d. according to the joint distribution $P_{SUV}$ and $Q_{SUV}$ under the null and alternate hypothesis, respectively. It is desired to keep the private part as concealed as possible from the detector. We consider two measures of privacy for $S^{n}$ at the detector. The first is the *equivocation* defined as $H(S^{n}|M,V^{n})$. The second one is the *average distortion* between $S^{n}$ and its reconstruction ${\hat{S}}^{n}$ at the detector, measured according to an arbitrary bounded additive distortion metric $d:S\times \hat{S}\to \left[0,D_{m}\right]$ with multi-letter distortion defined as

(4) $$\begin{array}{c} d\left(s^{n},{\hat{s}}^{n}\right):=\sum_{i=1}^{n}d\left(s_{i},{\hat{s}}_{i}\right). \end{array}$$

We will assume the *causal disclosure* assumption, i.e., ${\hat{S}}_{i}$ is a function of $S^{i-1}$ in addition to $\left(M,V^{n}\right)$. The goal is to ensure that the error exponent for HT is maximized, while satisfying the constraints on the type I error probability ϵ and the privacy of $S^{n}$. In the sequel, we study the trade-off between the rate, error exponent (henceforth also referred to simply as the error exponent) and privacy achieved in the above setting. Before delving into that, a few definitions are in order.

**Definition** **1.** *For a given type I error probability constraint ϵ, a rate-error exponent-distortion tuple $\left(R,\kappa ,\Delta_{0},\Delta_{1}\right)$ is *achievable*, if there exists a sequence of encoding and decoding functions $f_{n}:U^{n}\to P\left(M\right)$, and $g_{n}:M\times V^{n}\to P\left(\hat{H}\right)$ such that*

(5) $$\begin{array}{cc} \underset{n\to \infty }{lim inf}\frac{-\log\left({\bar{\beta }}_{n}\left(f_{n},\epsilon \right)\right)}{n} & \geq \kappa , \end{array}$$

*and for any γ>0, there exists an $n_{0}\in N$ such that*

(6) $$\begin{array}{cc} \underset{{\left\{g_{i,n}^{\left(r\right)}\right\}}_{i=1}^{n}}{\inf}E\left[d\left(S^{n},{\hat{S}}^{n}\right)|H=j\right] & \geq n\Delta_{j}-\gamma ,\;\forall \;n\geq n_{0},\;j=0,1, \end{array}$$

*where ${\hat{S}}_{i}∼g_{i,n}^{\left(r\right)}\left(\cdot |M,V^{n},S^{i-1}\right)$, and $g_{i,n}^{\left(r\right)}:\left[e^{nR}\right]\times V^{n}\times S^{i-1}\to P\left({\hat{S}}_{i}\right)$ denotes an arbitrary stochastic reconstruction map at the detector. The rate-error exponent-distortion region $R_{d}\left(\epsilon \right)$ is the closure of the set of all such achievable $\left(R,\kappa ,\Delta_{0},\Delta_{1}\right)$ tuples for a given ϵ.*

**Definition** **2.** *For a given type I error probability constraint ϵ, a rate-error exponent-equivocation (It is well known that equivocation as a privacy measure is a special case of average distortion under the causal disclosure assumption and log-loss distortion metric [43]. However, we provide a separate definition of the rate-error exponent-equivocation region for completeness.) $\left(R,\kappa ,\Lambda_{0},\Lambda_{1}\right)$ tuple is *achievable*, if there exists a sequence of encoding and decoding functions $f_{n}:U^{n}\to P\left(M\right)$ and $g_{n}:\left[e^{nR}\right]\times V^{n}\to P\left(\hat{H}\right)$ such that (5) is satisfied, and for any γ>0, there exists a $n_{0}\in N$ such that*

(7) $$\begin{array}{c} H\left(S^{n}|M,V^{n},H=i\right)\geq n\Lambda_{i}-\gamma ,\;\forall \;n\geq n_{0},\;i\in \left\{0,1\right\}. \end{array}$$

*The rate-error exponent-equivocation region $R_{e}\left(\epsilon \right)$ is the closure of the set of all such achievable $\left(R,\kappa ,\Lambda_{0},\Lambda_{1}\right)$ tuples for a given ϵ.*

Please note that the privacy measures considered in (6) and (7) are stronger than

(8) $$\begin{array}{cc} \; & \underset{n\to \infty }{lim inf}\underset{{\left\{g_{i,n}^{\left(r\right)}\right\}}_{i=1}^{n}}{\inf}E\left[\frac{1}{n}d\left(S^{n},{\hat{S}}^{n}\right)|H=i\right]\geq \Delta_{i},\;i=0,1, \end{array}$$

(9) $$\begin{array}{cc} \mathrm{and}\; & \underset{n\to \infty }{lim inf}\frac{1}{n}H\left(S^{n}|M,V^{n},H=i\right)\geq \Lambda_{i},\;i=0,1, \end{array}$$

respectively. To see this for the equivocation privacy measure, note that if $H\left(S^{n}|M,V^{n},H=i\right)=n\Lambda_{i}^{*}-n^{a}$, i=0,1, for some a∈(0,1), then an equivocation pair $\left(\Lambda_{0}^{*},\Lambda_{1}^{*}\right)$ is achievable under the constraint given in (9), while it is not achievable under the constraint given in (7).

### 2.3. Relation to Previous Work

Before stating our results, we briefly highlight the differences between our system model and the ones studied in [27,30]. In [27], the observer applies a privacy mechanism to the data before releasing it to the transmitter, which performs further encoding prior to transmission to the detector. More specifically, the observer checks if $U^{n}\in T_{{\left[P_{U}\right]}_{\delta }}^{n}$ and if successful, sends the output of a memoryless privacy mechanism applied to $U^{n}$, to the transmitter. Otherwise, it outputs a *n*-length zero-sequence. The privacy mechanism plays the role of randomizing the data (or adding noise) to achieve the desired privacy. Such randomized privacy mechanisms are popular in privacy studies, and have been used in [25,26,61]. In our model, the tasks of coding for privacy and compression are done jointly by using all the available data samples $U^{n}$. Also, while we consider the equivocation (and average distortion) between the revealed information and the private part as the privacy measure, in [27], the mutual information between the observer’s observations and the output of the memoryless mechanism is the privacy measure. As a result of these differences, there exist some points in the rate-error exponent-privacy trade-off that are achievable in our model, but not in [27]. For instance, a perfect privacy condition $\Lambda_{0}=0$ for testing against independence in ([27], Theorem 2) would imply that the error exponent is also zero, since the output of the memoryless mechanism has to be independent of the observer’s observations (under both hypotheses). However, as we later show in Example 2, a positive error exponent is achievable while guaranteeing perfect privacy in our model.

On the other hand, the difference between our model and [30] arises from the difference in the privacy constraint as well as the privacy measure. Specifically, the goal in [30] is to keep $U^{n}$ private from an illegitimate eavesdropper, while the objective here is to keep a r.v. $S^{n}$ that is correlated with $U^{n}$ private from the detector. Also, we consider the more general average distortion (under causal disclosure) as a privacy measure, in addition to equivocation in [30]. Moreover, as already noted, the equivocation privacy constraint in (7) is more stringent than (9) that is considered in [30]. To satisfy the distortion requirement or the stronger equivocation privacy constraint in (7), we require that the a posteriori probability distribution of $S^{n}$ given the observations $\left(M,V^{n}\right)$ at the detector is close in some sense to a desired “target" memoryless distribution. To achieve this, we use a stochastic encoding scheme to induce the necessary randomness for $S^{n}$ at the detector, which to the best of our knowledge has not been considered previously in the context of DHT. Consequently, the analysis of the type I and type II error probabilities and privacy achieved are novel. Another subtle yet important difference is that the marginal distributions of $U^{n}$ and the side information at the eavesdropper are assumed to be the same under the null and alternate hypotheses in [30], which is not the case here. This necessitates separate analysis for the privacy achieved under the two hypotheses.

Next, we state some supporting results that will be useful later for proving the main results.

### 2.4. Supporting Results

Let

(10) $$\begin{array}{c} g_{A_{n}}^{\left(d\right)}\left(m,v^{n}\right)=1\left(\left(m,v^{n}\right)\in A_{n}^{c}\right) \end{array}$$

denote a deterministic detector with acceptance region $A_{n}\subseteq \left[e^{nR}\right]\times V^{n}$ for $H_{0}$ and $A_{n}^{c}$ for $H_{1}$. Then, the type I and type II error probabilities are given by

(11) $$\begin{array}{cc} \alpha_{n}\left(f_{n},g_{n}\right) & :=P_{MV^{n}}\left(A_{n}^{c}\right)=E_{P}\left[1\left(\left(M,V^{n}\right)\in A_{n}^{c}\right)\right], \end{array}$$

(12) $$\begin{array}{cc} \beta_{n}\left(f_{n},g_{n}\right) & :=Q_{MV^{n}}\left(A_{n}\right)=E_{Q}\left[1\left(\left(M,V^{n}\right)\in A_{n}\right)\right]. \end{array}$$

**Lemma** **1.** *Any error exponent that is achievable is also achievable by a deterministic detector of the form given in (10) for some $A_{n}\subseteq \left[e^{nR}\right]\times V^{n}$, where $A_{n}$ and $A_{n}^{c}$ denote the acceptance regions for $H_{0}$ and $H_{1}$, respectively.*

The proof of Lemma 1 is given in Appendix A for completeness. Due to Lemma 1, henceforth we restrict our attention to a deterministic $g_{n}$ as given in (10).

The next result shows that without loss of generality (w.l.o.g), it is also sufficient to consider $g_{i,n}^{\left(r\right)}$ (in Definition 1) to be a deterministic function of the form

(13) $$\begin{array}{c} g_{i,n}^{\left(r\right)}={\left\{{\bar{\phi }}_{i,n}\left(\cdot ,\cdot ,\cdot \right)\right\}}_{i=1}^{n} \end{array}$$

for the minimization in (6), where ${\bar{\phi }}_{i,n}:M\times V^{n}\times S^{i-1}\to \hat{S}$, i∈[n], denotes an arbitrary deterministic function.

**Lemma** **2.** *The infimum in (6) is achieved by a deterministic function $g_{i,n}^{\left(r\right)}$ as given in (13), and hence it is sufficient to restrict to such deterministic $g_{i,n}^{\left(r\right)}$ in (6).*

The proof of Lemma 2 is given in Appendix B. Next, we state some lemmas that will be handy for upper bounding the amount of privacy leakage in the proofs of the main results stated below. The following one is a well-known result proved in [60] that upper bounds the difference in entropy of two r.v.’s (with a common support) in terms of the total variation distance between their probability distributions.

**Lemma** **3.** *([60], Lemma 2.7) Let $P_{X}$ and $Q_{X}$ be distributions defined on a common support X and let $\rho :=\left|\right|P_{X}-Q_{X}\left|\right|$. Then, for $\rho \leq \frac{1}{4}$*

$$\begin{array}{c} |H_{P_{X}}-H_{Q_{X}}|\leq -2\rho \log\left(\frac{2\rho }{\left|X\right|}\right). \end{array}$$

The next lemma will be handy in proving Theorems 1 and 2, Proposition 3 and the counter-example for strong converse presented in Section 5.

**Lemma** **4.** *Let $\left(X^{n},Y^{n}\right)$ denote n i.i.d. copies of r.v.’s (X,Y), and $P_{X^{n}Y^{n}}=\prod_{i=1}^{n}P_{XY}$ and $Q_{X^{n}Y^{n}}=\prod_{i=1}^{n}Q_{XY}$ denote two joint probability distributions on $\left(X^{n},Y^{n}\right)$. For δ>0, define*

(14) $$\begin{array}{c} \Pi \left(x^{n},\delta ,P_{X}\right):=1\left(x^{n}\notin T_{{\left[P_{X}\right]}_{\delta }}^{n}\right). \end{array}$$

*If $P_{X}\neq Q_{X}$, then for δ>0 sufficiently small, there exists $\bar{\delta }>0$ and $n_{0}\left(\delta ,|X|,|Y|\right)\in N$ such that for all $n\geq n_{0}\left(\delta ,|X|,|Y|\right)$,*

(15) $$\begin{array}{c} ∥Q_{Y^{n}}\left(\cdot \right)-Q_{Y^{n}|\Pi \left(X^{n},\delta ,P_{X}\right)}\left(\cdot |1\right)∥\leq e^{-n\bar{\delta }}. \end{array}$$

*If $P_{X}=Q_{X}$, then for any δ>0, there exists $\bar{\delta }>0$ and $n_{0}\left(\delta ,|X|,|Y|\right)\in N$ such that for all $n\geq n_{0}\left(\delta ,|X|,|Y|\right)$,*

(16) $$\begin{array}{cc} ∥Q_{Y^{n}}\left(\cdot \right)-Q_{Y^{n}|\Pi \left(X^{n},\delta ,P_{X}\right)}\left(\cdot |0\right)∥ & \leq e^{-n\bar{\delta }}, \end{array}$$

*Also, for any δ>0, there exists $\bar{\delta }>0$ and $n_{0}\left(\delta ,|X|,|Y|\right)\in N$ such that for all $n\geq n_{0}\left(\delta ,|X|,|Y|\right)$,*

(17) $$\begin{array}{cc} ∥P_{Y^{n}}\left(\cdot \right)-P_{Y^{n}|\Pi \left(X^{n},\delta ,P_{X}\right)}\left(\cdot |0\right)∥ & \leq e^{-n\bar{\delta }}. \end{array}$$

**Proof.**  The proof is presented in Appendix C. ☐

In the next section, we establish an inner bound on $R_{e}\left(\epsilon \right)$ and $R_{d}\left(\epsilon \right)$.

## 3. Main Results

The following two theorems are the main results of this paper providing inner bounds for $R_{e}\left(\epsilon \right)$ and $R_{d}\left(\epsilon \right)$, respectively.

**Theorem** **1.** *For ϵ∈(0,1), $\left(R,\kappa ,\Lambda_{0},\Lambda_{1}\right)\in R_{e}\left(\epsilon \right)$ if there exists an auxiliary r.v. W, such that (V,S)−U−W, and*

(18) $$\begin{array}{cc} R & \geq I_{P}\left(W;U|V\right), \end{array}$$

(19) $$\begin{array}{cc} \kappa & \leq \kappa^{*}\left(P_{W|U},R\right), \end{array}$$

(20) $$\begin{array}{cc} \Lambda_{0} & \leq H_{P}\left(S|W,V\right), \end{array}$$

(21) $$\begin{array}{cc} \Lambda_{1} & \leq 1\left(P_{U}=Q_{U}\right)\;H_{Q}\left(S|W,V\right)+1\left(P_{U}\neq Q_{U}\right)\;H_{Q}\left(S|V\right), \end{array}$$

*where*

$$\begin{array}{cc} \kappa^{*}\left(P_{W|U},R\right) & :=\min\left(E_{1}\left(P_{W|U}\right),\;E_{2}\left(R,P_{W|U}\right)\right), \end{array}$$

(22) $$\begin{array}{cc} E_{1}\left(P_{W|U}\right) & :=\min_{P_{\tilde{U}\tilde{V}\tilde{W}}\in L_{1}\left(P_{UW},P_{VW}\right)}D(P_{\tilde{U}\tilde{V}\tilde{W}}\left|\right|Q_{UV}P_{W|U}), \end{array}$$

(23) $$\begin{array}{cc} E_{2}\left(R,P_{W|U}\right) & :=\left\{\begin{array}{cc} \begin{array}{c} \min\\ P_{\tilde{U}\tilde{V}\tilde{W}}\in L_{2}\left(P_{UW},P_{V}\right) \end{array}\;D(P_{\tilde{U}\tilde{V}\tilde{W}}\left|\right|Q_{UV}P_{W|U})+\left(R-I_{P}\left(U;W|V\right)\right), & ifI_{P}\left(U;W\right)>R,\\ \infty , & otherwise, \end{array}\right.\\ L_{1}\left(P_{UW},P_{VW}\right) & :=\{P_{\tilde{U}\tilde{V}\tilde{W}}\in P\left(U\times V\times W\right):P_{\tilde{U}\tilde{W}}=P_{UW},\;P_{\tilde{V}\tilde{W}}=P_{VW}\},\\ L_{2}\left(P_{UW},P_{V}\right) & :=\{P_{\tilde{U}\tilde{V}\tilde{W}}\in P\left(U\times V\times W\right):P_{\tilde{U}\tilde{W}}=P_{UW},\;P_{\tilde{V}}=P_{V},\;H_{P}\left(W|V\right)\leq H\left(\tilde{W}|\tilde{V}\right)\},\\ P_{SUVW} & :=P_{SUV}P_{W|U},\;andQ_{SUVW}:=Q_{SUV}P_{W|U}. \end{array}$$

**Theorem** **2.** *For a given bounded additive distortion measure d(·,·) and ϵ∈(0,1), $\left(R,\kappa ,\Delta_{0},\Delta_{1}\right)\in R_{d}\left(\epsilon \right)$ if there exist an auxiliary r.v. W and deterministic functions $\phi :W\times V\to \hat{S}$ and $\phi^{\prime }:V\to \hat{S}$, such that (V,S)−U−W and (18) and (19),*

(24) $$\begin{array}{cc} \Delta_{0} & \leq \min_{\phi (\cdot ,\cdot )}E_{P}\left[d\left(S,\phi (W,V)\right)\right], \end{array}$$

(25) $$\begin{array}{cc} \mathrm{and}\;\Delta_{1} & \leq 1\left(P_{U}=Q_{U}\right)\;\min_{\phi (\cdot ,\cdot )}E_{Q}\left[d\left(S,\phi (W,V)\right)\right]+1\left(P_{U}\neq Q_{U}\right)\;\min_{\phi^{\prime }\left(\cdot \right)}E_{Q}\left[d\left(S,\phi^{\prime }\left(V\right)\right)\right], \end{array}$$

*are satisfied, where $P_{SUVW}$ and $Q_{SUVW}$ are as defined in Theorem 1.*

The proof of Theorems 1 and 2 is given in Apppendix Appendix D. While the rate-error exponent trade-off in Theorems 1 and 2 is the same as that achieved by the Shimokawa-Han-Amari (SHA) scheme [7], the coding strategy achieving it is different due to the requirement of the privacy constraint. As mentioned above, in order to obtain a single-letter lower bound for the achievable distortion (and achievable equivocation) of the private part at the detector, it is required that the a posteriori probability distribution of $S^{n}$ given the observations $\left(M,V^{n}\right)$ at the detector is close in some sense to a desired “target” memoryless distribution. For this purpose, we use the so-called likelihood encoder [62,63] (at the observer) in our achievability scheme. The likelihood encoder is a stochastic encoder that induces the necessary randomness for $S^{n}$ at the detector, and to the best of our knowledge has not been used before in the context of DHT. The analysis of the type I and type II error probabilities and the privacy achieved by our scheme is novel and involves the application of the well-known *channel resolvability* or *soft-covering lemma* [62,64,65]. Properties of the total variation distance between probability distributions mentioned in [43] play a key role in this analysis. The analysis also reveals the interesting fact that the coding schemes in Theorems 1 and 2, although quite different from the SHA scheme, achieves the same lower bound on the error exponent.

Theorems 1 and 2 provide single-letter inner bounds on $R_{d}\left(\epsilon \right)$ and $R_{e}\left(\epsilon \right)$, respectively. A complete computable characterization of these regions would require a matching converse. This is a hard problem, since such a characterization is not available even for the DHT problem without a privacy constraint, in general (see [5]). However, it is known that a single-letter characterization of the rate-error exponent region exists for the special case of TACI [11]. In the next section, we show that TACI with a privacy constraint also admits a single-letter characterization, in addition to other optimality results.

## 4. Optimality Results for Special Cases

### 4.1. TACI with a Privacy Constraint

Assume that the detector observes two discrete memoryless sources $Y^{n}$ and $Z^{n}$, i.e., $V^{n}=\left(Y^{n},Z^{n}\right)$. In TACI, the detector tests for the conditional independence of *U* and *Y*, given *Z*. Thus, the joint distribution of the r.v.’s under the null and alternate hypothesis are given by

(26a) $$H_{0}:\;P_{SUYZ}:=P_{S|UYZ}P_{U|Z}P_{Y|UZ}P_{Z},$$

and

(26b) $$H_{1}:\;Q_{SUYZ}:=Q_{S|UYZ}P_{U|Z}P_{Y|Z}P_{Z},$$

respectively.

Let $R_{e}$ and $R_{d}$ denote the rate-error exponent-equivocation and rate-error exponent-distortion regions, respectively, for the case of vanishing type I error probability constraint, i.e.,

$$\begin{array}{cc} R_{e} & :=\lim_{\epsilon \to 0}R_{e}\left(\epsilon \right)andR_{d}:=\lim_{\epsilon \to 0}R_{d}\left(\epsilon \right). \end{array}$$

Assume that the privacy constraint under the alternate hypothesis is inactive. Thus, we are interested in characterizing the set of all tuples $\left(R,\kappa ,\Lambda_{0},\Lambda_{1}\right)\in R_{e}$ and $\left(R,\kappa ,\Delta_{0},\Delta_{1}\right)\in R_{d}$, where

(27) $$\begin{array}{cc} \Lambda_{1} & \leq \Lambda_{min}:=H_{Q}\left(S|U,Y,Z\right),\\ \mathrm{and}\;\Delta_{1} & \leq \Delta_{min}:=\min_{\phi (u,y,z)}E_{Q}\left[d\left(S,\phi (U,Y,Z)\right)\right]. \end{array}$$

Please note that $\Lambda_{min}$ and $\Delta_{min}$ correspond to the equivocation and average distortion of $S^{n}$ at the detector, respectively, when $U^{n}$ is available directly at the detector under the alternate hypothesis. The above assumption is motivated by scenarios, in which the observer is more eager to protect $S^{n}$ when there is a correlation between its own observation and that of the detector, such as the online shopping portal example mentioned in Section 1. In that example, $U^{n}$, $S^{n}$ and $Y^{n}$ corresponds to shopping behavior, more information about the customer, and customers data available to the shopping portal, respectively.

For the above-mentioned case, we have the following results.

**Proposition** **1.** *For the HT given in (26), $\left(R,\kappa ,\Lambda_{0},\Lambda_{min}\right)\in R_{e}$ if and only if there exists an auxiliary r.v. W, such that (Z,Y,S)−U−W, and*

(28) $$\begin{array}{cc} \kappa & \leq I_{P}\left(W;Y|Z\right), \end{array}$$

(29) $$\begin{array}{cc} R & \geq I_{P}\left(W;U|Z\right), \end{array}$$

(30) $$\begin{array}{cc} \Lambda_{0} & \leq H_{P}\left(S|W,Z,Y\right), \end{array}$$

*for some joint distribution of the form $P_{SUYZW}:=P_{SUYZ}P_{W|U}$.*

**Proof.**  For TACI, the inner bound in Theorem 1 yields that for ϵ∈(0,1), $\left(R,\kappa ,\Lambda_{0},\Lambda_{1}\right)\in R_{e}\left(\epsilon \right)$ if there exists an auxiliary r.v. *W*, such that (Y,Z,S)−U−W, and

(31) $$\begin{array}{cc} R & \geq I_{P}\left(W;U|Y,Z\right), \end{array}$$

(32) $$\begin{array}{cc} \kappa & \leq \kappa^{*}\left(P_{W|U},R\right), \end{array}$$

(33) $$\begin{array}{cc} \Lambda_{0} & \leq H_{P}\left(S|W,Y,Z\right), \end{array}$$

(34) $$\begin{array}{cc} \Lambda_{1} & \leq H_{Q}\left(S|W,Y,Z\right), \end{array}$$

where

$$\begin{array}{cc} \; & \kappa^{*}\left(P_{W|U},R\right):=\min\left(E_{1}\left(P_{W|U}\right),\;E_{2}\left(R,P_{W|U}\right)\right), \end{array}$$

(35) $$\begin{array}{cc} \; & E_{1}\left(P_{W|U}\right):=\min_{P_{\tilde{U}\tilde{Y}\tilde{Z}\tilde{W}}\in L_{1}\left(P_{UW},P_{YZW}\right)}D(P_{\tilde{U}\tilde{Y}\tilde{Z}\tilde{W}}\left|\right|Q_{UYZ}P_{W|U}),\\ \; & E_{2}\left(R,P_{W|U}\right) \end{array}$$

(36) $$\begin{array}{ccc} \; & :=\left\{\begin{array}{cc} \begin{array}{c} \min\\ P_{\tilde{U}\tilde{Y}\tilde{Z}\tilde{W}}\in L_{2}\left(P_{UW},P_{YZ}\right) \end{array}\;D(P_{\tilde{U}\tilde{Y}\tilde{Z}\tilde{W}}\left|\right|Q_{UYZ}P_{W|U})+\left(R-I_{P}\left(U;W|Y,Z\right)\right), & ifI_{P}\left(U;W\right)>R,\\ \infty , & \mathrm{otherwise}, \end{array}\right.\\ \; & L_{1}\left(P_{UW},P_{YZW}\right):=\left\{P_{\tilde{U}\tilde{Y}\tilde{Z}\tilde{W}}\in P\left(U\times Y\times Z\times W\right):P_{\tilde{U}\tilde{W}}=P_{UW},\;P_{\tilde{Y}\tilde{Z}\tilde{W}}=P_{YZW}\right\},\\ \; & L_{2}\left(P_{UW},P_{YZ}\right):=\left\{P_{\tilde{U}\tilde{Y}\tilde{Z}\tilde{W}}\in P\left(U\times Y\times Z\times W\right):P_{\tilde{U}\tilde{W}}=P_{UW},\;P_{\tilde{Y}\tilde{Z}}=P_{YZ},\;H_{P}\left(W|Y,Z\right)\leq H\left(\tilde{W}|\tilde{Y}\tilde{Z}\right)\right\},\\ \; & P_{SUYZW}:=P_{SUYZ}P_{W|U},\;Q_{SUYZW}:=Q_{S|YZ}P_{U|Z}P_{Y|Z}P_{Z}P_{W|U}. & \; \end{array}$$

Please note that since (Y,Z,S)−U−W, we have

(37) $$\begin{array}{c} I_{P}\left(W;U\right)\geq I_{P}\left(W;U|Y,Z\right). \end{array}$$

Let $B^{\prime }:=\left\{P_{W|U}:I_{P}\left(U;W|Z\right)\leq R\right\}$. Then, for $P_{W|U}\in B^{\prime }$, we have,

$$\begin{array}{cc} E_{1}\left(R,P_{W|U}\right) & =\min_{P_{\tilde{U}\tilde{Y}\tilde{Z}\tilde{W}}\in L_{1}\left(P_{UW},P_{YZW}\right)}D(P_{\tilde{U}\tilde{Y}\tilde{Z}\tilde{W}}\left|\right|Q_{UYZ}P_{W|U})=I_{P}\left(Y;W|Z\right),\\ E_{2}\left(R,P_{W|U}\right) & \geq I_{P}\left(U;W|Z\right)-I_{P}\left(U;W|Y,Z\right)=I_{P}\left(Y;W|Z\right). \end{array}$$

Hence,

(38) $$\begin{array}{c} \kappa^{*}\left(P_{W|U},R\right)\geq I_{P}\left(Y;W|Z\right). \end{array}$$

By noting that $\Lambda_{min}\leq H_{Q}\left(S|W,Y,Z\right)$ (by the data processing inequality), we have shown that for $\Lambda_{1}\leq \Lambda_{min}$, $\left(R,\kappa ,\Lambda_{0},\Lambda_{1}\right)\in R_{e}$ if (28)–(30) are satisfied. This completes the proof of achievability.

*Converse:* Let $\left(R,\kappa ,\Lambda_{0},\Lambda_{1}\right)\in R_{e}$. Let *T* be a r.v. uniformly distributed over [n] and independent of all the other r.v.’s $\left(U^{n},Y^{n},Z^{n},S^{n},M\right)$. Define an auxiliary r.v. $W:=(W_{T},T)$, where $W_{i}:=\left(M,Y^{i-1},S^{i-1},Z^{i-1},Z_{i+1}^{n}\right)$, i∈[n]. Then, we have for sufficiently large *n* that

(39) $$\begin{array}{cc} nR & \geq H_{P}\left(M\right)\geq H_{P}\left(M|Z^{n}\right)\geq I_{P}\left(M;U^{n}|Z^{n}\right)\\ \; & =\sum_{i=1}^{n}I_{P}\left(M;U_{i}|U^{i-1},Z^{n}\right) \end{array}$$

(40) $$\begin{array}{cc} \; & =\sum_{i=1}^{n}I_{P}\left(M,U^{i-1},Z^{i-1},Z_{i+1}^{n};U_{i}|Z_{i}\right)\\ \; & =\sum_{i=1}^{n}I_{P}\left(M,U^{i-1},Z^{i-1},Z_{i+1}^{n},Y^{i-1},S^{i-1};U_{i}|Z_{i}\right)\\ \; & \geq \sum_{i=1}^{n}I_{P}\left(M,Z^{i-1},Z_{i+1}^{n},Y^{i-1},S^{i-1};U_{i}|Z_{i}\right)\\ \; & =\sum_{i=1}^{n}I_{P}\left(W_{i};U_{i}|Z_{i}\right)=nI_{P}\left(W_{T};U_{T}|Z_{T},T\right) \end{array}$$

(41) $$\begin{array}{cc} \; & =nI_{P}\left(W_{T},T;U_{T}|Z_{T}\right) \end{array}$$

(42) $$\begin{array}{ccc} \; & =nI_{P}\left(W;U|Z\right). & \; \end{array}$$

Here, (39) follows since the sequences $\left(U^{n},Z^{n}\right)$ are memoryless; (40) follows since $\left(Y^{i-1},S^{i-1}\right)-\left(M,U^{i-1},Z^{n}\right)-U_{i}$ form a Markov chain; and, (41) follows from the fact that *T* is independent of all the other r.v.’s.

The equivocation of $S^{n}$ under the null hypothesis can be bounded as follows.

$$\begin{array}{cc} H(S^{n}|M,Y^{n},Z^{n},H=0) & =\sum_{i=1}^{n}H\left(S_{i}|M,S^{i-1},Y^{n},Z^{n},H=0\right) \end{array}$$

(43) $$\begin{array}{cc} \; & \leq \sum_{i=1}^{n}H\left(S_{i}|M,Y^{i-1},S^{i-1},Z^{i-1},Z_{i+1}^{n},Y_{i},Z_{i},H=0\right)\\ \; & =\sum_{i=1}^{n}H\left(S_{i}|W_{i},Y_{i},Z_{i},H=0\right)\\ \; & =nH(S_{T}|W_{T},Y_{T},Z_{T},T,H=0) \end{array}$$

(44) $$\begin{array}{ccc} \; & =nH_{P}\left(S|W,Y,Z\right), & \; \end{array}$$

where $P_{SUYZW}=P_{SUYZ}P_{W|U}$ for some conditional distribution $P_{W|U}$. In (43), we used the fact that conditioning reduces entropy.

Finally, we prove the upper bound on κ. For any encoding function $f_{n}$ and decision region $A_{n}\subseteq M\times Y^{n}\times Z^{n}$ for $H_{0}$ such that $\epsilon_{n}\to 0$, we have,

(45) $$\begin{array}{cc} D\left(P_{MY^{n}Z^{n}}\left|\right|Q_{MY^{n}Z^{n}}\right) & \geq P_{MY^{n}Z^{n}}\left(A_{n}\right)\log\left(\frac{P_{MY^{n}Z^{n}}\left(A_{n}\right)}{Q_{MY^{n}Z^{n}}\left(A_{n}\right)}\right)+P_{MY^{n}Z^{n}}\left(A_{n}^{c}\right)\log\left(\frac{P_{MY^{n}Z^{n}}\left(A_{n}^{c}\right)}{Q_{MY^{n}Z^{n}}\left(A_{n}^{c}\right)}\right)\\ \; & \geq -H\left(\epsilon_{n}\right)-\left(1-\epsilon_{n}\right)\log\left({\bar{\beta }}_{n}\left(f_{n},\epsilon_{n}\right)\right). \end{array}$$

Here, (45) follows from the log-sum inequality [60]. Thus,

$$\begin{array}{cc} \underset{n\to \infty }{lim sup}\frac{-\log\left({\bar{\beta }}_{n}\left(f_{n},\epsilon_{n}\right)\right)}{n} & \leq \underset{n\to \infty }{lim sup}\frac{1}{n}D\left(P_{MY^{n}Z^{n}}\left|\right|Q_{MY^{n}Z^{n}}\right) \end{array}$$

(46) $$\begin{array}{cc} \; & =\underset{n\to \infty }{lim sup}\;\frac{1}{n}I_{P}\left(M;Y^{n}|Z^{n}\right) \end{array}$$

(47) $$\begin{array}{ccc} \; & =H_{P}\left(Y|Z\right)-\underset{n\to \infty }{lim inf}\frac{1}{n}H_{P}\left(Y^{n}|M,Z^{n}\right), & \; \end{array}$$

where (46) follows since $Q_{MY^{n}Z^{n}}=P_{MZ^{n}}P_{Y^{n}|Z^{n}}$. The last term can be single-letterized as follows:

(48) $$\begin{array}{ccc} H_{P}\left(Y^{n}|M,Z^{n}\right) & =\sum_{i=1}^{n}H_{P}\left(Y_{i}|Y^{i-1},M,Z^{n}\right)\\ \; & \geq \sum_{i=1}^{n}H_{P}\left(Y_{i}|Y^{i-1},S^{i-1},M,Z^{n}\right)\\ \; & =\sum_{i=1}^{n}H_{P}\left(Y_{i}|Z_{i},W_{i}\right)\\ \; & =nH_{P}\left(Y_{T}|Z_{T},W_{T},T\right)\\ \; & =nH_{P}\left(Y|Z,W\right). & \; \end{array}$$

Substituting (48) in (47), we obtain

(49) $$\begin{array}{c} \kappa \leq I_{P}\left(Y;W|Z\right). \end{array}$$

Also, note that (Z,Y)−U−W holds. To see this, note that $\left(U_{i},Y_{i},Z_{i},S_{i}\right)$ are i.i.d across i∈[n]. Hence, any information in $W_{i}$ on $\left(Y_{i},Z_{i},S_{i}\right)$ is only through *M* as a function of $U_{i}$, and so given $U_{i}$, $W_{i}$ is independent of $\left(Y_{i},Z_{i},S_{i}\right)$. The above Markov chain then follows from the fact that *T* is independent of $\left(U^{n},Y^{n},Z^{n},S^{n},M\right)$. This completes the proof of the converse and the theorem. ☐

Next, we state the result for TACI with a distortion privacy constraint, where the distortion is measured using an arbitrary distortion measure d(·,·). Let $\Delta_{min}:=\min_{\phi (u,y,z)}E_{Q}\left[d\left(S,\phi (U,Y,Z)\right)\right]$.

**Proposition** **2.** *For the HT given in (26), $\left(R,\kappa ,\Delta_{0},\Delta_{min}\right)\in R_{d}$ if and only if there exist an auxiliary r.v. W and a deterministic function $\phi :W\times Y\times Z\to \hat{S}$ such that*

(50) $$\begin{array}{cc} R & \geq I_{P}\left(W;U|Z\right), \end{array}$$

(51) $$\begin{array}{cc} \kappa & \leq I_{P}\left(W;Y|Z\right), \end{array}$$

(52) $$\begin{array}{cc} \Delta_{0} & \leq \min_{\phi (\cdot ,\cdot ,\cdot )}E_{P}\left[d\left(S,\phi (W,Y,Z)\right)\right], \end{array}$$

*for some $P_{SUYZW}$ as defined in Proposition 1.*

**Proof.**  The proof of achievability follows from Theorem 2, similarly to the way Proposition 1 is obtained from Theorem 1. Hence, only differences will be highlighted. Similar to the inequality $\Lambda_{min}\leq H_{Q}\left(S|U,Y,Z\right)$ in the proof of Proposition 1, we need to prove the inequality $\Delta_{min}\leq E_{Q}\left[d\left(S,\phi (W,Y,Z)\right)\right]$, where $Q_{SUYZW}:=Q_{SUYZ}P_{W|U}$ for some conditional distribution $P_{W|U}$. This can be shown as follows:

(53) $$\begin{array}{ccc} \; & \min_{\phi (\cdot ,\cdot ,\cdot )}E_{Q}\left[d\left(S,\phi (W,Y,Z)\right)\right]\\ \; & =\sum_{u,y,z}Q_{UYZ}\left(u,y,z\right)\;\sum_{w}P_{W|U}\left(w|u\right)\;\min_{\phi (w,y,z)}\;\sum_{s}Q_{S|UYZ}\left(s|u,y,z\right)\;d\left(s,\phi \left(w,y,z\right)\right)\\ \; & \geq \sum_{u,y,z}Q_{UYZ}\left(u,y,z\right)\;\sum_{w,s}P_{W|U}\left(w|u\right)\;Q_{S|UYZ}\left(s|u,y,z\right)\;d\left(s,\phi^{*}\left(u,y,z\right)\right)\\ \; & \geq \sum_{u,y,z}Q_{UYZ}\left(u,y,z\right)\;\min_{\phi (u,y,z)}\;\sum_{w,s}P_{W|U}\left(w|u\right)\;Q_{S|UYZ}\left(s|u,y,z\right)\;d\left(s,\phi \left(u,y,z\right)\right)\\ \; & =\sum_{u,y,z}Q_{UYZ}\left(u,y,z\right)\;\min_{\phi (u,y,z)}\;\sum_{s}Q_{S|UYZ}\left(s|u,y,z\right)\;d\left(s,\phi \left(u,y,z\right)\right)\\ \; & =\min_{\phi (\cdot ,\cdot ,\cdot )}E_{Q}\left[d\left(S,\phi (U,Y,Z)\right)\right]:=\Delta_{min}, & \; \end{array}$$

where in (53), $\phi^{*}\left(u,y,z\right)$ is chosen such that

$$\begin{array}{c} \phi^{*}\left(u,y,z\right):=\underset{\phi (w,y,z),w\in W}{arg min}\sum_{s}Q_{S|UYZ}\left(s|u,y,z\right)\;d\left(s,\phi \left(w,y,z\right)\right). \end{array}$$

*Converse:* Let $W=(W_{T},T)$ denote the auxiliary r.v. defined in the converse of Proposition 1. Inequalities (50) and (51) follow similarly as obtained in Proposition 1. We prove (52). Defining ${\tilde{\phi }}_{n}\left(M,Y^{n},Z^{n},S^{i-1},i\right):={\bar{\phi }}_{i,n}\left(M,Y^{n},Z^{n},S^{i-1}\right)$, we have

(54) $$\begin{array}{ccc} \min_{g_{i,n}^{\left(r\right)}}E\left[d\left(S^{n},{\hat{S}}^{n}\right)|H=0\right] & =\min_{{\left\{{\tilde{\phi }}_{n}\left(m,y^{n},z^{n},s^{i-1},i\right)\right\}}_{i=1}^{n}}E\left[\sum_{i=1}^{n}d\left(S_{i},{\tilde{\phi }}_{n}\left(M,Y^{n},Z^{n},S^{i-1},i\right)\right)|H=0\right]\\ \; & =\min_{{\left\{{\tilde{\phi }}_{n}\left(\cdot ,\cdot ,\cdot ,\cdot ,\cdot \right)\right\}}_{i=1}^{n}}E\left[\sum_{i=1}^{n}d\left(S_{i},{\tilde{\phi }}_{n}\left(W_{i},Z_{i},Y_{i},Y_{i+1}^{n},i\right)\right)|H=0\right]\\ \; & \leq \min_{\left\{\phi \left(w_{i},z_{i},y_{i},i\right)\right\}}E\left[\sum_{i=1}^{n}d\left(S_{i},\phi \left(W_{i},Z_{i},Y_{i},i\right)\right)|H=0\right]\\ \; & =n\min_{\left\{\phi (\cdot ,\cdot ,\cdot ,\cdot )\right\}}E\left[E\left[d\left(S_{T},\phi \left(W_{T},Z_{T},Y_{T},T\right)\right)|T\right]\;|H=0\right]\\ \; & =n\min_{\left\{\phi (\cdot ,\cdot ,\cdot ,\cdot )\right\}}E\left[d\left(S_{T},\phi \left(W_{T},Z_{T},Y_{T},T\right)\right)|H=0\right]\\ \; & =n\min_{\left\{\phi (w,z,y)\right\}}E\left[d\left(S,\phi (W,Z,Y)\right)|H=0\right], & \; \end{array}$$

where (54) is due to (A1) (in Appendix B). Hence, any $\Delta_{0}$ satisfying (6) satisfies

$$\begin{array}{cc} \Delta_{0} & \leq \min_{\left\{\phi (w,z,y)\right\}}E_{P}\left[d\left(S,\phi (W,Z,Y)\right)\right]. \end{array}$$

This completes the proof of the converse and the theorem. ☐

A more general version of Propositions 1 and 2 is claimed in [66] as Theorems 7 and 8, respectively, in which a privacy constraint under the alternate hypothesis is also imposed. However, we have identified a mistake in the converse proof; and hence, a single-letter characterization for this general problem remains open.

To complete the single-letter characterization in Propositions 1 and 2, we bound the alphabet size of the auxiliary r.v. *W* in the following lemma, whose proof is given in Appendix E.

**Lemma** **5.** *In Propositions 1 and 2, it suffices to consider auxiliary r.v.’s W such that |W|≤|U|+2.*

The proof of Lemma 5 uses standard arguments based on the Fenchel–Eggleston–Carathéodory’s theorem and is given in Appendix E.

**Remark** **1.** *When $Q_{S|UYZ}=Q_{S|YZ}$, a tight single-letter characterization of $R_{e}$ and $R_{d}$ exists even if the privacy constraint is active under the alternate hypothesis. This is due to the fact that given $Y^{n}$ and $Z^{n}$, M is independent of $S^{n}$ under the alternate hypothesis. In this case, $\left(R,\kappa ,\Lambda_{0},\Lambda_{1}\right)\in R_{e}$ if and only if there exists an auxiliary r.v. W, such that (Z,Y,S)−U−W, and*

(55) $$\begin{array}{cc} \kappa & \leq I_{P}\left(W;Y|Z\right), \end{array}$$

(56) $$\begin{array}{cc} R & \geq I_{P}\left(W;U|Z\right), \end{array}$$

(57) $$\begin{array}{cc} \Lambda_{0} & \leq H_{P}\left(S|W,Z,Y\right), \end{array}$$

(58) $$\begin{array}{cc} \Lambda_{1} & \leq H_{Q}\left(S|Z,Y\right), \end{array}$$

*for some $P_{SUYZW}$ as in Proposition 1. Similarly, we have that $\left(R,\kappa ,\Delta_{0},\Delta_{1}\right)\in R_{d}$ if and only if there exist an auxiliary r.v. W and a deterministic function $\phi :W\times Y\times Z\to \hat{S}$ such that (55) and (56),*

(59) $$\begin{array}{cc} \Delta_{0} & \leq \min_{\phi (\cdot ,\cdot ,\cdot )}E_{P}\left[d\left(S,\phi (W,Y,Z)\right)\right], \end{array}$$

(60) $$\begin{array}{cc} \Delta_{1} & \leq \min_{\phi (\cdot ,\cdot ,\cdot )}E_{Q}\left[d\left(S,\phi (Y,Z)\right)\right], \end{array}$$

*are satisfied for some $P_{SUYZW}$ as in Proposition 1.*

The computation of the trade-off given in Proposition 1 is challenging despite the cardinality bound on the auxiliary r.v. *W* provided by Lemma 5, as closed form solutions do not exist in general. To see this, note that the inequality constraints defining $R_{e}$ are not convex in general, and hence even computing specific points in the trade-off could be a hard problem. This is evident from the fact that in the absence of the privacy constraint in Proposition 1, i.e., (30), computing the maximum error exponent for a given rate constraint is equivalent to the information bottleneck problem [67], which is known to be a hard non-convex optimization problem. Also, the complexity of brute force search is exponential in |U|, and hence intractable for large values of |U|. Below we provide an example which can be solved in closed form and hence computed easily.

**Example** **1.** *Let V=U=S={0,1}, V=Y, Z= constant, V−S−U, $P_{U}\left(0\right)=Q_{U}\left(0\right)=0.5$, $P_{S|U}\left(0|0\right)=P_{S|U}\left(1|1\right)=Q_{S|U}\left(0|0\right)=Q_{S|U}\left(1|1\right)=1-q$, $P_{V|S}\left(0|0\right)=P_{V|S}\left(1|1\right)=1-p$ and $Q_{V|S}\left(0|0\right)=Q_{V|S}\left(1|1\right)=0.5$. Then, $\left(R,\kappa ,\Lambda_{0},0\right)\in R_{e}$ if there exists r∈[0,0.5] such that*

(61) $$\begin{array}{cc} R & \geq 1-h_{b}\left(r\right), \end{array}$$

(62) $$\begin{array}{cc} \kappa & \leq 1-h_{b}\left(\left(r*q\right)*p\right), \end{array}$$

(63) $$\begin{array}{cc} \Lambda_{0} & \leq h_{b}\left(p\right)+h_{b}\left(q*r\right)-h_{b}\left(p*\left(q*r\right)\right), \end{array}$$

where for a,b∈R, a*b:=(1−a)·b+(1−b)·a, and $h_{b}:\left[0,1\right]\mapsto \left[0,1\right]$ is the binary entropy function given by

$$\begin{array}{c} h_{b}\left(t\right)=-\left(1-t\right)\log\left(1-t\right)-t\log\left(t\right). \end{array}$$

*The above characterization (Numerical computation shows that the characterization given in (61)–(63) is exact even when q∈(0,1).) is exact for q=0, i.e., $\left(R,\kappa ,\Lambda_{0},0\right)\in R_{e}$ only if there exists r∈[0,0.5] such that (61)–(63) are satisfied.*

**Proof.**  Taking W={0,1}, and $P_{W|U}\left(0|0\right)=P_{W|U}\left(1|1\right)=1-r$, the constraints defining the trade-off given in Proposition 1 simplifies to

$$\begin{array}{ccc} I_{P}\left(U;W\right) & =1-h_{b}\left(r\right),\\ I_{P}\left(V;W\right) & =1-h_{b}\left(\left(r*q\right)*p\right),\\ H_{P}\left(S|V,W\right) & =H_{P}\left(S|W\right)-I_{P}\left(S;V|W\right)\\ \; & =H_{P}\left(S|W\right)+H_{P}\left(V|S\right)-H_{P}\left(V|W\right)\\ \; & =h_{b}\left(r*q\right)+h_{b}\left(p\right)-h_{b}\left(p*\left(q*r\right)\right). & \; \end{array}$$

On the other hand, if q=0, note that S=U. Hence, the same constraints can be bounded as follows:

$$\begin{array}{cc} I_{P}\left(U;W\right) & =1-H_{P}\left(U|W\right), \end{array}$$

(64) $$\begin{array}{cc} I_{P}\left(V;W\right) & =1-H_{P}\left(V|W\right)\leq 1-h_{b}\left(h_{b}^{-1}\left(H\left(U|W\right)\right)*p\right),\\ H_{P}\left(U|V,W\right) & =H_{P}\left(U|W\right)+H_{P}\left(V|U\right)-H_{P}\left(V|W\right) \end{array}$$

(65) $$\begin{array}{ccc} \; & \leq h_{b}\left(p\right)+H_{P}\left(U|W\right)-h_{b}\left(h_{b}^{-1}\left(H_{P}\left(U|W\right)\right)*p\right), & \; \end{array}$$

where $h_{b}^{-1}:\left[0,1\right]\mapsto \left[0,0.5\right]$ is the inverse of the binary entropy function. Here, the inequality in (64) and (65) follows by an application of Mrs Gerber’s lemma [68], since $V=U⊕N_{p}$ under the null hypothesis and $N_{p}∼Ber\left(p\right)$ is independent of *U* and *W*. Also, $\Lambda_{min}=0$ since S=U. Noting that $H_{P}\left(U|W\right)\in \left[0,1\right]$, and defining $r:=h_{b}^{-1}\left(H_{P}\left(U|W\right)\right)\in \left[0,0.5\right]$, the result follows. ☐

**Figure 2:**
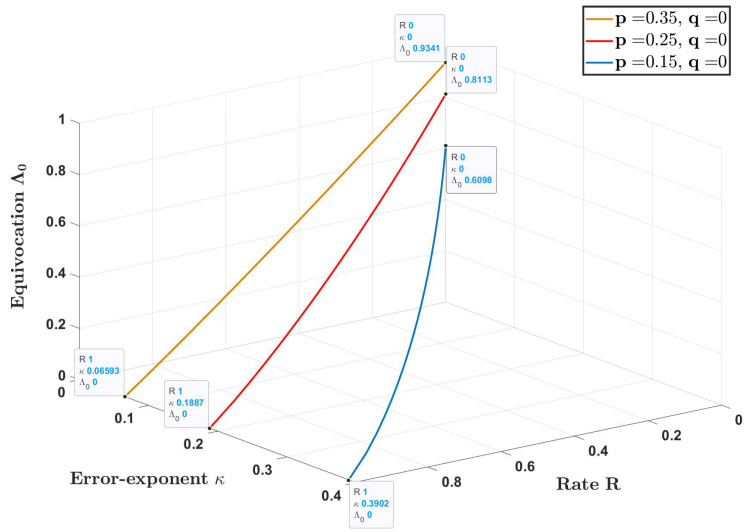
$\left(R,\kappa ,\Lambda_{0}\right)$ trade-off at the boundary of $R_{e}$ in Example 1 (Axes units are in bits)

**Figure 3:**
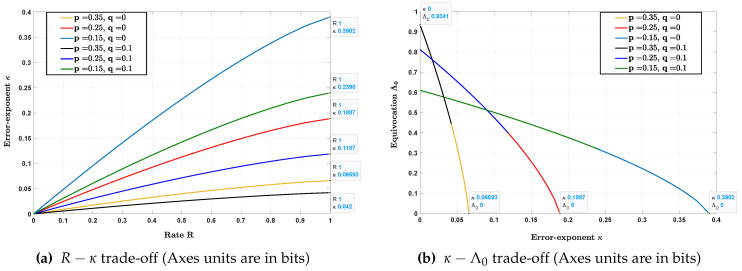
Projections of Figure 2 in the R−κ plane and $\kappa -\Lambda_{0}$ plane

Figure 2 depicts the curve $\left(1-h_{b}\left(r\right),1-h_{b}\left(p*\left(q*r\right)\right),h_{b}\left(p\right)+h_{b}\left(r*q\right)-h_{b}\left(p*\left(r*q\right)\right)\right)$ for q=0 and p∈{0.15,0.25,0.35}, as *r* is varied in the range [0,0.5]. The projection of this curve on the R−κ and $\kappa -\Lambda_{0}$ plane is shown in Figure 3a,b, respectively, for q∈{0,0.1} and the same values of *p*. As expected, the error exponent κ increases with rate *R* while the equivocation $\Lambda_{0}$ decreases with κ at the boundary of $R_{e}$.

Proposition 1 (resp. Proposition 2) provide a characterization of $R_{e}$ (resp. $R_{d}$) under the condition of vanishing type I error probability constraint. Consequently, the converse part of these results are known as *weak converse* results in the context of HT. In the next subsection, we establish the optimal error exponent-privacy trade-off for the special case of zero-rate compression. This trade-off is independent of the type I error probability constraint ϵ∈(0,1), and hence known as a *strong converse* result.

### 4.2. Zero-Rate Compression

Assume the following zero-rate constraint on the communication between the observer and the detector,

(66) $$\begin{array}{c} \lim_{n\to \infty }\frac{\log(|M|)}{n}=0. \end{array}$$

Please note that (66) does not imply that |M|=0, i.e., nothing can be transmitted, but that the message set cardinality can grow at most sub-exponentially in *n*. Such a scenario is motivated practically by low power or low bandwidth constrained applications in which communication is costly. Propositions 3 and 4 stated below provide an optimal single-letter characterization of $R_{d}\left(\epsilon \right)$ and $R_{e}\left(\epsilon \right)$ in this case. While the coding schemes in the achievability part of these results are inspired from that in [6], the analysis of privacy achieved at the detector is new. Lemma 4 serves as a crucial tool for this purpose. We next state the results. Let

(67a) $$\begin{array}{cc} \Delta_{0}^{max} & :=\min_{\phi^{\prime }\left(\cdot \right)}E_{P}\left[d\left(S,\phi^{\prime }\left(V\right)\right)\right], \end{array}$$

(67b) $$\begin{array}{cc} \mathrm{and}\;\Delta_{1}^{max} & :=\min_{\phi^{\prime }\left(\cdot \right)}E_{Q}\left[d\left(S,\phi^{\prime }\left(V\right)\right)\right]. \end{array}$$

**Proposition** **3.** *For ϵ∈(0,1), $\left(0,\kappa ,\Delta_{0},\Delta_{1}\right)\in R_{d}\left(\epsilon \right)$ if and only if it satisfies,*

(68) $$\begin{array}{cc} \kappa & \leq \min_{P_{\tilde{U}\tilde{V}}\in L^{\prime }\left(P_{U},P_{V}\right)}D(P_{\tilde{U}\tilde{V}}\left|\right|Q_{UV}), \end{array}$$

(69) $$\begin{array}{cc} \Delta_{0} & \leq \Delta_{0}^{max}, \end{array}$$

(70) $$\begin{array}{cc} \Delta_{1} & \leq \Delta_{1}^{max}, \end{array}$$

*where $\phi^{\prime }:V\to \hat{S}$ is a deterministic function and*

$$\begin{array}{cc} L^{\prime }\left(P_{U},P_{V}\right) & =\{P_{\tilde{U}\tilde{V}}\in P\left(U\times V\right):P_{\tilde{U}}=P_{U},\;P_{\tilde{V}}=P_{V}\}. \end{array}$$

**Proof.**  First, we prove that $\left(0,\kappa ,\Delta_{0},\Delta_{1}\right)$ satisfying (68)–(70) is achievable. While the encoding and decoding scheme is the same as that in [6], we mention it for the sake of completeness.

**Encoding:** The observer sends the message M=1 if $U^{n}\in T_{{\left[P_{U}\right]}_{\delta }}^{n}$, δ>0, and M=0 otherwise.

**Decoding:** The detector declares $\hat{H}=0$ if M=1 and $V^{n}\in T_{{\left[P_{V}\right]}_{\delta }}^{n}$, δ>0. Otherwise, $\hat{H}=1$ is declared.

We analyze the type I and type II error probabilities for the above scheme. Please note that for any δ>0, the weak law of large numbers implies that

$$\begin{array}{c} P\left(U^{n}\in T_{{\left[P_{U}\right]}_{\delta }}^{n}\cap V^{n}\in T_{{\left[P_{V}\right]}_{\delta }}^{n})|H=0\right)=P\left(M=1\cap V^{n}\in T_{{\left[P_{V}\right]}_{\delta }}^{n})|H=0\right)\overset{\left(n\right)}{\to }1. \end{array}$$

Hence, the type I error probability tends to zero, asymptotically. The type II error probability can be written as follows:

$$\begin{array}{ccc} \beta_{n}\left(f_{n},g_{n}\right) & =P(U^{n}\in T_{{\left[P_{U}\right]}_{\delta }}^{n}\cap V^{n}\in T_{{\left[P_{V}\right]}_{\delta }}^{n})|H=1)\\ \; & =\sum_{\begin{array}{c} u^{n}\in T_{{\left[P_{U}\right]}_{\delta }}^{n},\\ v^{n}\in T_{{\left[P_{V}\right]}_{\delta }} \end{array}}Q_{U^{n}V^{n}}\left(u^{n},v^{n}\right)\leq {\left(n+1\right)}^{\left|U||V\right|}e^{-n(\kappa^{*}-O\left(\delta \right))}=e^{-n\left(\kappa^{*}-\frac{\left|U||V|\log(n+1\right)}{n}-O\left(\delta \right)\right)}, & \; \end{array}$$

where

$$\begin{array}{c} \kappa^{*}=\min_{P_{\tilde{U}\tilde{V}}\in L^{\prime }\left(P_{U},P_{V}\right)}D(P_{\tilde{U}\tilde{V}}\left|\right|Q_{UV}). \end{array}$$

Next, we lower bound the average distortion for $S^{n}$ achieved by this scheme at the detector. Defining

(71) $$\begin{array}{cc} \Pi (U^{n},\delta ,P_{U}) & :=1\left(U^{n}\notin T_{{\left[P_{U}\right]}_{\delta }}^{n}\right), \end{array}$$

(72) $$\begin{array}{cc} \rho_{n}^{\left(0\right)}\left(\delta \right) & :=∥P_{S^{n}V^{n}}\left(\cdot \right)-P_{S^{n}V^{n}|\Pi \left(U^{n},\delta ,P_{U}\right)}\left(\cdot |0\right)∥,, \end{array}$$

(73) $$\begin{array}{cc} \rho_{n}^{\left(1\right)}\left(\delta \right) & :=∥Q_{S^{n}V^{n}}\left(\cdot \right)-Q_{S^{n}V^{n}|\Pi \left(U^{n},\delta ,P_{U}\right)}\left(\cdot |1\right)∥,\\ \phi_{n}^{\prime }\left(v^{n}\right) & :=(\phi^{\prime }\left(v_{1}\right),\cdots ,\phi^{\prime }\left(v_{n}\right)), \end{array}$$

we can write

(74) $$\begin{array}{cc} \; & \left|\min_{{\left\{{\bar{\phi }}_{i}\left(m,v^{n},s^{i-1}\right)\right\}}_{i=1}^{n}}E\left[d\left(S^{n},{\hat{S}}^{n}\right)|H=0\right]-n\min_{\phi^{\prime }\left(v\right)}E_{P}\left[d\left(S,\phi^{\prime }\left(V\right)\right)\right]\right|\\ \; & =|\min_{{\left\{{\bar{\phi }}_{i}\left(m,v^{n},s^{i-1}\right)\right\}}_{i=1}^{n}}E\left[d\left(S^{n},{\hat{S}}^{n}\right)|H=0\right]-\min_{\phi_{n}^{\prime }\left(v^{n}\right)}E\left[d\left(S^{n},\phi_{n}^{\prime }\left(V^{n}\right)\right)|H=0\right]|\\ \; & \leq |\min_{{\left\{{\bar{\phi }}_{i}\left(m,v^{n},s^{i-1}\right)\right\}}_{i=1}^{n}}E\left[d\left(S^{n},{\hat{S}}^{n}\right)|H=0\right]-P\left(M=1|H=0\right)\min_{\phi_{n}^{\prime }\left(v^{n}\right)}E\left[d\left(S^{n},\phi_{n}^{\prime }\left(V^{n}\right)\right)|M=1,H=0\right]|\\ \; & \;+P\left(M=0|H=0\right)\min_{\phi_{n}^{\prime }\left(v^{n}\right)}E\left[d\left(S^{n},\phi_{n}^{\prime }\left(V^{n}\right)\right)|M=0,H=0\right]\\ \; & \leq |\min_{{\left\{{\bar{\phi }}_{i}\left(m,v^{n},s^{i-1}\right)\right\}}_{i=1}^{n}}E\left[d\left(S^{n},{\hat{S}}^{n}\right)|H=0\right]-\min_{\phi_{n}^{\prime }\left(v^{n}\right)}E\left[d\left(S^{n},\phi_{n}^{\prime }\left(V^{n}\right)\right)|M=1,H=0\right]|\\ \; & \;+P\left(M=0|H=0\right)[\min_{\phi_{n}^{\prime }\left(v^{n}\right)}E\left[d\left(S^{n},\phi_{n}^{\prime }\left(V^{n}\right)\right)|M=1,H=0\right]\\ \; & \;\;+\min_{\phi_{n}^{\prime }\left(v^{n}\right)}E\left[d\left(S^{n},\phi_{n}^{\prime }\left(V^{n}\right)\right)|M=0,H=0\right]]\\ \; & =|\min_{{\left\{{\bar{\phi }}_{i}\left(m,v^{n},s^{i-1}\right)\right\}}_{i=1}^{n}}E\left[d\left(S^{n},{\hat{S}}^{n}\right)|H=0\right]-\min_{\phi_{n}^{\prime }\left(v^{n}\right)}E\left[d\left(S^{n},\phi_{n}^{\prime }\left(V^{n}\right)\right)|\Pi \left(U^{n},\delta ,P_{U}\right)=0,H=0\right]|\\ \; & \;+P\left(\Pi (U^{n},\delta ,P_{U})=1|H=0\right)[\min_{\phi_{n}^{\prime }\left(v^{n}\right)}E\left[d\left(S^{n},\phi_{n}^{\prime }\left(V^{n}\right)\right)|M=1,H=0\right]\\ \; & \;\;\;\;\;\;\;\;+\min_{\phi_{n}^{\prime }\left(v^{n}\right)}E\left[d\left(S^{n},\phi_{n}^{\prime }\left(V^{n}\right)\right)|M=0,H=0\right]] \end{array}$$

(75) $$\begin{array}{cc} \; & \leq nD_{m}\rho_{n}^{\left(0\right)}\left(\delta \right)+2\;e^{-n\Omega (\delta )}nD_{m} \end{array}$$

(76) $$\begin{array}{ccc} \; & \overset{\left(n\right)}{\to }0, & \; \end{array}$$

where (74) is since $\Pi (U^{n},\delta ,P_{U})=1-M$ with probability one by the encoding scheme; (75) follows from

(77) $$\begin{array}{c} P\left(\Pi (U^{n},\delta ,P_{U})=1|H=0\right)=P\left(U^{n}\notin T_{{\left[P_{U}\right]}_{\delta }}^{n}|H=0\right)\leq e^{-n\Omega (\delta )} \end{array}$$

and ([43], Property 2(b)); and, (76) is due to (17). Similarly, it can be shown using (16) that if $Q_{U}=P_{U}$, then

(78) $$\begin{array}{c} |\min_{{\left\{{\bar{\phi }}_{i,n}\left(m,v^{n},s^{i-1}\right)\right\}}_{i=1}^{n}}E\left[d\left(S^{n},{\hat{S}}^{n}\right)|H=1\right]-n\min_{\phi^{\prime }\left(v\right)}E_{Q}\left[d\left(S,\phi^{\prime }\left(V\right)\right)\right]|\overset{\left(n\right)}{\to }0. \end{array}$$

On the other hand, if $Q_{U}\neq P_{U}$ and δ is small enough, we have

(79) $$\begin{array}{c} P\left(M=0|H=1\right)=P\left(\Pi (U^{n},\delta ,P_{U})=1|H=1\right)\geq 1-e^{-n(D(P_{U}\left|\right|Q_{U})-O\left(\delta \right))}\overset{\left(n\right)}{\to }1. \end{array}$$

Hence, we can write for δ small enough,

(80) $$\begin{array}{cc} \; & \left|\min_{{\left\{{\bar{\phi }}_{i}\left(m,v^{n},s^{i-1}\right)\right\}}_{i=1}^{n}}E\left[d\left(S^{n},{\hat{S}}^{n}\right)|H=1\right]-n\min_{\phi^{\prime }\left(v\right)}E_{Q}\left[d\left(S,\phi^{\prime }\left(V\right)\right)\right]\right|\\ \; & =|\min_{{\left\{{\bar{\phi }}_{i}\left(m,v^{n},s^{i-1}\right)\right\}}_{i=1}^{n}}E\left[d\left(S^{n},{\hat{S}}^{n}\right)|H=1\right]-\min_{\phi_{n}^{\prime }\left(v^{n}\right)}E\left[d\left(S^{n},\phi_{n}^{\prime }\left(V^{n}\right)\right)|H=1\right]|\\ \; & \leq |\min_{{\left\{{\bar{\phi }}_{i}\left(m,v^{n},s^{i-1}\right)\right\}}_{i=1}^{n}}E\left[d\left(S^{n},{\hat{S}}^{n}\right)|H=1\right]-P\left(M=0|H=0\right)\min_{\phi_{n}^{\prime }\left(v^{n}\right)}E\left[d\left(S^{n},\phi_{n}^{\prime }\left(V^{n}\right)\right)|M=0,H=1\right]|\\ \; & \;+P\left(M=1|H=1\right)\min_{\phi_{n}^{\prime }\left(v^{n}\right)}E\left[d\left(S^{n},\phi_{n}^{\prime }\left(V^{n}\right)\right)|M=1,H=1\right]\\ \; & \leq |\min_{{\left\{{\bar{\phi }}_{i}\left(m,v^{n},s^{i-1}\right)\right\}}_{i=1}^{n}}E\left[d\left(S^{n},{\hat{S}}^{n}\right)|H=1\right]-\min_{\phi_{n}^{\prime }\left(v^{n}\right)}E\left[d\left(S^{n},\phi_{n}^{\prime }\left(V^{n}\right)\right)|M=0,H=1\right]|\\ \; & \;+P\left(M=1|H=1\right)[\min_{\phi_{n}^{\prime }\left(v^{n}\right)}E\left[d\left(S^{n},\phi_{n}^{\prime }\left(V^{n}\right)\right)|M=1,H=1\right]\\ \; & \;\;\;\;\;\;\;\;+\min_{\phi_{n}^{\prime }\left(v^{n}\right)}E\left[d\left(S^{n},\phi_{n}^{\prime }\left(V^{n}\right)\right)|M=0,H=1\right]]\\ \; & =|\min_{{\left\{{\bar{\phi }}_{i}\left(m,v^{n},s^{i-1}\right)\right\}}_{i=1}^{n}}E\left[d\left(S^{n},{\hat{S}}^{n}\right)|H=1\right]-\min_{\phi_{n}^{\prime }\left(v^{n}\right)}E\left[d\left(S^{n},\phi_{n}^{\prime }\left(V^{n}\right)\right)|\Pi \left(U^{n},\delta ,P_{U}\right)=1,H=1\right]|\\ \; & \;+P\left(\Pi (U^{n},\delta ,P_{U})=0|H=1\right)[\min_{\phi_{n}^{\prime }\left(v^{n}\right)}E\left[d\left(S^{n},\phi_{n}^{\prime }\left(V^{n}\right)\right)|M=1,H=1\right]\\ \; & \;\;\;\;\;\;\;\;+\min_{\phi_{n}^{\prime }\left(v^{n}\right)}E\left[d\left(S^{n},\phi_{n}^{\prime }\left(V^{n}\right)\right)|M=0,H=1\right]] \end{array}$$

(81) $$\begin{array}{cc} \; & \leq nD_{m}\rho_{n}^{\left(1\right)}\left(\delta \right)+2\;e^{-n(D(P_{U}\left|\right|Q_{U})-O\left(\delta \right))}nD_{m} \end{array}$$

(82) $$\begin{array}{ccc} \; & \overset{\left(n\right)}{\to }0, & \; \end{array}$$

where (80) is since $\Pi (U^{n},\delta ,P_{U})=1-M$ with probability one; (81) is due to (79) and ([43], Property 2(b)); and, (82) follows from (15). This completes the proof of the achievability.

We next prove the converse. Please note that by the strong converse result in [8], the right hand side (R.H.S) of (68) is an upper bound on the achievable error exponent for all ϵ∈(0,1) even without a privacy constraint (hence, also with a privacy constraint). Also,

(83) $$\begin{array}{ccc} \min_{g_{i,n}^{\left(r\right)}}E\left[d\left(S^{n},{\hat{S}}^{n}\right)|H=0\right] & \leq \min_{{\left\{\phi^{\prime }\left(v_{i}\right)\right\}}_{i=1}^{n}}\sum_{i=1}^{n}E_{P_{S_{i}V_{i}}}\left[d\left(S_{i},\phi^{\prime }\left(V_{i}\right)\right)\right]\\ \; & =n\min_{\left\{\phi^{\prime }\left(v\right)\right\}}E_{P}\left[d(S,\phi^{\prime }\left(V\right))\right]. & \; \end{array}$$

Here, (83) follows from the fact that the detector can always reconstruct ${\hat{S}}_{i}$ as a function of $V_{i}$ for i∈[n]. Similarly,

$$\begin{array}{cc} \; & \min_{g_{i,n}^{\left(r\right)}}E\left[d\left(S^{n},{\hat{S}}^{n}\right)|H=1\right]\leq n\min_{\left\{\phi^{\prime }\left(v\right)\right\}}E_{Q}\left[d(S,\phi^{\prime }\left(V\right))\right]. \end{array}$$

Hence, any achievable $\Lambda_{0}$ and $\Lambda_{1}$ must satisfy (69) and (70), respectively. This completes the proof. ☐

The following Proposition is the analogous result to Proposition 3 when the privacy measure is equivocation.

**Proposition** **4.** *For ϵ∈(0,1), $\left(0,\kappa ,\Lambda_{0},\Lambda_{1}\right)\in R_{e}\left(\epsilon \right)$ if and only if it satisfies (68) and*

(84) $$\begin{array}{cc} \Lambda_{0} & \leq H_{P}\left(S|V\right), \end{array}$$

(85) $$\begin{array}{cc} \Lambda_{1} & \leq H_{Q}\left(S|V\right). \end{array}$$

**Proof.**  For proving the achievability part, the encoding and decoding scheme is the same as in Proposition 3. Hence, the analysis of the error exponent given in Proposition 3 holds. To lower bound the equivocation of $S^{n}$ at the detector, defining $\Pi (U^{n},\delta ,P_{U})$, $\rho_{n}^{\left(0\right)}\left(\delta \right)$ and $\rho_{n}^{\left(1\right)}\left(\delta \right)$ as in (71)–(73), we can write

(86) $$\begin{array}{cc} \; & \left|nH_{P}\left(S|V\right)-H\left(S^{n}|M,V^{n},H=0\right)\right|\\ \; & =|H\left(S^{n}|V^{n},H=0\right)-H\left(S^{n}|M,V^{n},H=0\right)|\\ \; & \leq |H\left(S^{n},V^{n}|H=0\right)-H\left(S^{n},V^{n}|M,H=0\right)|\\ \; & \leq |H\left(S^{n},V^{n}|H=0\right)-P\left(M=1|H=0\right)\;H\left(S^{n},V^{n}|M=1,H=0\right)|\\ \; & \;\;+P\left(M=0|H=0\right)H\left(S^{n},V^{n}|M=0,H=0\right)\\ \; & \leq |H\left(S^{n},V^{n}|H=0\right)-H\left(S^{n},V^{n}|M=1,H=0\right)|\\ \; & \;\;+P\left(M=0|H=0\right)\left(H\left(S^{n},V^{n}|M=1,H=0\right)+H\left(S^{n},V^{n}|M=0,H=0\right)\right)\\ \; & \leq |H\left(S^{n},V^{n}|H=0\right)-H\left(S^{n},V^{n}|\Pi \left(U^{n},\delta ,P_{U}\right)=0,H=0\right)|\\ \; & \;\;+P\left(\Pi (U^{n},\delta ,P_{U})=1|H=0\right)\left(H\left(S^{n},V^{n}|M=1,H=0\right)+H\left(S^{n},V^{n}|M=0,H=0\right)\right)\\ \; & \overset{\left(n\right)}{\leq }-2\rho_{n}^{\left(0\right)}\left(\delta \right)\log\left(\frac{\rho_{n}^{\left(0\right)}\left(\delta \right)}{{\left|S\right|}^{n}{\left|V\right|}^{n}}\right)+2\;e^{-n\Omega (\delta )}\log\left({\left|S\right|}^{n}{\left|V\right|}^{n}\right) \end{array}$$

(87) $$\begin{array}{ccc} \; & \overset{\left(n\right)}{\to }0, & \; \end{array}$$

where (86) follows due to Lemma 3, ([60], Lemma 2.12) and the fact that entropy of a r.v. is bounded by the logarithm of cardinality of its support; and, (87) follows from (17) in Lemma 4 since δ>0. In a similar way, it can be shown using (16) that if $Q_{U}=P_{U}$, then

(88) $$\begin{array}{cc} \; & |H\left(S^{n}|V^{n},H=1\right)-H\left(S^{n}|M,V^{n},H=1\right)|\overset{\left(n\right)}{\to }0. \end{array}$$

On the other hand, if $Q_{U}\neq P_{U}$ and δ is small enough, we can write

(89) $$\begin{array}{ccc} \; & |nH_{Q}\left(S|V\right)-H\left(S^{n}|M,V^{n},H=1\right)|\\ \; & =|H\left(S^{n}|V^{n},H=1\right)-H\left(S^{n}|M,V^{n},H=1\right)|\\ \; & \leq |H\left(S^{n},V^{n}|H=1\right)-H\left(S^{n},V^{n}|M,H=1\right)|\\ \; & \leq |H\left(S^{n},V^{n}|H=1\right)-H\left(S^{n},V^{n}|M=0,H=1\right)|\\ \; & \;+P\left(\Pi (U^{n},\delta ,P_{U})=0|H=1\right)\left(H\left(S^{n},V^{n}|M=0,H=1\right)+H\left(S^{n},V^{n}|M=1,H=1\right)\right)\\ \; & \leq -2\rho_{n}^{\left(1\right)}\left(\delta \right)\log\left(\frac{\rho_{n}^{\left(1\right)}\left(\delta \right)}{{\left|S\right|}^{n}{\left|V\right|}^{n}}\right)+2\;e^{-n(D(P_{U}\left|\right|Q_{U})-O\left(\delta \right))}\log\left({\left|S\right|}^{n}{\left|V\right|}^{n}\right), & \; \end{array}$$

where (89) follows from Lemma 3 and (79). It follows from (15) in Lemma 4 that for δ>0 sufficiently small, $\rho_{n}^{\left(1\right)}\left(\delta \right)\leq e^{-n\bar{\delta }}$ for some $\bar{\delta }>0$, thus implying that the R.H.S. of (89) tends to zero. This completes the proof of achievability.

The converse follows from the results in [6,8] that the R.H.S of (68) is the optimal error exponent achievable for all values of ϵ∈(0,1) even when there is no privacy constraint, and the following inequality

(90) $$\begin{array}{c} H\left(S^{n}|M,V^{n},H=j\right)\leq H\left(S^{n}|V^{n},H=j\right),\;j=0,1. \end{array}$$

This concludes the proof of the Proposition. ☐

In Section 2.2, we mentioned that it is possible to achieve a positive error exponent with perfect privacy in our model. Here, we provide an example of TAI with an equivocation privacy constraint under both hypothesis, and show that perfect privacy is possible. Recall that TAI is a special case of TACI, in which Z= constant, and hence, the null and alternate hypothesis are given by

$$\begin{array}{cc} \; & H_{0}:\left(U^{n},Y^{n}\right)∼\prod_{i=1}^{n}P_{UY},\\ \mathrm{and}\; & H_{1}:\left(U^{n},Y^{n}\right)∼\prod_{i=1}^{n}P_{U}P_{Y}. \end{array}$$

**Example** **2.** *Let S=U={0,1,2,3}, Y={0,1},*

$$P_{SU}=0.125\cdot \left[\begin{array}{cccc} 1 & 1 & 0 & 0\\ 1 & 1 & 0 & 0\\ 0 & 0 & 1 & 1\\ 0 & 0 & 1 & 1 \end{array}\right],\;\;P_{Y|U}=\left[\begin{array}{cc} 1 & 0\\ 0 & 1\\ 1 & 0\\ 0 & 1 \end{array}\right],$$

*$P_{SUY}:=P_{SU}P_{Y|U}$ and $Q_{SUY}:=P_{SU}P_{Y}$, where $P_{Y}=\sum_{u\in U}P_{U}\left(u\right)P_{Y|U}\left(y|u\right)$. Then, we have $H_{Q}\left(S|Y\right)=H_{P}\left(S\right)=H_{P}\left(U\right)=2$ bits. Also, noting that under the null hypothesis, Y=Umod2, $H_{P}\left(S|Y\right)=2$ bits. It follows from the inner bound given by Equations (31)–(34), and, (37) and (38) that $\left(R,\kappa ,\Lambda_{0},\Lambda_{1}\right)\in R_{e}\left(\epsilon \right)$, ϵ∈(0,1) if*

$$\begin{array}{cc} R & \geq I_{P}\left(W;U\right),\\ \kappa & \leq I_{P}\left(W;Y\right),\\ \Lambda_{0} & \leq H_{P}\left(S|W,Y\right),\\ \Lambda_{1} & \leq H_{Q}\left(S|W,Y\right)=H_{Q}\left(S|W\right), \end{array}$$

*where $P_{SUYW}:=P_{SUY}P_{W|U}$ and $Q_{SUYW}:=Q_{SUY}P_{W|U}$ for some conditional distribution $P_{W|U}$. If we set W:=Umod2, then we have $I_{P}\left(U;W\right)=1$ bit, $I_{P}\left(Y;W\right)=H_{P}\left(Y\right)=1$ bit, $H_{P}\left(S|W,Y\right)=H_{P}\left(S|Y\right)=2$ bits, and $H_{Q}\left(S|W\right)=H_{P}\left(S|Y\right)=2$ bits. Thus, by revealing only W to the detector, it is possible to achieve a positive error exponent while ensuring maximum privacy under both the null and alternate hypothesis, i.e., the tuple $\left(1,1,2,2\right)\in R_{e}\left(\epsilon \right)$, ∀ϵ∈(0,1).*

## 5. A Counter-Example to the Strong Converse

Ahlswede and Csiszár obtained a strong converse result for the DHT problem without a privacy constraint in [5], where they showed that for any positive rate *R*, the optimal achievable error exponent is independent of the type I error probability constraint ϵ. Here, we explore whether a similar result holds in our model, in which an additional privacy constraint is imposed. We will show through a counter-example that this is not the case in general. The basic idea used in the counter-example is a “time-sharing” argument which is used to construct from a given coding scheme that achieves the optimal rate-error exponent-equivocation trade-off under a vanishing type I error probability constraint, a new coding scheme that satisfies the given type I error probability constraint $\epsilon^{*}$ and the same error exponent as before, yet achieves a higher equivocation for $S^{n}$ at the detector. This concept has been used previously in other contexts, e.g., in the characterization of the first-order maximal channel coding rate of additive white gaussian noise (AWGN) channel in the finite block-length regime [69], and subsequently in the characterization of the second order maximal coding rate in the same setting [70]. However, we will provide a self-contained proof of the counter-example by using Lemma 4 for this purpose.

Assume that the joint distribution $P_{SUV}$ is such that $H_{P}\left(S|U,V\right)<H_{P}\left(S|V\right)$. Proving the strong converse amounts to showing that any $\left(R,\kappa ,\Lambda_{0},\Lambda_{1}\right)\in R_{e}\left(\epsilon \right)$ for some ϵ∈(0,1) also belongs to $R_{e}$. Consider TAI problem with an equivocation privacy constraint, in which $R\geq H_{P}\left(U\right)$ and $\Lambda_{1}\leq \Lambda_{min}$. Then, from the optimal single-letter characterization of $R_{e}$ given in Proposition 1, it follows by taking W=U that $\left(H_{P}\left(U\right),I_{P}\left(V;U\right),H_{P}\left(S|V,U\right),\Lambda_{min}\right)\in R_{e}$. Please note that $I_{P}\left(V;U\right)$ is the maximum error exponent achievable for any type I error probability constraint ϵ∈(0,1), even when $U^{n}$ is observed directly at the detector. Thus, for vanishing type I error probability constraint ϵ→0 and $\kappa =I_{P}\left(V;U\right)$, the term $H_{P}\left(S|V,U\right)$ denotes the maximum achievable equivocation for $S^{n}$ under the null hypothesis. From the proof of Proposition 1, the coding scheme achieving this tuple is as follows:

1. Quantize $u^{n}$ to codewords in $B_{n}=\left\{u^{n}\left(j\right)\in T_{{\left[P_{U}\right]}_{\delta }}^{n},\;j\in \left[e^{n(H_{P}\left(U\right)+\eta )}\right]\right\}$ and send the index of quantization to the detector, i.e., if $u^{n}\in T_{{\left[P_{U}\right]}_{\delta }}^{n}$, send M=j, where *j* is the index of $u^{n}$ in $B_{n}$. Else, send M=0.
2. At the detector, if M=0, declare $\hat{H}=1$. Else, declare $\hat{H}=0$ if $\left(u^{n}\left(M\right),v^{n}\right)\in T_{{\left[P_{UV}\right]}_{\delta^{\prime }}}^{n}$ for some $\delta^{\prime }>\delta$, and $\hat{H}=1$ otherwise.

The type I error probability of the above scheme tends to zero asymptotically with *n*. Now, for a fixed $\epsilon^{*}>0$, consider a modification of this coding scheme as follows:

1. If $u^{n}\in T_{{\left[P_{U}\right]}_{\delta }}^{n}$, send M=j with probability $1-\epsilon^{*}$, where *j* is the index of $u^{n}$ in $B_{n}$, and with probability $\epsilon^{*}$, send M=0. If $u^{n}\notin T_{{\left[P_{U}\right]}_{\delta }}^{n}$, send M=0.
2. At the detector, if M=0, declare $\hat{H}=1$. Else, declare $\hat{H}=0$ if $u^{n}\left(M\right),v^{n})\in T_{{\left[P_{UV}\right]}_{\delta^{\prime }}}^{n}$ for some $\delta^{\prime }>\delta$, and $\hat{H}=1$ otherwise.

It is easy to see that for this modified coding scheme, the type I error probability is asymptotically equal to $\epsilon^{*}$, while the error exponent remains the same as I(V;U) since the probability of declaring $\hat{H}=0$ is decreased. Recalling that $\Pi \left(u^{n},\delta ,P_{U}\right):=1\left(u^{n}\notin T_{{\left[P_{U}\right]}_{\delta }}^{n}\right)$, we also have

(91) $$\begin{array}{cc} \; & \frac{1}{n}H\left(S^{n}|M,V^{n},H=0\right)\\ \; & =\left(1-\gamma_{n}\right)\left(1-\epsilon^{*}\right)\frac{1}{n}H\left(S^{n}|U^{n},V^{n},\Pi \left(U^{n},\delta ,P_{U}\right)=0,H=0\right)+\left(1-\gamma_{n}\right)\;\epsilon^{*}\\ \; & \;\frac{1}{n}H\left(S^{n}|M=0,V^{n},\Pi \left(U^{n},\delta ,P_{U}\right)=0,H=0\right)+\gamma_{n}\;\frac{1}{n}H\left(S^{n}|M=0,V^{n},\Pi \left(U^{n},\delta ,P_{U}\right)=1,H=0\right)\\ \; & \geq \left(1-\gamma_{n}\right)\left(1-\epsilon^{*}\right)\;\left(H_{P}\left(S|U,V\right)-\gamma_{n}^{\prime \prime }\right)+\left(1-\gamma_{n}\right)\;\epsilon^{*}\;\frac{1}{n}H\left(S^{n}|M=0,V^{n},\Pi \left(U^{n},\delta ,P_{U}\right)=0,H=0\right)\\ \; & \;+\gamma_{n}\;\frac{1}{n}H\left(S^{n}|M=0,V^{n},\Pi \left(U^{n},\delta ,P_{U}\right)=1,H=0\right)\\ \; & >\left(1-\gamma_{n}\right)\left(1-\epsilon^{*}\right)\;\left(H_{P}\left(S|U,V\right)-\gamma_{n}^{\prime \prime }\right)+\left(1-\gamma_{n}\right)\;\epsilon^{*}\;\left(H_{P}\left(S|U,V\right)-\frac{\gamma_{n}^{\prime }}{n}\right) \end{array}$$

(92) $$\begin{array}{cc} \; & \;+\gamma_{n}\;\frac{1}{n}H\left(S^{n}|M=0,V^{n},H=0,\Pi \left(U^{n},\delta ,P_{U}\right)=1\right) \end{array}$$

(93) $$\begin{array}{cc} \; & =\left(1-\gamma_{n}\right)\left(1-\epsilon^{*}\right)\;\left(H_{P}\left(S|U,V\right)-\gamma_{n}^{\prime \prime }\right)+\left(1-\gamma_{n}\right)\;\epsilon^{*}\left(H_{P}\left(S|U,V\right)-\frac{\gamma_{n}^{\prime }}{n}\right)+\gamma_{n}^{\prime \prime \prime } \end{array}$$

(94) $$\begin{array}{ccc} \; & =\left(1-\gamma_{n}\right)H_{P}\left(S|U,V\right)-{\bar{\gamma }}_{n}, & \; \end{array}$$

where ${\left\{\gamma_{n}^{\prime \prime }\right\}}_{n\in N}$ denotes some sequence of positive numbers such that $\gamma_{n}^{\prime \prime }\overset{\left(n\right)}{\to }0$, and

(95) $$\begin{array}{cc} \; & \gamma_{n}:=P\left(U^{n}\notin T_{{\left[P_{U}\right]}_{\delta }}^{n}|H=0\right)\leq e^{-n\Omega (\delta )}\overset{\left(n\right)}{\to }0,\\ \; & \gamma_{n}^{\prime }:=-2\rho_{n}^{*}\log\left(\frac{2\rho_{n}^{*}}{{\left|S\right|}^{n}}\right), \end{array}$$

(96) $$\begin{array}{cc} \; & \rho_{n}^{*}:=∥P_{S^{n}V^{n}|\Pi \left(U^{n},\delta ,P_{U}\right),M}\left(\cdot |0,0\right)-P_{S^{n}V^{n}}\left(\cdot \right)∥=∥P_{S^{n}V^{n}|\Pi \left(U^{n},\delta ,P_{U}\right)}\left(\cdot |0\right)-P_{S^{n}V^{n}}\left(\cdot \right)∥, \end{array}$$

(97) $$\begin{array}{cc} \; & \gamma_{n}^{\prime \prime \prime }:=\frac{\gamma_{n}}{n}H\left(S^{n}|M=0,V^{n},,H=0,\Pi \left(U^{n},\delta ,P_{U}\right)=1\right)\overset{\left(n\right)}{\to }0,\\ \; & {\bar{\gamma }}_{n}:=\left(1-\gamma_{n}\right)\left(1-\epsilon^{*}\right)\gamma_{n}^{\prime \prime }+\left(1-\gamma_{n}\right)\;\epsilon^{*}\frac{\gamma_{n}^{\prime }}{n}-\gamma_{n}^{\prime \prime \prime }. \end{array}$$

Equation (91) follows similarly to the proof of Theorem 1 in [71]. Equation (92) is obtained as follows:

$$\begin{array}{cc} \; & \frac{1}{n}H\left(S^{n}|M=0,V^{n},I_{U}\left(U^{n},\delta \right)=0,H=0\right) \end{array}$$

(98) $$\begin{array}{cc} \; & \geq \frac{1}{n}H\left(S^{n}|V^{n},H=0\right)-\frac{\gamma_{n}^{\prime }}{n} \end{array}$$

(99) $$\begin{array}{ccc} \; & >H_{P}\left(S|U,V\right)-\frac{\gamma_{n}^{\prime }}{n}. & \; \end{array}$$

Here, (98) is obtained by an application of Lemma 3; and (99) is due to the assumption that $H_{P}\left(S|U,V\right)<H_{P}\left(S|V\right)$.

It follows from Lemma 4 that $\rho_{n}^{*}\overset{\left(n\right)}{\to }0$, which in turn implies that

(100) $$\begin{array}{c} \frac{\gamma_{n}^{\prime }}{n}\overset{\left(n\right)}{\to }0. \end{array}$$

From (95), (97) and (100), we have that ${\bar{\gamma }}_{n}\overset{\left(n\right)}{\to }0$. Hence, Equation (94) implies that $\left(H_{P}\left(U\right),I_{P}\left(V;U\right),\Lambda_{0}^{*},\Lambda_{min}\right)\in R_{e}\left(\epsilon^{*}\right)$ for some $\Lambda_{0}^{*}>H_{P}\left(S|U,V\right)$. Since $\left(H_{P}\left(U\right),I_{P}\left(V;U\right),\Lambda_{0}^{*},\Lambda_{min}\right)\notin R_{e}$, this implies that in general, the strong converse does not hold for HT with an equivocation privacy constraint. The same counter-example can be used in a similar manner to show that the strong converse does not hold for HT with an average distortion privacy constraint either.

## 6. Conclusions

We have studied the DHT problem with a privacy constraint, with equivocation and average distortion under a causal disclosure assumption as the measures of privacy. We have established a single-letter inner bound on the rate-error exponent-equivocation and rate-error exponent-distortion trade-offs. We have also obtained the optimal rate-error exponent-equivocation and rate-error exponent-distortion trade-offs for two special cases, when the communication rate over the channel is zero, and for TACI under a privacy constraint. It is interesting to note that the strong converse for DHT does not hold when there is an additional privacy constraint in the system. Extending these results to the case when the communication between the observer and detector takes place over a noisy communication channel is an interesting avenue for future research. Yet another important topic worth exploring is the trade-off between rate, error probability and privacy in the finite sample regime for the setting considered in this paper.

## Abbreviations

The following abbreviations are used in this manuscript:

HT	Hypothesis testing
DHT	Distributed hypothesis testing
TACI	Testing against conditional independence
TAI	Testing against independence
DP	Differential privacy
KL	Kullback–Leibler
SHA	Shimokawa-Han-Amari

## Appendix A. Proof of Lemma 1

Please note that for a stochastic detector, the type I and type II error probabilities are linear functions of $P_{\hat{H}|M,V^{n}}$. As a result, for each fixed *n* and $f_{n}$, $\alpha_{n}\left(f_{n},g_{n}\right)$ and $\beta_{n}\left(f_{n},g_{n}\right)$ for a stochastic detector $g_{n}$ can be thought of as the type I and type II errors achieved by “time-sharing” among a finite number of deterministic detectors. To see this, consider some ordering on the elements of the set $M\times V^{n}$ and let $\nu_{i}:=P_{\hat{H}|M,V^{n}}\left(0|i\right)$, i∈[1:N], where *i* denotes the $i^{th}$ element of $M\times V^{n}$ and $N=|M\times V^{n}|$. Then, we can write

$$P_{\hat{H}|M,V^{n}}=\left[\begin{array}{cc} \nu_{1} & 1-\nu_{1}\\ \nu_{2} & 1-\nu_{2}\\ \vdots & \vdots \\ \nu_{N} & 1-\nu_{N} \end{array}\right].$$

Then, it is easy to see that $P_{\hat{H}|M,V^{n}}=\sum_{i=1}^{N}\nu_{i}I_{i}$, where $I_{i}:=\left[e_{i}\;1-e_{i}\right]$ and $e_{i}$ is an *N* length vector with 1 at the $i^{th}$ component and 0 elsewhere. Now, suppose $\left(\alpha_{n}^{\left(1\right)},\beta_{n}^{\left(1\right)}\right)$ and $\left(\alpha_{n}^{\left(2\right)},\beta_{n}^{\left(2\right)}\right)$ denote the pair of type I and type II error probabilities achieved by deterministic detectors $g_{n}^{\left(1\right)}$ and $g_{n}^{\left(2\right)}$, respectively. Let $A_{1,n}$ and $A_{2,n}$ denote their corresponding acceptance regions for $H_{0}$. Let $g_{n}^{\left(\theta \right)}$ denote the stochastic detector formed by using $g_{n}^{\left(1\right)}$ and $g_{n}^{\left(2\right)}$ with probabilities θ and 1−θ, respectively. From the above-mentioned linearity property, it follows that $g_{n}^{\left(\theta \right)}$ achieves type I and type II error probabilities of $\alpha_{n}\left(f_{n},g_{n}^{\left(\theta \right)}\right)=\theta \alpha_{n}^{\left(1\right)}+\left(1-\theta \right)\alpha_{n}^{\left(2\right)}$ and $\beta_{n}\left(f_{n},g_{n}^{\left(\theta \right)}\right)=\theta \beta_{n}^{\left(1\right)}+\left(1-\theta \right)\beta_{n}^{\left(2\right)}$, respectively. Let r(θ)=min(θ,1−θ). Then, for θ∈(0,1),

$$\begin{array}{c} -\frac{1}{n}\log\left(\beta_{n}\left(f_{n},g_{n}^{\left(\theta \right)}\right)\right)\leq \min\left(-\frac{1}{n}\log\beta_{n}^{\left(1\right)},-\frac{1}{n}\log\beta_{n}^{\left(2\right)}\right)-\frac{1}{n}\log\left(r\left(\theta \right)\right). \end{array}$$

Hence, either

$$\begin{array}{c} \alpha_{n}^{\left(1\right)}\leq \alpha_{n}\left(f_{n},g_{n}^{\left(\theta \right)}\right)and-\frac{1}{n}\log\beta_{n}^{\left(1\right)}\geq -\frac{1}{n}\log\left(\beta_{n}\left(f_{n},g_{n}^{\left(\theta \right)}\right)\right)+\frac{1}{n}\log\left(r\left(\theta \right)\right), \end{array}$$

or

$$\begin{array}{c} \alpha_{n}^{\left(2\right)}\leq \alpha_{n}\left(f_{n},g_{n}^{\left(\theta \right)}\right)and-\frac{1}{n}\log\beta_{n}^{\left(2\right)}\geq -\frac{1}{n}\log\left(\beta_{n}\left(f_{n},g_{n}^{\left(\theta \right)}\right)\right)+\frac{1}{n}\log\left(r\left(\theta \right)\right). \end{array}$$

Thus, since $\frac{1}{n}\log\left(r\left(\theta \right)\right)\overset{\left(n\right)}{\to }0$, a stochastic detector does not offer any advantage over deterministic detectors in the trade-off between the error exponent and the type I error probability.

## Appendix B. Proof of Lemma 2

Let ${\tilde{P}}_{S^{n}U^{n}V^{n}M{\hat{S}}^{n}}^{\left(C_{n},0\right)}=P_{S^{n}U^{n}V^{n}M}\;\prod_{i=1}^{n}{\tilde{P}}_{{\hat{S}}_{i}|M,V^{n},S^{i-1}}$ and ${\tilde{P}}_{S^{n}U^{n}V^{n}M{\hat{S}}^{n}}^{\left(C_{n},1\right)}=Q_{S^{n}U^{n}V^{n}M}\;\prod_{i=1}^{n}{\tilde{P}}_{{\hat{S}}_{i}|M,V^{n},S^{i-1}}$ denote the joint distribution of the r.v.’s $\left(S^{n},U^{n},V^{n},M,{\hat{S}}^{n}\right)$ under hypothesis $H_{0}$ and $H_{1}$, respectively, where ${\tilde{P}}_{{\hat{S}}_{i}|M,V^{n},S^{i-1}}$ denotes $g_{i,n}^{\left(r\right)}$ for i∈[n]. Then, we have

$$\begin{array}{ccc} \; & \min_{g_{i,n}^{\left(r\right)}}E\left[d\left(S^{n},{\hat{S}}^{n}\right)|H=j\right]\\ \; & =\min_{{\left\{{\tilde{P}}_{{\hat{S}}_{i}|M,V^{n},S^{i-1}}\right\}}_{i=1}^{n}}E_{{\tilde{P}}^{\left(j\right)}}\left[d\left(S^{n},{\hat{S}}^{n}\right)\right]\\ \; & =\min_{{\left\{{\tilde{P}}_{{\hat{S}}_{i}|M,V^{n},S^{i-1}}\right\}}_{i=1}^{n}}\frac{1}{n}\sum_{i=1}^{n}E_{{\tilde{P}}^{\left(j\right)}}\left[d\left(S_{i},{\hat{S}}_{i}\right)\right]\\ \; & =\frac{1}{n}\sum_{i=1}^{n}\sum_{\left(m,v^{n},s^{i-1}\right)}{\tilde{P}}_{MV^{n}S^{i-1}}^{\left(j\right)}\left(m,v^{n},s^{i-1}\right)\;\min_{{\tilde{P}}_{{\hat{S}}_{i}|M,V^{n},S^{i-1}}\left(\cdot |m,v^{n},s^{i-1}\right)}\;\sum_{{\hat{s}}_{i}}{\tilde{P}}_{{\hat{S}}_{i}|M,V^{n},S^{i-1}}\left({\hat{s}}_{i}|m,v^{n},s^{i-1}\right)\;\\ \; & \;\;\;\;\;\;\;\;\;\;\;\;\;\;\;E_{{\tilde{P}}_{S_{i}|M,V^{n},S^{i-1}}^{\left(j\right)}\left(\cdot |m,v^{n},s^{i-1}\right)}\left[d\left(S_{i},{\hat{s}}_{i}\right)\right]\\ \; & =\frac{1}{n}\sum_{i=1}^{n}\sum_{m,v^{n},s^{i-1}}{\tilde{P}}_{MV^{n}S^{i-1}}^{\left(j\right)}\left(m,v^{n},s^{i-1}\right)\;E_{{\tilde{P}}_{S_{i}|M,V^{n},S^{i-1}}^{\left(j\right)}\left(\cdot |m,v^{n},s^{i-1}\right)}\left[d\left(S_{i},\phi_{ij}\left(m,v^{n},s^{i-1}\right)\right)\right], & \; \end{array}$$

where

$$\begin{array}{c} \phi_{ij}\left(m,v^{n},s^{i-1}\right)=\underset{\hat{s}\in \hat{S}}{arg min}E_{{\tilde{P}}_{S_{i}|M,V^{n},S^{i-1}}^{\left(j\right)}\left(\cdot |m,v^{n},s^{i-1}\right)}\left[d(S_{i},\hat{s})\right]. \end{array}$$

Continuing, we have

(A1) $$\begin{array}{ccc} \; & \min_{g_{i,n}^{\left(r\right)}}E\left[d\left(S^{n},{\hat{S}}^{n}\right)|H=j\right]\\ \; & =\frac{1}{n}\sum_{i=1}^{n}\sum_{m,v^{n},s^{i-1}}{\tilde{P}}_{MV^{n}S^{i-1}}^{\left(j\right)}\left(m,v^{n},s^{i-1}\right)\;\min_{\phi_{i}\left(m,v^{n},s^{i-1}\right)}E_{{\tilde{P}}_{S_{i}|M,V^{n},S^{i-1}}^{\left(j\right)}\left(\cdot |m,v^{n},s^{i-1}\right)}\left[d\left(S_{i},\phi_{i}\left(m,v^{n},s^{i-1}\right)\right)\right]\\ \; & =\min_{{\left\{\phi_{i}\left(m,v^{n},s^{i-1}\right)\right\}}_{i=1}^{n}}\frac{1}{n}\sum_{i=1}^{n}E_{{\tilde{P}}^{\left(j\right)}}\left[d\left(S_{i},\phi_{i}\left(M,V^{n},S^{i-1}\right)\right)\right]. & \; \end{array}$$

This completes the proof.

## Appendix C. Proof of Lemma 4

We will first prove (15). Fix δ>0. For γ>0, define the following sets:

(A2) $$\begin{array}{cc} B_{0,\gamma }^{\delta } & :=\left\{y^{n}\in T_{{\left[P_{Y}\right]}_{\gamma }}^{n}:P_{Y^{n}}\left(y^{n}\right)\geq P_{Y^{n}|\Pi \left(X^{n},\delta ,P_{X}\right)}\left(y^{n}|0\right)\right\}, \end{array}$$

(A3) $$\begin{array}{cc} C_{0,\gamma }^{\delta } & :=\left\{y^{n}\in T_{{\left[P_{Y}\right]}_{\gamma }}^{n}:P_{Y^{n}}\left(y^{n}\right)<P_{Y^{n}|\Pi \left(X^{n},\delta ,P_{X}\right)}\left(y^{n}|0\right)\right\},\\ B_{1,\gamma }^{\delta } & :=\left\{y^{n}\in T_{{\left[Q_{Y}\right]}_{\gamma }}^{n}:Q_{Y^{n}}\left(y^{n}\right)\geq Q_{Y^{n}|\Pi \left(X^{n},\delta ,P_{X}\right)}\left(y^{n}|0\right)\right\},\\ C_{1,\gamma }^{\delta } & :=\left\{y^{n}\in T_{{\left[Q_{Y}\right]}_{\gamma }}^{n}:Q_{Y^{n}}\left(y^{n}\right)<Q_{Y^{n}|\Pi \left(X^{n},\delta ,P_{X}\right)}\left(y^{n}|0\right)\right\},\\ B_{2,\gamma }^{\delta } & :=\left\{y^{n}\in T_{{\left[Q_{Y}\right]}_{\gamma }}^{n}:Q_{Y^{n}}\left(y^{n}\right)\geq Q_{Y^{n}|\Pi \left(X^{n},\delta ,P_{X}\right)}\left(y^{n}|1\right)\right\},\\ C_{2,\gamma }^{\delta } & :=\left\{y^{n}\in T_{{\left[Q_{Y}\right]}_{\gamma }}^{n}:Q_{Y^{n}}\left(y^{n}\right)<Q_{Y^{n}|\Pi \left(X^{n},\delta ,P_{X}\right)}\left(y^{n}|1\right)\right\}. \end{array}$$

Then, we can write

(A4) $$\begin{array}{ccc} \; & ∥Q_{Y^{n}}\left(\cdot \right)-Q_{Y^{n}|\Pi \left(X^{n},\delta ,P_{X}\right)}\left(\cdot |1\right)∥\\ \; & =\frac{1}{2}\sum_{y^{n}}|Q_{Y^{n}}\left(y^{n}\right)-Q_{Y^{n}|\Pi \left(X^{n},\delta ,P_{X}\right)}\left(y^{n}|1\right)|\\ \; & =\frac{1}{2}\sum_{y^{n}\notin T_{{\left[Q_{Y}\right]}_{\gamma }}^{n}}|Q_{Y^{n}}\left(y^{n}\right)-Q_{Y^{n}|\Pi \left(X^{n},\delta ,P_{X}\right)}\left(y^{n}|1\right)|+\frac{1}{2}\sum_{y^{n}\in T_{{\left[Q_{Y}\right]}_{\gamma }}^{n}}|Q_{Y^{n}}\left(y^{n}\right)-Q_{Y^{n}|\Pi \left(X^{n},\delta ,P_{X}\right)}\left(y^{n}|1\right)|\\ \; & \leq \frac{1}{2}\sum_{y^{n}\notin T_{{\left[Q_{Y}\right]}_{\gamma }}^{n}}Q_{Y^{n}}\left(y^{n}\right)+Q_{Y^{n}|\Pi \left(X^{n},\delta ,P_{X}\right)}\left(y^{n}|1\right)+\frac{1}{2}\sum_{y^{n}\in T_{{\left[Q_{Y}\right]}_{\gamma }}^{n}}|Q_{Y^{n}}\left(y^{n}\right)-Q_{Y^{n}|\Pi \left(X^{n},\delta ,P_{X}\right)}\left(y^{n}|1\right)|. & \; \end{array}$$

Next, note that

(A5) $$\begin{array}{cc} Q_{Y^{n}|\Pi \left(X^{n},\delta ,P_{X}\right)}\left(y^{n}|1\right) & =Q_{Y^{n}}\left(y^{n}\right)\;\frac{Q_{\Pi \left(X^{n},\delta ,P_{X}\right)|Y^{n}}\left(1|y^{n}\right)}{Q_{\Pi (X^{n},\delta ,P_{X})}\left(1\right)}\leq \frac{Q_{Y^{n}}\left(y^{n}\right)}{Q_{\Pi (X^{n},\delta ,P_{X})}\left(1\right)}\leq 2Q_{Y^{n}}\left(y^{n}\right), \end{array}$$

for sufficiently large *n* (depending on |X|), since $Q_{\Pi (X^{n},\delta ,P_{X})}\left(1\right)\overset{\left(n\right)}{\to }1$. Thus, for *n* large enough,

(A6) $$\begin{array}{cc} \; & \sum_{y^{n}\notin T_{{\left[Q_{Y}\right]}_{\gamma }}^{n}}Q_{Y^{n}}\left(y^{n}\right)+Q_{Y^{n}|\Pi \left(X^{n},\delta ,P_{X}\right)}\left(y^{n}|1\right)\leq 3\sum_{y^{n}\notin T_{{\left[Q_{Y}\right]}_{\gamma }}^{n}}Q_{Y^{n}}\left(y^{n}\right)\leq e^{-n\Omega (\gamma )}. \end{array}$$

We can bound the last term in (A4) as follows:

(A7) $$\begin{array}{cc} \; & \sum_{y^{n}\in T_{{\left[Q_{Y}\right]}_{\gamma }}^{n}}|Q_{Y^{n}}\left(y^{n}\right)-Q_{Y^{n}|\Pi \left(X^{n},\delta ,P_{X}\right)}\left(y^{n}|1\right)|\\ \; & =\sum_{y^{n}\in B_{2,\gamma }^{\delta }}Q_{Y^{n}}\left(y^{n}\right)-Q_{Y^{n}|\Pi \left(X^{n},\delta ,P_{X}\right)}\left(y^{n}|1\right)+\sum_{y^{n}\in C_{2,\gamma }^{\delta }}Q_{Y^{n}|\Pi \left(X^{n},\delta ,P_{X}\right)}\left(y^{n}|1\right)-Q_{Y^{n}}\left(y^{n}\right)\\ \; & =\sum_{y^{n}\in B_{2,\gamma }^{\delta }}Q_{Y^{n}}\left(y^{n}\right)-Q_{Y^{n}|\Pi \left(X^{n},\delta ,P_{X}\right)}\left(y^{n}|1\right)+\sum_{y^{n}\in C_{2,\gamma }^{\delta }}Q_{Y^{n}|\Pi \left(X^{n},\delta ,P_{X}\right)}\left(y^{n}|1\right)-Q_{Y^{n}}\left(y^{n}\right)\\ \; & =\sum_{y^{n}\in B_{2,\gamma }^{\delta }}Q_{Y^{n}}\left(y^{n}\right)\left(1-\frac{Q_{Y^{n}|\Pi \left(X^{n},\delta ,P_{X}\right)}\left(y^{n}|1\right)}{Q_{Y^{n}}\left(y^{n}\right)}\right)+\sum_{y^{n}\in C_{2,\gamma }^{\delta }}Q_{Y^{n}}\left(y^{n}\right)\left(\frac{Q_{Y^{n}|\Pi \left(X^{n},\delta ,P_{X}\right)}\left(y^{n}|1\right)}{Q_{Y^{n}}\left(y^{n}\right)}-1\right)\\ \; & =\sum_{y^{n}\in B_{2,\gamma }^{\delta }}Q_{Y^{n}}\left(y^{n}\right)\left(1-\frac{Q_{\Pi \left(X^{n},\delta ,P_{X}\right)|Y^{n}}\left(1|y^{n}\right)}{Q_{\Pi (X^{n},\delta ,P_{X})}\left(1\right)}\right)+\sum_{y^{n}\in C_{2,\gamma }^{\delta }}Q_{Y^{n}}\left(y^{n}\right)\left(\frac{Q_{\Pi \left(X^{n},\delta ,P_{X}\right)|Y^{n}}\left(1|y^{n}\right)}{Q_{\Pi (X^{n},\delta ,P_{X})}\left(1\right)}-1\right) \end{array}$$

(A8) $$\begin{array}{ccc} \; & \leq \sum_{y^{n}\in B_{2,\gamma }^{\delta }}Q_{Y^{n}}\left(y^{n}\right)\left(1-Q_{\Pi \left(X^{n},\delta ,P_{X}\right)|Y^{n}}\left(1|y^{n}\right)\right)+\sum_{y^{n}\in C_{2,\gamma }^{\delta }}Q_{Y^{n}}\left(y^{n}\right)\left(\frac{1}{Q_{\Pi (X^{n},\delta ,P_{X})}\left(1\right)}-1\right). & \; \end{array}$$

Let $P_{\tilde{Y}}$ denote the type of $y^{n}$ and define

$$\begin{array}{c} E_{n}\left(\delta ,\gamma \right):=\min_{P_{\tilde{Y}}\in P_{n}\left(T_{{\left[Q_{Y}\right]}_{\gamma }}^{n}\right)}\min_{P_{\tilde{X}}\in P_{n}\left(T_{{\left[P_{X}\right]}_{\delta }}^{n}\right)}D(P_{\tilde{X}|\tilde{Y}}\left|\right|Q_{X|Y}\left|P_{\tilde{Y}}\right). \end{array}$$

Then, for $y^{n}\in T_{{\left[Q_{Y}\right]}_{\gamma }}^{n}$, arbitrary $\tilde{\gamma }>0$ and *n* sufficiently large (depending on |X|,|Y|,δ,γ), it follows from ([60], Lemma 2.6) that

(A9) $$\begin{array}{cc} \; & Q_{\Pi \left(X^{n},\delta ,P_{X}\right)|Y^{n}}\left(1|y^{n}\right)\geq 1-e^{-n\left(E_{n}\left(\delta ,\gamma \right)-\tilde{\gamma }\right)}, \end{array}$$

(A10) $$\begin{array}{cc} \mathrm{and}\; & Q_{\Pi (X^{n},\delta ,P_{X})}\left(1\right)\geq 1-e^{-n(D(P_{X}\left|\right|Q_{X})-\tilde{\gamma })}. \end{array}$$

From (A4), (A6) and (A8)–(A10), it follows that

(A11) $$\begin{array}{c} ∥Q_{Y^{n}}\left(\cdot \right)-Q_{Y^{n}|\Pi \left(X^{n},\delta ,P_{X}\right)}\left(\cdot |1\right)∥\leq e^{-n\Omega (\gamma )}+e^{-n\left(E_{n}\left(\delta ,\gamma \right)-\tilde{\gamma }\right)}+e^{-n(D(P_{X}\left|\right|Q_{X})-\tilde{\gamma })}. \end{array}$$

We next show that $E_{n}\left(\delta ,\gamma \right)>0$ for sufficiently small δ>0 and γ>0. This would imply that the R.H.S of (A11) converges exponentially to zero (for $\tilde{\gamma }$ small enough) with exponent $\bar{\delta }:=\min\left(\Omega \left(\gamma \right),E_{n}\left(\delta ,\gamma \right)-\tilde{\gamma },D(P_{X}\left|\right|Q_{X})-\tilde{\gamma }\right)$, thus proving (15). We can write,

(A12) $$\begin{array}{cc} E_{n}\left(\delta ,\gamma \right) & \geq \min_{P_{\tilde{Y}}\in T_{{\left[Q_{Y}\right]}_{\gamma }}^{n}}\min_{P_{\tilde{X}}\in T_{{\left[P_{X}\right]}_{\delta }}^{n}}D(P_{\tilde{X}}\left|\right|{\hat{Q}}_{X}) \end{array}$$

(A13) $$\begin{array}{cc} \; & \geq 2\left[\min_{P_{\tilde{Y}}\in T_{{\left[Q_{Y}\right]}_{\gamma }}^{n}}\min_{P_{\tilde{X}}\in T_{{\left[P_{X}\right]}_{\delta }}^{n}}{∥P_{\tilde{X}}-{\hat{Q}}_{X}∥}^{2}\right], \end{array}$$

where

$$\begin{array}{c} {\hat{Q}}_{X}\left(x\right):=\sum_{y}P_{\tilde{Y}}\left(y\right)Q_{X|Y}\left(x|y\right). \end{array}$$

Here, (A12) follows due to the convexity of KL divergence (A13) is due to Pinsker’s inequality [60]. We also have from the triangle inequality satisfied by total variation that,

$$\begin{array}{c} ∥P_{\tilde{X}}-{\hat{Q}}_{X}∥\geq ∥P_{X}-Q_{X}∥-∥P_{\tilde{X}}-P_{X}∥-∥{\hat{Q}}_{X}-Q_{X}∥. \end{array}$$

For $y^{n}\in T_{{\left[Q_{Y}\right]}_{\gamma }}^{n}$,

$$\begin{array}{c} ∥{\hat{Q}}_{X}-Q_{X}∥\leq ∥Q_{X|Y}P_{\tilde{Y}}-Q_{XY}∥\leq ∥P_{\tilde{Y}}-Q_{Y}∥\leq O\left(\gamma \right). \end{array}$$

Also, for $P_{\tilde{X}}\in T_{{\left[P_{X}\right]}_{\delta }}^{n}$,

$$\begin{array}{c} ∥P_{\tilde{X}}-P_{X}∥\leq O\left(\delta \right). \end{array}$$

Hence,

$$\begin{array}{cc} E_{n}\left(\delta ,\gamma \right) & \geq 2{\left(∥P_{X}-Q_{X}∥-O\left(\gamma \right)-O\left(\delta \right)\right)}^{2}. \end{array}$$

Since $P_{X}\neq Q_{X}$, $E_{n}\left(\delta ,\gamma \right)>0$ for sufficiently small γ>0 and δ>0. This completes the proof of (15).

We next prove (17). Similar to (A4) and (A5), we have,

(A14) $$\begin{array}{ccc} \; & ∥P_{Y^{n}}\left(\cdot \right)-P_{Y^{n}|\Pi \left(X^{n},\delta ,P_{X}\right)}\left(\cdot |0\right)∥\\ \; & \leq \frac{1}{2}\sum_{y^{n}\notin T_{{\left[P_{Y}\right]}_{\gamma }}^{n}}\left[P_{Y^{n}}\left(y^{n}\right)+P_{Y^{n}|\Pi \left(X^{n},\delta ,P_{X}\right)}\left(y^{n}|0\right)\right]+\frac{1}{2}\sum_{y^{n}\in T_{{\left[P_{Y}\right]}_{\gamma }}^{n}}|P_{Y^{n}}\left(y^{n}\right)-P_{Y^{n}|\Pi \left(X^{n},\delta ,P_{X}\right)}\left(y^{n}|0\right)|, & \; \end{array}$$

and

(A15) $$\begin{array}{c} P_{Y^{n}|\Pi \left(X^{n},\delta ,P_{X}\right)}\left(y^{n}|0\right)\leq 2P_{Y^{n}}\left(y^{n}\right), \end{array}$$

since $P_{\Pi (X^{n},\delta ,P_{X})}\left(0\right)\overset{\left(n\right)}{\to }1$.

Also, for $\gamma <\frac{\delta }{\left|Y\right|}$ and sufficiently large *n* (depending on δ,γ,|X|,|Y|), we have

(A16) $$\begin{array}{cc} \; & \sum_{y^{n}\in T_{{\left[P_{Y}\right]}_{\gamma }}^{n}}|P_{Y^{n}}\left(y^{n}\right)-P_{Y^{n}|\Pi \left(X^{n},\delta ,P_{X}\right)}\left(y^{n}|0\right)|\\ \; & =\sum_{y^{n}\in B_{0,\gamma }^{\delta }}P_{Y^{n}}\left(y^{n}\right)-P_{Y^{n}|\Pi \left(X^{n},\delta ,P_{X}\right)}\left(y^{n}|0\right)+\sum_{y^{n}\in C_{0,\gamma }^{\delta }}P_{Y^{n}|\Pi \left(X^{n},\delta ,P_{X}\right)}\left(y^{n}|0\right)-P_{Y^{n}}\left(y^{n}\right)\\ \; & \leq \sum_{y^{n}\in B_{0,\gamma }^{\delta }}P_{Y^{n}}\left(y^{n}\right)\left(1-P_{\Pi \left(X^{n},\delta ,P_{X}\right)|Y^{n}}\left(0|y^{n}\right)\right)+\sum_{y^{n}\in C_{0,\gamma }^{\delta }}P_{Y^{n}}\left(y^{n}\right)\left(\frac{1}{P_{\Pi (X^{n},\delta ,P_{X})}\left(0\right)}-1\right)\\ \; & \leq \sum_{y^{n}\in B_{0,\gamma }^{\delta }}P_{Y^{n}}\left(y^{n}\right)e^{-n\Omega (\delta -\gamma |Y|)}+\sum_{y^{n}\in C_{0,\gamma }^{\delta }}P_{Y^{n}}\left(y^{n}\right)e^{-n\Omega (\delta )} \end{array}$$

(A17) $$\begin{array}{ccc} \; & \leq e^{-n\Omega (\delta -\gamma |Y|)}, & \; \end{array}$$

where to obtain (A16), we used

(A18) $$\begin{array}{cc} \; & P_{\Pi (X^{n},\delta ,P_{X})}\left(0\right)\geq 1-e^{-n\Omega (\delta )}, \end{array}$$

(A19) $$\begin{array}{cc} \mathrm{and}\; & P_{\Pi \left(X^{n},\delta ,P_{X}\right)|Y^{n}}\left(0|y^{n}\right)\geq 1-e^{-n\Omega (\delta -\gamma |Y|)},fory^{n}\in B_{0,\gamma }^{\delta }and\gamma <\frac{\delta }{\left|Y\right|}. \end{array}$$

Here, (A18) follows from ([60], Lemma 2.12), and (A19) follows from ([60], Lemmas 2.10 and 2.12), respectively. Thus, from (A14), (A15) and (A17), we can write that,

$$\begin{array}{c} ∥P_{Y^{n}}\left(\cdot \right)-P_{Y^{n}|\Pi \left(X^{n},\delta ,P_{X}\right)}\left(\cdot |0\right)∥\leq e^{-n\Omega (\gamma )}+e^{-n\Omega (\delta -\gamma |Y|)}\overset{\left(n\right)}{\to }0. \end{array}$$

This completes the proof of (17). The proof of (16) is exactly the same as (17), with the only difference that the sets $B_{1,\gamma }^{\delta }$ and $C_{1,\gamma }^{\delta }$ are used in place of $B_{0,\gamma }^{\delta }$ and $C_{0,\gamma }^{\delta }$, respectively.

## Appendix D. Proof of Theorems 1 and 2

We describe the encoding and decoding operations which are the same for both Theorems 1 and 2. Fix positive numbers (small) η,δ>0, and let $\delta^{\prime }:=\frac{\delta }{2},\;\hat{\delta }:=\left|U\right|\delta ,\;\tilde{\delta }:=2\delta$ and $\bar{\delta }:=\frac{\delta^{\prime }}{\left|V\right|}$.

**Codebook Generation**: Fix a finite alphabet W and a conditional distribution $P_{W|U}$. Let $B_{n}=\left\{W^{n}\left(j\right),\;j\in \left[M_{n}^{\prime }\right]\right\}$, $M_{n}^{\prime }:=e^{n(I_{P}\left(U:W\right)+\eta )}$, denote a random codebook such that each $W^{n}\left(j\right)$ is randomly and independently generated according to distribution $\prod_{i=1}^{n}P_{W}\left(w_{i}\right)$, where

$$\begin{array}{c} P_{W}\left(w\right)=\sum_{u\in U}P_{U}\left(u\right)P_{W|U}\left(w|u\right). \end{array}$$

Denote a realization of $B_{n}$ by $B_{n}$ and the support of $B_{n}$ by $B_{n}$.

**Encoding**: For a given codebook $B_{n}$, let

(A20) $$\begin{array}{cc} \; & P_{E_{u}}^{\left(B_{n}\right)}\left(j|u^{n}\right):=\frac{\prod_{i=1}^{n}P_{U|W}\left(u_{i}|w_{i}\left(j\right)\right)}{\sum_{j^{\prime }}\prod_{i=1}^{n}P_{U|W}\left(u_{i}|w_{i}\left(j^{\prime }\right)\right))}, \end{array}$$

denote the likelihood encoding function. If $I_{P}\left(U;W\right)+\eta +\left|U||W\right|\frac{\log(n+1)}{n}>R$, the observer performs uniform random binning on the indices in $\left[M_{n}^{\prime }\right]$, i.e., for each $j\in \left[M_{n}^{\prime }\right]$, it selects an index uniformly at random from the set ${\tilde{M}}_{n}:=\left[e^{n\left(R-\left|U||W\right|\frac{\log(n+1)}{n}\right)}\right]$. Denote the random binning function by $f_{B}$ and a realization of it by $f_{B}$. If $I_{P}\left(U;W\right)+\eta +\left|U||W\right|\frac{\log(n+1)}{n}\leq R$, set $f_{B}$ as the identity function with probability one, i.e., $f_{B}\left(j\right)=j$. If $u^{n}\in T_{{\left[P_{U}\right]}_{\delta^{\prime }}}^{n}$, then the observer outputs the message $m=(t,f_{B}\left(j\right))$ if $I_{P}\left(U;W\right)+\eta +\left|U||W\right|\frac{\log(n+1)}{n}>R$ or m=(t,j) otherwise, where $j\in [M_{n}^{\prime }]$ is chosen randomly with probability $P_{E_{u}}^{\left(B_{n}\right)}\left(j|u^{n}\right)$ and *t* denotes the index of the joint type of $\left(u^{n},w^{n}\left(j\right)\right)$ in the set of types $P^{n}\left(U\times W\right)$. If $u^{n}\notin T_{{\left[P_{U}\right]}_{\delta^{\prime }}}^{n}$, the observer outputs the *error* message M=0. Please note that $\left|M\right|\leq e^{nR}$ since the total number of types in $P^{n}\left(U\times W\right)$ is upper bounded by ${\left(n+1\right)}^{\left|U||W\right|}$ ([60], Lemma 2.2). Let $C_{n}:=\left(B_{n},f_{B}\right)$, and let $C_{n}=\left(B_{n},f_{B}\right)$ and $\mu_{n}\left(\cdot \right)$ denote its realization and probability distribution, respectively. For a given $C_{n}$, let $f_{n}^{\left(C_{n}\right)}$ represent the encoder induced by the above operations, where $f_{n}^{\left(C_{n}\right)}:U^{n}\to P\left(M\right)$ and $M:=[e^{nR}]$.

**Decoding**: If M=0 or $t\notin T_{{\left[P_{UW}\right]}_{\delta }}^{n}$, $\hat{H}=1$ is declared. Else, given $m=(t,f_{B}\left(j\right))$ and $V^{n}=v^{n}$, the detector decodes for a codeword ${\hat{w}}^{n}:=w^{n}\left(\hat{j}\right)\in T_{{\left[P_{W}\right]}_{\hat{\delta }}}^{n}$ in the codebook $B_{n}$ such that

$$\begin{array}{cc} \hat{j} & =\underset{\begin{array}{c} l:\;f_{B}\left(l\right)=f_{B}\left(j\right),\\ w^{n}\left(l\right)\in T_{{\left[P_{W}\right]}_{\hat{\delta }}}^{n} \end{array}}{arg min}H_{e}\left(w^{n}\left(l\right)|v^{n}\right),ifI_{P}\left(U;W\right)+\eta +\frac{1}{n}|U||W|\log\left(n+1\right)>R,\\ \hat{j} & =j,\mathrm{otherwise}. \end{array}$$

Denote the above decoding rule by $P_{\mathrm{ED}}^{\left(C_{n}\right)}$, where $P_{\mathrm{ED}}^{\left(C_{n}\right)}:M\times V^{n}\to J$. The detector declares $\hat{H}=0$ if $\left({\hat{w}}^{n},v^{n}\right)\in T_{{\left[P_{WV}\right]}_{\tilde{\delta }}}^{n}$ and $\hat{H}=1$ otherwise. Let $g_{n}^{\left(C_{n}\right)}:M\times V^{n}\to \hat{H}$ stand for the decision rule induced by the above operations.

**System induced distributions and auxiliary distributions**:

The system induced probability distribution when H=0 is given by

$$\begin{array}{cc} \; & {\tilde{P}}^{\left(C_{n},0\right)}\left(s^{n},u^{n},v^{n},j,w^{n},m,\hat{j},{\hat{w}}^{n}\right)\\ \; & =\left[\prod_{i=1}^{n}P_{SUV}\left(s_{i},u_{i},v_{i},z_{i}\right)\right]P_{E_{u}}^{\left(B_{n}\right)}\left(j|u^{n}\right)1\left(w^{n}\left(j\right)=w^{n}\right)1\left(f_{B}\left(j\right)=m\right)\;1\left(\hat{j}=P_{\mathrm{ED}}^{\left(C_{n}\right)}\left(m,v^{n}\right)\right) \end{array}$$

(A21) $$\begin{array}{cc} \;1\left(w^{n}\left(\hat{j}\right)={\hat{w}}^{n}\right),\;\;\;\;\;\;\;\;\;\;\mathrm{if}\;u^{n}\in T_{{\left[P_{U}\right]}_{\delta^{\prime }}}^{n}, & \; \end{array}$$

and

(A22) $$\begin{array}{cc} {\tilde{P}}^{\left(C_{n},0\right)}\left(s^{n},u^{n},v^{n},m\right)=\left[\prod_{i=1}^{n}P_{SUV}\left(s_{i},u_{i},v_{i}\right)\right]1\left(m=0\right),\;\;\;\mathrm{if}\;u^{n}\notin T_{{\left[P_{U}\right]}_{\delta^{\prime }}}^{n}. & \; \end{array}$$

Consider two auxiliary distribution $\tilde{\Psi }$ and Ψ given by

(A23) $$\begin{array}{ccc} \; & {\tilde{\Psi }}^{\left(C_{n},0\right)}\left(s^{n},u^{n},v^{n},j,w^{n},m,\hat{j},{\hat{w}}^{n}\right)\\ \; & :=\left[\prod_{i=1}^{n}P_{SUV}\left(s_{i},u_{i},v_{i}\right)\right]P_{E_{u}}^{\left(B_{n}\right)}\left(j|u^{n}\right)1\left(w^{n}\left(j\right)=w^{n}\right)1\left(f_{B}\left(j\right)=m\right)\;1\left(\hat{j}=P_{\mathrm{ED}}^{\left(C_{n}\right)}\left(m,v^{n}\right)\right)\\ \; & \;\;1(w^{n}\left(\hat{j}\right)={\hat{w}}^{n}), & \; \end{array}$$

and

(A24) $$\begin{array}{ccc} \; & \Psi^{\left(C_{n},0\right)}\left(s^{n},u^{n},v^{n},j,w^{n},m,\hat{j},{\hat{w}}^{n}\right)\\ \; & :=\frac{1}{M_{n}^{\prime }}\;1\left(w^{n}\left(j\right)=w^{n}\right)\;\left[\prod_{i=1}^{n}P_{U|W}\left(u_{i}|w_{i}\right)\right]\left[\prod_{i=1}^{n}P_{VS|U}\left(v_{i},s_{i}|u_{i}\right)\right]\;1\left(f_{B}\left(j\right)=m\right)\;1\left(\hat{j}=P_{\mathrm{ED}}^{\left(C_{n}\right)}\left(m,v^{n}\right)\right)\\ \; & \;1(w^{n}\left(\hat{j}\right)={\hat{w}}^{n}). & \; \end{array}$$

Let ${\tilde{P}}^{\left(C_{n},1\right)}$ and ${\tilde{\Psi }}^{\left(C_{n},1\right)}$ denote probability distributions under H=1 defined by the R.H.S. of (A21)–(A23) with $P_{SUV}$ replaced by $Q_{SUV}$, and let $\Psi^{\left(C_{n},1\right)}$ denote the R.H.S. of (A24) with $P_{VS|U}$ replaced by $Q_{VS|U}$. Please note that the encoder $f_{n}^{\left(C_{n}\right)}$ is such that $P_{E_{u}}^{\left(B_{n}\right)}\left(j|u^{n}\right)=\Psi^{\left(C_{n},0\right)}\left(j|u^{n}\right)$ and hence, the only difference between the joint distribution $\Psi^{\left(C_{n},0\right)}$ and ${\tilde{\Psi }}^{\left(C_{n},0\right)}$ is the marginal distribution of $U^{n}$. By the soft-covering lemma [62,64], it follows that for some $\gamma_{1}>0$,

(A25) $$\begin{array}{c} E_{\mu_{n}}\left[∥\Psi_{U^{n}}^{\left(C_{n},0\right)}-{\tilde{\Psi }}_{U^{n}}^{\left(C_{n},0\right)}∥\right]\leq e^{-n\gamma_{1}}\overset{\left(n\right)}{\to }0. \end{array}$$

Hence, from ([43], Property 2(d)), it follows that

(A26) $$\begin{array}{c} E_{\mu_{n}}\left[∥\Psi^{\left(C_{n},0\right)}-{\tilde{\Psi }}^{\left(C_{n},0\right)}∥\right]\leq e^{-n\gamma_{1}}. \end{array}$$

Also, note that the only difference between the distributions ${\tilde{P}}^{\left(C_{n},0\right)}$ and ${\tilde{\Psi }}^{\left(C_{n},0\right)}$ is $P_{E_{u}}^{\left(B_{n}\right)}$ when $U^{n}\notin T_{{\left[P_{U}\right]}_{\delta^{\prime }}}^{n}$. Since

(A27) $$\begin{array}{c} P\left(U^{n}\notin T_{{\left[P_{U}\right]}_{\delta^{\prime }}}^{n}|H=0\right)\leq e^{-n\Omega (\delta^{\prime })}, \end{array}$$

it follows that

(A28) $$\begin{array}{c} E_{\mu_{n}}\left[∥{\tilde{P}}^{\left(C_{n},0\right)}-{\tilde{\Psi }}^{\left(C_{n},0\right)}∥\right]\leq e^{-n\Omega (\delta^{\prime })}. \end{array}$$

Equations (A26) and (A28) together imply via ([43], Property 2(c)) that

(A29) $$\begin{array}{c} E_{\mu_{n}}\left[∥{\tilde{P}}^{\left(C_{n},0\right)}-\Psi^{\left(C_{n},0\right)}∥\right]\leq e^{-n\Omega (\delta^{\prime })}+e^{-n\gamma_{1}}\overset{\left(n\right)}{\to }0. \end{array}$$

Please note that for l∈{0,1}, the joint distribution $\Psi^{\left(C_{n},l\right)}$ satisfies

(A30) $$\begin{array}{c} S_{i}-\left(w_{i}\left(J\right),V_{i}\right)-\left(M,w^{n}\left(J\right),V^{n},S^{i-1}\right),\;i\in \left[n\right]. \end{array}$$

Also, since $I_{P}\left(U;W\right)+\eta >0$, by the application of soft-covering lemma,

(A31) $$\begin{array}{cc} E_{\mu_{n}}\left[\sum_{i=1}^{n}∥P_{W}-\Psi_{W_{i}\left(J\right)}^{\left(C_{n},l\right)}∥|H=l\right] & \leq e^{-\gamma_{3}n}\overset{\left(n\right)}{\to }0,\;l=0,1, \end{array}$$

for some $\gamma_{3}>0$.

If $Q_{U}=P_{U}$, then it again follows from the soft-covering lemma that

(A32) $$\begin{array}{c} E_{\mu_{n}}\left[∥\Psi_{U^{n}}^{\left(C_{n},1\right)}-{\tilde{\Psi }}_{U^{n}}^{\left(C_{n},1\right)}∥\right]\leq e^{-\gamma_{1}n}\overset{\left(n\right)}{\to }0, \end{array}$$

thereby implying that

(A33) $$\begin{array}{c} E_{\mu_{n}}\left[∥\Psi^{\left(C_{n},1\right)}-{\tilde{\Psi }}^{\left(C_{n},1\right)}∥\right]\leq e^{-\gamma_{1}n}. \end{array}$$

Also, note that the only difference between the distributions ${\tilde{P}}^{\left(C_{n},1\right)}$ and ${\tilde{\Psi }}^{\left(C_{n},1\right)}$ is $P_{E_{u}}^{\left(B_{n}\right)}$ when $U^{n}\notin T_{{\left[P_{U}\right]}_{\delta^{\prime }}}^{n}$. Since $Q_{U}=P_{U}$ implies $P\left(U^{n}\notin T_{{\left[P_{U}\right]}_{\delta^{\prime }}}^{n}|H=1\right)\leq e^{-n\Omega (\delta^{\prime })}$, it follows that

(A34) $$\begin{array}{c} E_{\mu_{n}}\left[∥{\tilde{P}}^{\left(C_{n},1\right)}-{\tilde{\Psi }}^{\left(C_{n},1\right)}∥\right]\leq e^{-n\Omega (\delta^{\prime })}. \end{array}$$

Equations (A33) and (A34) together imply that

(A35) $$\begin{array}{c} E_{\mu_{n}}\left[∥{\tilde{P}}^{\left(C_{n},1\right)}-\Psi^{\left(C_{n},1\right)}∥\right]\leq e^{-n\Omega (\delta^{\prime })}+e^{-\gamma_{1}n}\overset{\left(n\right)}{\to }0. \end{array}$$

Let ${\bar{P}}_{{\tilde{P}}^{\left(C_{n},0\right)}}=E_{\mu_{n}}\left[P_{{\tilde{P}}^{\left(C_{n},0\right)}}\right]$ and ${\bar{P}}_{{\tilde{P}}^{\left(C_{n},1\right)}}=E_{\mu_{n}}\left[P_{{\tilde{P}}^{\left(C_{n},1\right)}}\right]$ denote the expected probability measure (random coding measure) induced by PMF’s ${\tilde{P}}^{\left(C_{n},0\right)}$ and ${\tilde{P}}^{\left(C_{n},1\right)}$, respectively. Then, note that from (A24), (A29), (A31) and the weak law of large numbers,

(A36) $$\begin{array}{c} {\bar{P}}_{{\tilde{P}}^{\left(C_{n},0\right)}}\left(\left(U^{n},W^{n}\left(J\right)\right)\in T_{{\left[P_{UW}\right]}_{\delta }}^{n}\right)\geq 1-e^{-n\Omega (\delta )}\overset{\left(n\right)}{\to }1. \end{array}$$

**Analysis of type I and type II error probabilities**:

We analyze type I and type II error probabilities of the coding scheme mentioned above averaged over the random ensemble $C_{n}$.

**Type I error probability**:

Please note that a type I error occurs only if one of the following events occur:

$$\begin{array}{ccc} E_{\mathrm{TE}} & =\left\{\left(U^{n},V^{n}\right)\notin T_{{\left[P_{UV}\right]}_{\bar{\delta }}}^{n}\right\},\\ E_{\mathrm{SE}} & =\left\{T\notin P_{n}\left(T_{{\left[P_{UW}\right]}_{\delta }}^{n}\right)\right\},\\ E_{\mathrm{ME}} & =\left\{\left(V^{n},W^{n}\left(J\right)\right)\notin T_{{\left[P_{VW}\right]}_{\tilde{\delta }}}^{n}\right\},\\ E_{\mathrm{DE}} & =\{\exists \;l\in \left[e^{n(I_{P}\left(U;W\right)+\eta )}\right],\;l\neq J:f_{B}\left(l\right)=f_{B}\left(J\right),\;W^{n}\left(l\right)\in T_{{\left[P_{W}\right]}_{\hat{\delta }}}^{n},\\ \; & \;H_{e}\left(W^{n}\left(l\right)|V^{n}\right)\leq H_{e}\left(W^{n}\left(J\right)|V^{n}\right)\}. & \; \end{array}$$

Let $E:=E_{\mathrm{TE}}\cup E_{\mathrm{SE}}\cup E_{\mathrm{ME}}\cup E_{\mathrm{DE}}$. Then, the expected type I error probability over $C_{n}$ be upper bounded as

(A37) $$\begin{array}{c} E_{\mu_{n}}\left[\alpha_{n}\left(f_{n}^{\left(C_{n}\right)},g_{n}^{\left(C_{n}\right)}\right)\right]\leq {\bar{P}}_{{\tilde{P}}^{\left(C_{n},0\right)}}\left(E\right). \end{array}$$

Please note that ${\bar{P}}_{{\tilde{P}}^{\left(C_{n},0\right)}}\left(E_{\mathrm{TE}}\right)$ tends to 0 asymptotically by the weak law of large numbers. From (A36), ${\bar{P}}_{{\tilde{P}}^{\left(C_{n},0\right)}}\left(E_{\mathrm{SE}}\right)\overset{\left(n\right)}{\to }0$. Given $E_{\mathrm{SE}}^{c}$ and $E_{\mathrm{TE}}^{c}$ holds, it follows from the Markov chain relation V−U−W and the Markov lemma [68] that ${\bar{P}}_{{\tilde{P}}^{\left(C_{n},0\right)}}\left(E_{\mathrm{ME}}\right)\overset{\left(n\right)}{\to }0$. Also, as in the proof of Theorem 2 in [13], it follows that

(A38) $$\begin{array}{cc} \; & {\bar{P}}_{{\tilde{P}}^{\left(C_{n},0\right)}}\left(E_{\mathrm{DE}}|\;V^{n}=v^{n},W^{n}\left(J\right)=w^{n},E_{\mathrm{ME}}^{c}\cap E_{\mathrm{SE}}^{c}\cap E_{\mathrm{TE}}^{c}\right)\leq e^{-n\left(R-I_{P}\left(U;W|V\right)-\delta_{n}^{\left(1\right)}\right)}, \end{array}$$

where $\delta_{n}^{\left(1\right)}\overset{\left(n\right)}{\to }\eta +O\left(\delta \right)$. Thus, if $R>I_{P}\left(U;W|V\right)$, it follows by choosing η=O(δ) that for δ>0 small enough, the R.H.S. of (A38) tends to zero asymptotically. By the union bound on probability, the R.H.S. of (A37) tends to zero.

**Type II error probability**:

Let $\delta^{\prime \prime }=\left|W\right|\tilde{\delta }$. Please note that a type II error occurs only if $V^{n}\in T_{{\left[P_{V}\right]}_{\delta^{\prime \prime }}}^{n}$ and M≠0, i.e., $U^{n}\in T_{{\left[P_{U}\right]}_{\delta^{\prime }}}^{n}$ and $T\in T_{{\left[P_{UW}\right]}_{\delta }}^{n}$. Hence, we can restrict the type II error analysis to only such $\left(U^{n},V^{n}\right)$. Denoting the event that a type II error occurs by $D_{0}$, we have

(A39) $$\begin{array}{cc} E_{\mu_{n}}\left[\beta_{n}\left(f_{n}^{\left(C_{n}\right)},g_{n}^{\left(C_{n}\right)}\right)\right]=\sum_{u^{n},v^{n}}{\bar{P}}_{{\tilde{P}}^{\left(C_{n},1\right)}}\left(U^{n}=u^{n},V^{n}=v^{n}\right)\;{\bar{P}}_{{\tilde{P}}^{\left(C_{n},1\right)}}\left(D_{0}|U^{n}=u^{n},V^{n}=v^{n}\right). & \; \end{array}$$

Let $E_{\mathrm{NE}}:=E_{\mathrm{SE}}^{c}\cap \left\{V^{n}\in T_{{\left[V\right]}_{\delta^{\prime \prime }}}^{n}\right\}\cap \left\{U^{n}\in T_{{\left[U\right]}_{\delta^{\prime }}}^{n}\right\}$. The last term in (A39) can be upper bounded as follows:

(A40) $$\begin{array}{cc} \; & {\bar{P}}_{{\tilde{P}}^{\left(C_{n},1\right)}}\left(D_{0}|U^{n}=u^{n},V^{n}=v^{n}\right)\\ \; & ={\bar{P}}_{{\tilde{P}}^{\left(C_{n},1\right)}}\left(E_{\mathrm{NE}}|U^{n}=u^{n},V^{n}=v^{n}\right)\;{\bar{P}}_{{\tilde{P}}^{\left(C_{n},1\right)}}\left(D_{0}|U^{n}=u^{n},V^{n}=v^{n},E_{\mathrm{NE}}\right)\\ \; & \leq {\bar{P}}_{{\tilde{P}}^{\left(C_{n},1\right)}}\left(D_{0}|U^{n}=u^{n},V^{n}=v^{n},E_{\mathrm{NE}}\right)\\ \; & =\sum_{j,\tilde{m}}{\bar{P}}_{{\tilde{P}}^{\left(C_{n},1\right)}}\left(J=j,f_{B}\left(J\right)=\tilde{m}|U^{n}=u^{n},V^{n}=v^{n},E_{\mathrm{NE}}\right)\\ \; & \;\;{\bar{P}}_{{\tilde{P}}^{\left(C_{n},1\right)}}\left(D_{0}|\;U^{n}=u^{n},V^{n}=v^{n},J=j,f_{B}\left(J\right)=\tilde{m},E_{\mathrm{NE}}\right) \end{array}$$

(A41) $$\begin{array}{cc} \; & ={\bar{P}}_{{\tilde{P}}^{\left(C_{n},1\right)}}\left(D_{0}|\;U^{n}=u^{n},V^{n}=v^{n},J=1,f_{B}\left(J\right)=1,E_{\mathrm{NE}}\right)\\ \; & =\sum_{\begin{array}{c} w^{n}\in W^{n} \end{array}}{\bar{P}}_{{\tilde{P}}^{\left(C_{n},1\right)}}\left(W^{n}\left(1\right)=w^{n}|U^{n}=u^{n},V^{n}=v^{n},J=1,f_{B}\left(J\right)=1,\;E_{\mathrm{NE}}\right) \end{array}$$

(A42) $$\begin{array}{ccc} \; & \;\;\;\;{\bar{P}}_{{\tilde{P}}^{\left(C_{n},1\right)}}\left(D_{0}|U^{n}=u^{n},V^{n}=v^{n},J=1,f_{B}\left(J\right)=1,W^{n}\left(1\right)=w^{n},\;E_{\mathrm{NE}}\right). & \; \end{array}$$

where (A41) follows since the term in (A40) is independent of the indices $\left(j,\tilde{m}\right)$ due to the symmetry of the codebook generation, encoding and decoding procedure. The first term in (A42) can be upper bounded as

(A43) $$\begin{array}{ccc} \; & {\bar{P}}_{{\tilde{P}}^{\left(C_{n},1\right)}}\left(W^{n}\left(1\right)=w^{n}|U^{n}=u^{n},V^{n}=v^{n},J=1,f_{B}\left(J\right)=1,\;E_{\mathrm{NE}}\right)\\ \; & \leq \frac{1}{|T_{P_{\tilde{W}|\tilde{U}}}|}\leq e^{-n(H\left(\tilde{W}|\tilde{U}\right)-\frac{1}{n}|U||W|\log\left(n+1\right))}. & \; \end{array}$$

To obtain (A43), we used the fact that $P_{E_{u}}^{\left(B_{n}\right)}\left(1|u^{n}\right)$ in (A20) is invariant to the joint type $P_{\tilde{U}\tilde{W}}$ of $\left(U^{n},W^{n}\left(1\right)\right)=\left(u^{n},w^{n}\right)$ (keeping all the other codewords fixed). This in turn implies that given $E_{\mathrm{NE}}$, each sequence in the conditional type class $T_{P_{\tilde{W}|\tilde{U}}}\left(u^{n}\right)$ is equally likely (in the randomness induced by $B_{n}$ and stochastic encoding in (A20)) and its probability is upper bounded by $\frac{1}{\left|T_{P_{\tilde{W}|\tilde{U}}}\right|}$. Defining the events

(A44) $$\begin{array}{cc} \; & E_{\mathrm{BE}}:=\left\{\exists \;l\in \left[M_{n}^{\prime }\right],\;l\neq J,\;f_{B}\left(l\right)=M,\;W^{n}\left(l\right))\in T_{{\left[P_{W}\right]}_{\hat{\delta }}}^{n},\left(V^{n},W^{n}\left(l\right)\right)\in T_{{\left[P_{VW}\right]}_{\tilde{\delta }}}^{n}\right\}, \end{array}$$

(A45) $$\begin{array}{cc} \; & F:=\{U^{n}=u^{n},V^{n}=v^{n},J=1,f_{B}\left(J\right)=1,W^{n}\left(1\right)=w^{n},\;E_{\mathrm{NE}}\}, \end{array}$$

(A46) $$\begin{array}{cc} \; & F_{1}:=\left\{U^{n}=u^{n},V^{n}=v^{n},J=1,f_{B}\left(J\right)=1,W^{n}\left(1\right)=w^{n},\;E_{\mathrm{NE}},\;E_{\mathrm{BE}}^{c}\right\}, \end{array}$$

(A47) $$\begin{array}{ccc} \mathrm{and}\; & F_{2}:=\left\{U^{n}=u^{n},V^{n}=v^{n},J=1,f_{B}\left(J\right)=1,W^{n}\left(1\right)=w^{n},\;E_{\mathrm{NE}},\;E_{\mathrm{BE}}\right\}, & \; \end{array}$$

the last term in (A42) can be written as

(A48) $$\begin{array}{cc} \; & {\bar{P}}_{{\tilde{P}}^{\left(C_{n},1\right)}}\left(D_{0}|F\right)={\bar{P}}_{{\tilde{P}}^{\left(C_{n},1\right)}}\left(E_{\mathrm{BE}}^{c}|F\right)\;{\bar{P}}_{{\tilde{P}}^{\left(C_{n},1\right)}}\left(D_{0}|F_{1}\right)+{\bar{P}}_{{\tilde{P}}^{\left(C_{n},1\right)}}\left(E_{\mathrm{BE}}|F\right)\;{\bar{P}}_{{\tilde{P}}^{\left(C_{n},1\right)}}\left(D_{0}|F_{2}\right). \end{array}$$

The analysis of the terms in (A48) is essentially similar to that given in the proof of Theorem 2 in [13], except for a subtle difference that we mention next. To bound the *binning* error event $E_{\mathrm{BE}}$, we require an upper bound similar to

(A49) $$\begin{array}{c} {\bar{P}}_{{\tilde{P}}^{\left(C_{n},1\right)}}\left(W^{n}\left(l\right)={\tilde{w}}^{n}|F\right)\leq 2\;{\bar{P}}_{{\tilde{P}}^{\left(C_{n},1\right)}}\left(W^{n}\left(l\right)={\tilde{w}}^{n}\right),\;\forall \;{\tilde{w}}^{n}\in W^{n}, \end{array}$$

that is used in the proof of Theorem 2 in [13]. Please note that the stochastic encoding scheme considered here is different from the encoding scheme in [13]. In place (A49), we will show that for l≠1,

(A50) $$\begin{array}{cc} \; & {\bar{P}}_{{\tilde{P}}^{\left(C_{n},1\right)}}\left(W^{n}\left(l\right)={\tilde{w}}^{n}|\;F\right)\leq 3\;{\bar{P}}_{{\tilde{P}}^{\left(C_{n},1\right)}}\left(W^{n}\left(l\right)={\tilde{w}}^{n}\right), \end{array}$$

which suffices for the proof. Please note that

(A51) $$\begin{array}{cc} \; & {\bar{P}}_{{\tilde{P}}^{\left(C_{n},1\right)}}\left(W^{n}\left(l\right)={\tilde{w}}^{n}|F\right)\\ \; & ={\bar{P}}_{{\tilde{P}}^{\left(C_{n},1\right)}}\left(W^{n}\left(l\right)={\tilde{w}}^{n}|U^{n}=u^{n},V^{n}=v^{n}\right)\frac{{\bar{P}}_{{\tilde{P}}^{\left(C_{n},1\right)}}\left(W^{n}\left(1\right)=w^{n}|W^{n}\left(l\right)={\tilde{w}}^{n},U^{n}=u^{n},V^{n}=v^{n}\right)}{{\bar{P}}_{{\tilde{P}}^{\left(C_{n},1\right)}}\left(W^{n}\left(1\right)=w^{n}|U^{n}=u^{n},V^{n}=v^{n}\right)}\\ \; & \;\frac{{\bar{P}}_{{\tilde{P}}^{\left(C_{n},1\right)}}\left(J=1|W^{n}\left(1\right)=w^{n},W^{n}\left(l\right)={\tilde{w}}^{n},U^{n}=u^{n},V^{n}=v^{n}\right)}{{\bar{P}}_{{\tilde{P}}^{\left(C_{n},1\right)}}\left(J=1|W^{n}\left(1\right)=w^{n},U^{n}=u^{n},V^{n}=v^{n}\right)}\\ \; & \;\frac{{\bar{P}}_{{\tilde{P}}^{\left(C_{n},1\right)}}\left(f_{B}\left(J\right)=1|J=1,W^{n}\left(1\right)=w^{n},W^{n}\left(l\right)={\tilde{w}}^{n},U^{n}=u^{n},V^{n}=v^{n}\right)}{{\bar{P}}_{{\tilde{P}}^{\left(C_{n},1\right)}}\left(f_{B}\left(J\right)=1|J=1,W^{n}\left(1\right)=w^{n},U^{n}=u^{n},V^{n}=v^{n}\right)} \end{array}$$

(A52) $$\begin{array}{ccc} \; & \;\frac{{\bar{P}}_{{\tilde{P}}^{\left(C_{n},1\right)}}\left(E_{\mathrm{NE}}|f_{B}\left(J\right)=1,J=1,W^{n}\left(1\right)=w^{n},W^{n}\left(l\right)={\tilde{w}}^{n},U^{n}=u^{n},V^{n}=v^{n}\right)}{{\bar{P}}_{{\tilde{P}}^{\left(C_{n},1\right)}}\left(E_{\mathrm{NE}}|f_{B}\left(J\right)=1,J=1,W^{n}\left(1\right)=w^{n},U^{n}=u^{n},V^{n}=v^{n}\right)} & \; \end{array}$$

Since the codewords are generated independently of each other and the binning operation is done independent of the codebook generation, we have

(A53) $$\begin{array}{cc} \; & {\bar{P}}_{{\tilde{P}}^{\left(C_{n},1\right)}}\left(W^{n}\left(1\right)=w^{n}|W^{n}\left(l\right)={\tilde{w}}^{n},U^{n}=u^{n},V^{n}=v^{n}\right)={\bar{P}}_{{\tilde{P}}^{\left(C_{n},1\right)}}\left(W^{n}\left(1\right)=w^{n}|U^{n}=u^{n},V^{n}=v^{n}\right), \end{array}$$

and

(A54) $$\begin{array}{cc} \; & {\bar{P}}_{{\tilde{P}}^{\left(C_{n},1\right)}}\left(f_{B}\left(J\right)=1|J=1,W^{n}\left(1\right)=w^{n},W^{n}\left(l\right)={\tilde{w}}^{n},U^{n}=u^{n},V^{n}=v^{n}\right)\\ \; & ={\bar{P}}_{{\tilde{P}}^{\left(C_{n},1\right)}}\left(f_{B}\left(J\right)=1|J=1,W^{n}\left(1\right)=w^{n},U^{n}=u^{n},V^{n}=v^{n}\right). \end{array}$$

Also, note that

(A55) $$\begin{array}{cc} \; & {\bar{P}}_{{\tilde{P}}^{\left(C_{n},1\right)}}\left(E_{\mathrm{NE}}|f_{B}\left(J\right)=1,J=1,W^{n}\left(1\right)=w^{n},W^{n}\left(l\right)={\tilde{w}}^{n},U^{n}=u^{n},V^{n}=v^{n}\right)\\ \; & ={\bar{P}}_{{\tilde{P}}^{\left(C_{n},1\right)}}\left(E_{\mathrm{NE}}|f_{B}\left(J\right)=1,J=1,W^{n}\left(1\right)=w^{n},U^{n}=u^{n},V^{n}=v^{n}\right). \end{array}$$

Next, consider the term in (A51). Let

$$\begin{array}{cc} \; & F^{\prime }:=\left\{W^{n}\left(1\right)=w^{n},U^{n}=u^{n},V^{n}=v^{n}\right\},\\ \; & F^{\prime \prime }:=\left\{W^{n}\left(1\right)=w^{n},W^{n}\left(l\right)={\tilde{w}}^{n},U^{n}=u^{n},V^{n}=v^{n}\right\}. \end{array}$$

Then, the numerator and denominator of (A51) can be written as

(A56) $$\begin{array}{ccc} \; & {\bar{P}}_{{\tilde{P}}^{\left(C_{n},1\right)}}\left(J=1|F^{\prime \prime }\right)\\ \; & =E_{\mu_{n}}\left[\frac{\prod_{i=1}^{n}P_{U|W}\left(u_{i}|w_{i}\right)}{\prod_{i=1}^{n}P_{U|W}\left(u_{i}|w_{i}\right)+\prod_{i=1}^{n}P_{U|W}\left(u_{i}|{\tilde{w}}_{i}\right)+\sum_{j\neq 1,l}\prod_{i=1}^{n}P_{U|W}\left(u_{i}|W_{i}\left(j\right)\right)}\right]\\ \; & \leq E_{\mu_{n}}\left[\frac{\prod_{i=1}^{n}P_{U|W}\left(u_{i}|w_{i}\right)}{\prod_{i=1}^{n}P_{U|W}\left(u_{i}|w_{i}\right)+\sum_{j\neq 1,l}\prod_{i=1}^{n}P_{U|W}\left(u_{i}|W_{i}\left(j\right)\right)}\right], & \; \end{array}$$

and

(A57) $$\begin{array}{ccc} \; & {\bar{P}}_{{\tilde{P}}^{\left(C_{n},1\right)}}\left(J=1|F^{\prime }\right)=E_{\mu_{n}}\left[\frac{\prod_{i=1}^{n}P_{U|W}\left(u_{i}|w_{i}\right)}{\prod_{i=1}^{n}P_{U|W}\left(u_{i}|w_{i}\right)+\sum_{j\neq 1}\prod_{i=1}^{n}P_{U|W}\left(u_{i}|W_{i}\left(j\right)\right)}\right], & \; \end{array}$$

respectively. The R.H.S. of (A56) (resp. (A57)) denote the average probability that J=1 is chosen by $P_{E_{u}}^{\left(B_{n}\right)}$ given $W^{n}\left(1\right)=w^{n}$, $U^{n}=u^{n}$ and $M_{n}^{\prime }-2$ (resp. $M_{n}^{\prime }-1$) other independent codewords in $B_{n}$. Let

$$\begin{array}{c} E_{l}:=\left\{\prod_{i=1}^{n}P_{U|W}\left(u_{i}|W_{i}\left(l\right)\right)\geq \max\left(\left\{\prod_{i=1}^{n}P_{U|W}\left(u_{i}|W_{i}\left(j\right)\right),\;j\in \left\lceil M_{n}^{\prime }\right\rceil \setminus \left\{1\right\}\right\}\cup \left\{\prod_{i=1}^{n}P_{U|W}\left(u_{i}|w_{i}\right)\right\}\right)\right\}. \end{array}$$

Please note that

(A58) $$\begin{array}{ccc} \; & E_{\mu_{n}|E_{l}^{c}}\left[\frac{\prod_{i=1}^{n}P_{U|W}\left(u_{i}|w_{i}\right)}{\prod_{i=1}^{n}P_{U|W}\left(u_{i}|w_{i}\right)+\sum_{j\neq 1}\prod_{i=1}^{n}P_{U|W}\left(u_{i}|W_{i}\left(j\right)\right)}\right]\\ \; & \geq \frac{1}{2}E_{\mu_{n}|E_{l}^{c}}\left[\frac{\prod_{i=1}^{n}P_{U|W}\left(u_{i}|w_{i}\right)}{\prod_{i=1}^{n}P_{U|W}\left(u_{i}|w_{i}\right)+\sum_{j\neq 1,l}\prod_{i=1}^{n}P_{U|W}\left(u_{i}|W_{i}\left(j\right)\right)}\right]. & \; \end{array}$$

Hence, denoting by ${\bar{\mu }}_{n}$ the probability measure induced by $\mu_{n}$, we have

(A59) $$\begin{array}{cc} \; & \frac{{\bar{P}}_{{\tilde{P}}^{\left(C_{n},1\right)}}\left(J=1|F^{\prime \prime }\right)}{{\bar{P}}_{{\tilde{P}}^{\left(C_{n},1\right)}}\left(J=1|F^{\prime }\right)}\\ \; & \leq \frac{E_{\mu_{n}}\left[\frac{\prod_{i=1}^{n}P_{U|W}\left(u_{i}|w_{i}\right)}{\prod_{i=1}^{n}P_{U|W}\left(u_{i}|w_{i}\right)+\sum_{j\neq 1,l}\prod_{i=1}^{n}P_{U|W}\left(u_{i}|W_{i}\left(j\right)\right)}\right]}{E_{\mu_{n}}\left[\frac{\prod_{i=1}^{n}P_{U|W}\left(u_{i}|w_{i}\right)}{\prod_{i=1}^{n}P_{U|W}\left(u_{i}|w_{i}\right)+\sum_{j\neq 1}\prod_{i=1}^{n}P_{U|W}\left(u_{i}|W_{i}\left(j\right)\right)}\right]}\\ \; & \leq \frac{E_{\mu_{n}}\left[\frac{\prod_{i=1}^{n}P_{U|W}\left(u_{i}|w_{i}\right)}{\prod_{i=1}^{n}P_{U|W}\left(u_{i}|w_{i}\right)+\sum_{j\neq 1,l}\prod_{i=1}^{n}P_{U|W}\left(u_{i}|W_{i}\left(j\right)\right)}\right]}{{\bar{\mu }}_{n}\left(E_{l}^{c}\right)E_{\mu_{n}|E_{l}^{c}}\left[\frac{\prod_{i=1}^{n}P_{U|W}\left(u_{i}|w_{i}\right)}{\prod_{i=1}^{n}P_{U|W}\left(u_{i}|w_{i}\right)+\sum_{j\neq 1}\prod_{i=1}^{n}P_{U|W}\left(u_{i}|W_{i}\left(j\right)\right)}\right]}\\ \; & \leq \frac{E_{\mu_{n}}\left[\frac{\prod_{i=1}^{n}P_{U|W}\left(u_{i}|w_{i}\right)}{\prod_{i=1}^{n}P_{U|W}\left(u_{i}|w_{i}\right)+\sum_{j\neq 1,l}\prod_{i=1}^{n}P_{U|W}\left(u_{i}|W_{i}\left(j\right)\right)}\right]}{\frac{1}{2}{\bar{\mu }}_{n}\left(E_{l}^{c}\right)E_{\mu_{n}|E_{l}^{c}}\left[\frac{\prod_{i=1}^{n}P_{U|W}\left(u_{i}|w_{i}\right)}{\prod_{i=1}^{n}P_{U|W}\left(u_{i}|w_{i}\right)+\sum_{j\neq 1,l}\prod_{i=1}^{n}P_{U|W}\left(u_{i}|W_{i}\left(j\right)\right)}\right]} \end{array}$$

(A60) $$\begin{array}{cc} \; & =\frac{E_{\mu_{n}}\left[\frac{\prod_{i=1}^{n}P_{U|W}\left(u_{i}|w_{i}\right)}{\prod_{i=1}^{n}P_{U|W}\left(u_{i}|w_{i}\right)+\sum_{j\neq 1,l}\prod_{i=1}^{n}P_{U|W}\left(u_{i}|W_{i}\left(j\right)\right)}\right]}{\frac{1}{2}E_{\mu_{n}}\left[\frac{\prod_{i=1}^{n}P_{U|W}\left(u_{i}|w_{i}\right)}{\prod_{i=1}^{n}P_{U|W}\left(u_{i}|w_{i}\right)+\sum_{j\neq 1,l}\prod_{i=1}^{n}P_{U|W}\left(u_{i}|W_{i}\left(j\right)\right)}\right]-\frac{1}{2}{\bar{\mu }}_{n}\left(E_{l}\right)E_{\mu_{n}|E_{l}}\left[\frac{\prod_{i=1}^{n}P_{U|W}\left(u_{i}|w_{i}\right)}{\prod_{i=1}^{n}P_{U|W}\left(u_{i}|w_{i}\right)+\sum_{j\neq 1,l}\prod_{i=1}^{n}P_{U|W}\left(u_{i}|W_{i}\left(j\right)\right)}\right]} \end{array}$$

(A61) $$\begin{array}{cc} \; & \leq \frac{E_{\mu_{n}}\left[\frac{\prod_{i=1}^{n}P_{U|W}\left(u_{i}|w_{i}\right)}{\prod_{i=1}^{n}P_{U|W}\left(u_{i}|w_{i}\right)+\sum_{j\neq 1,l}\prod_{i=1}^{n}P_{U|W}\left(u_{i}|W_{i}\left(j\right)\right)}\right]}{\frac{1}{2}E_{\mu_{n}}\left[\frac{\prod_{i=1}^{n}P_{U|W}\left(u_{i}|w_{i}\right)}{\prod_{i=1}^{n}P_{U|W}\left(u_{i}|w_{i}\right)+\sum_{j\neq 1,l}\prod_{i=1}^{n}P_{U|W}\left(u_{i}|W_{i}\left(j\right)\right)}\right]-\frac{1}{2}{\bar{\mu }}_{n}\left(E_{l}\right)} \end{array}$$

(A62) $$\begin{array}{cc} \; & \leq \frac{E_{\mu_{n}}\left[\frac{\prod_{i=1}^{n}P_{U|W}\left(u_{i}|w_{i}\right)}{\prod_{i=1}^{n}P_{U|W}\left(u_{i}|w_{i}\right)+\sum_{j\neq 1,l}\prod_{i=1}^{n}P_{U|W}\left(u_{i}|W_{i}\left(j\right)\right)}\right]}{\frac{1}{2}E_{\mu_{n}}\left[\frac{\prod_{i=1}^{n}P_{U|W}\left(u_{i}|w_{i}\right)}{\prod_{i=1}^{n}P_{U|W}\left(u_{i}|w_{i}\right)+\sum_{j\neq 1,l}\prod_{i=1}^{n}P_{U|W}\left(u_{i}|W_{i}\left(j\right)\right)}\right]-e^{-e^{n(I_{P}\left(U;W\right)+\eta^{\prime })}}} \end{array}$$

(A63) $$\begin{array}{ccc} \; & \leq 2+o(1)\leq 3, & \; \end{array}$$

where (A59) is due to (A58); (A61) is since the term within $E_{\mu_{n}|E_{l}}\left[\cdot \right]$ in (A60) is upper bounded by one; (A62) is since ${\bar{\mu }}_{n}\left(E_{l}\right)\leq e^{-e^{n(I_{P}\left(U;W\right)+\eta^{\prime })}}$ for some $\eta^{\prime }>0$ which follows similar to ([68], Section 3.6.3), and (A63) follows since the term within the expectation which is exponential in order dominates the double exponential term. From (A52)–(A55), (A63) and (A50) follows. The analysis of the other terms in (A48) is the same as in the SHA scheme in [7], and results in the error exponent (within an additive O(δ) term) claimed in the Theorem. We refer the reader to ([13], Theorem 2) for a detailed proof (In [13], the communication channel between the observer and the detector is a DMC. However, since the coding scheme used in the achievability part of Theorem 2 in [13] is a separation-based scheme, the error exponent when the channel is noiseless can be recovered by setting $E_{3}\left(\cdot \right)$ and $E_{4}\left(\cdot \right)$ in Theorem 2 to *∞*). By the random coding argument followed by the standard expurgation technique [72] (see ([13], Proof of Theorem 2)), there exists a deterministic codebook and binning function pair $C_{n}=\left(B_{n},f_{B}\right)$ such that the type I and type II error probabilities are within a constant multiplicative factor of their average values over the random ensemble $C_{n}$, and

(A64) $$\begin{array}{cc} \; & S_{i}-\left(w_{i}\left(J\right),V_{i}\right)-\left(M,w^{n}\left(J\right),V^{n},S^{i-1}\right),\;i\in \left[n\right], \end{array}$$

(A65) $$\begin{array}{cc} \; & ∥{\tilde{P}}^{\left(C_{n},0\right)}-\Psi^{\left(C_{n},0\right)}∥\leq e^{-\gamma_{4}n}, \end{array}$$

(A66) $$\begin{array}{cc} \; & ∥{\tilde{P}}^{\left(C_{n},1\right)}-\Psi^{\left(C_{n},1\right)}∥\leq e^{-\gamma_{4}n},\;\;ifQ_{U}=P_{U}, \end{array}$$

(A67) $$\begin{array}{cc} \mathrm{and}\; & \sum_{i=1}^{n}∥P_{W}-\Psi_{w_{i}\left(J\right)}^{\left(C_{n},l\right)}∥\leq e^{-\gamma_{5}n},\;l=0,1, \end{array}$$

where $\gamma_{4}$ and $\gamma_{5}$ are some positive numbers. Since the average type I error probability for our scheme tends to zero asymptotically, and the error exponent is unaffected by a constant multiplicative scaling of the type II error probability, this codebook achieves the same type I error probability and error exponent as the average over the random ensemble. Using this deterministic codebook for encoding and decoding, we first lower bound the equivocation and average distortion of $S^{n}$ at the detector as follows:

First consider the equivocation of $S^{n}$ under the null hypothesis.

$$\begin{array}{cc} H_{{\tilde{P}}^{\left(C_{n},0\right)}}\left(S^{n}|M,V^{n}\right) & \geq P_{{\tilde{P}}^{\left(C_{n},0\right)}}\left(M\neq 0\right)H\left(S^{n}|M\neq 0,V^{n}\right) \end{array}$$

(A68) $$\begin{array}{cc} \; & \geq \left(1-e^{-n\Omega (\delta )}\right)\;H_{{\tilde{P}}^{\left(C_{n},0\right)}}\left(S^{n}|M\neq 0,V^{n}\right) \end{array}$$

(A69) $$\begin{array}{cc} \; & \geq \left(1-e^{-n\Omega (\delta )}\right)\;H_{{\tilde{P}}^{\left(C_{n},0\right)}}\left(S^{n}|w^{n}\left(J\right),V^{n}\right) \end{array}$$

(A70) $$\begin{array}{cc} \; & =\left(1-e^{-n\Omega (\delta )}\right)\;H_{{\tilde{P}}^{\left(C_{n},0\right)}}\left(S^{n}|w^{n}\left(J\right),V^{n}\right) \end{array}$$

(A71) $$\begin{array}{cc} \; & \geq \left(1-e^{-n\Omega (\delta )}\right)\;H_{\Psi^{\left(C_{n},0\right)}}\left(S^{n}|w^{n}\left(J\right),V^{n}\right)-2e^{-\gamma_{4}n}\log\left(\frac{{\left|S\right|}^{n}{\left|V\right|}^{n}}{e^{-\gamma_{4}n}}\right) \end{array}$$

(A72) $$\begin{array}{cc} \; & =\sum_{i=1}^{n}H_{\Psi^{\left(C_{n},0\right)}}\left(S_{i}|w_{i}\left(J\right),V_{i}\right)-e^{-n\Omega (\delta )}\sum_{i=1}^{n}H_{\Psi^{\left(C_{n},0\right)}}\left(S_{i}|w_{i}\left(J\right),V_{i}\right)-o\left(1\right) \end{array}$$

(A73) $$\begin{array}{cc} \; & \geq \sum_{i=1}^{n}H_{\Psi^{\left(C_{n},0\right)}}\left(S_{i}|w_{i}\left(J\right),V_{i}\right)-ne^{-n\Omega (\delta )}H_{P}\left(S|V\right)-o\left(1\right) \end{array}$$

(A74) $$\begin{array}{cc} \; & =\left[\sum_{i=1}^{n}H_{\Psi^{\left(C_{n},0\right)}}\left(S_{i}|w_{i}\left(J\right),V_{i}\right)\right]-o\left(1\right) \end{array}$$

(A75) $$\begin{array}{ccc} \; & =nH_{P}\left(S|W,V\right)-o\left(1\right). & \; \end{array}$$

Here, (A68) follows from (A27); (A69) follows since *M* is a function of $w^{n}\left(J\right)$ for a deterministic codebook; (A71) follows from (A65) and Lemma 3; (A72) follows from (A24); and (A75) follows from (A67) and $\Psi_{S_{i}V_{i}|w_{i}}^{\left(0\right)}=P_{SV|W}^{\left(0\right)},\;i\in \left[n\right]$.

If $Q_{U}=P_{U}$, it follows similarly to above that

(A76) $$\begin{array}{cc} H_{{\tilde{P}}^{\left(C_{n},1\right)}}\left(S^{n}|M,V^{n}\right) & \geq \left(1-e^{-n\Omega (\delta )}\right)\;H_{\Psi^{\left(C_{n},1\right)}}\left(S^{n}|w^{n}\left(J\right),V^{n}\right)-2e^{-\gamma_{4}n}\log\left(\frac{{\left|S\right|}^{n}{\left|V\right|}^{n}}{e^{-\gamma_{4}n}}\right) \end{array}$$

(A77) $$\begin{array}{cc} \; & =\sum_{i=1}^{n}H_{\Psi^{\left(C_{n},1\right)}}\left(S_{i}|w_{i}\left(J\right),V_{i}\right)-e^{-n\Omega (\delta )}\sum_{i=1}^{n}H_{\Psi^{\left(C_{n},1\right)}}\left(S_{i}|w_{i}\left(J\right),V_{i}\right)-o\left(1\right) \end{array}$$

(A78) $$\begin{array}{cc} \; & \geq \sum_{i=1}^{n}H_{\Psi^{\left(C_{n},1\right)}}\left(S_{i}|w_{i}\left(J\right),V_{i}\right)-ne^{-n\Omega (\delta )}H_{Q}\left(S|V\right)-o\left(1\right) \end{array}$$

(A79) $$\begin{array}{cc} \; & =\left[\sum_{i=1}^{n}H_{\Psi^{\left(C_{n},1\right)}}\left(S_{i}|w_{i}\left(J\right),V_{i}\right)\right]-o\left(1\right) \end{array}$$

(A80) $$\begin{array}{ccc} \; & =nH_{Q}\left(S|W,V\right)-o\left(1\right). & \; \end{array}$$

Finally, consider the case H=1 and $Q_{U}\neq P_{U}$. We have for $\delta^{\prime }$ small enough that

(A81) $$\begin{array}{c} P_{{\tilde{P}}^{\left(C_{n},1\right)}}\left(M=0\right)=P_{{\tilde{P}}^{\left(C_{n},1\right)}}\left(U^{n}\notin T_{{\left[P_{U}\right]}_{\delta^{\prime }}}^{n}\right)\geq 1-e^{-n(D(P_{U}\left|\right|Q_{U})-O\left(\delta^{\prime }\right))}\overset{\left(n\right)}{\to }1. \end{array}$$

Hence, for $\delta^{\prime }$ small enough, we can write

$$\begin{array}{cc} \; & H_{{\tilde{P}}^{\left(C_{n},1\right)}}\left(S^{n}|M,V^{n}\right)\geq H_{{\tilde{P}}^{\left(C_{n},1\right)}}\left(S^{n}|M,V^{n},\Pi \left(U^{n},\delta^{\prime },P_{U}\right)\right) \end{array}$$

(A82) $$\begin{array}{cc} \; & \geq \left(1-e^{-n(D(P_{U}\left|\right|Q_{U})-O\left(\delta^{\prime }\right))}\right)\;H_{{\tilde{P}}^{\left(C_{n},1\right)}}\left(S^{n}|M,V^{n},\Pi \left(U^{n},\delta^{\prime },P_{U}\right)=1\right) \end{array}$$

(A83) $$\begin{array}{cc} \; & =\left(1-e^{-n(D(P_{U}\left|\right|Q_{U})-O\left(\delta^{\prime }\right))}\right)\;H_{{\tilde{P}}^{\left(C_{n},1\right)}}\left(S^{n}|V^{n},\Pi \left(U^{n},\delta^{\prime },P_{U}\right)=1\right) \end{array}$$

(A84) $$\begin{array}{cc} \; & \geq \left(1-e^{-n(D(P_{U}\left|\right|Q_{U})-O\left(\delta^{\prime }\right))}\right)\;\left(H_{{\tilde{P}}^{\left(C_{n},1\right)}}\left(S^{n}|V^{n}\right)-o\left(1\right)\right) \end{array}$$

(A85) $$\begin{array}{ccc} \; & =nH_{Q}\left(S|V\right)-ne^{-n(D(P_{U}\left|\right|Q_{U})-O\left(\delta^{\prime }\right))}H_{Q}\left(S|V\right)-o\left(1\right)=nH_{Q}\left(S|V\right)-o\left(1\right). & \; \end{array}$$

Here, (A82) follows from (A81); (A83) follows since $\Pi (U^{n},\delta^{\prime },P_{U})=1$ implies M=0; (A84) follows from Lemma 3 and (15). Thus, since δ>0 is arbitrary, we have shown that for ϵ∈(0,1), $\left(R,\kappa ,\Lambda_{0},\Lambda_{1}\right)\in R_{e}\left(\epsilon \right)$ if (18)–(21) holds.

On the other hand, average distortion of $S^{n}$ at the detector can be lower bounded under H=0 as follows:

$$\begin{array}{cc} \; & \min_{g_{i,n}^{\left(r\right)}}E\left[d\left(S^{n},{\hat{S}}^{n}\right)|H=0\right] \end{array}$$

(A86) $$\begin{array}{cc} \; & =\min_{{\left\{{\bar{\phi }}_{i,n}\left(m,v^{n},s^{i-1}\right)\right\}}_{i=1}^{n}}E_{{\tilde{P}}^{\left(C_{n},0\right)}}\left[\sum_{i=1}^{n}d\left(S_{i},{\bar{\phi }}_{i}\left(m,v^{n},s^{i-1}\right)\right)\right] \end{array}$$

(A87) $$\begin{array}{cc} \; & \geq \min_{{\left\{{\bar{\phi }}_{i}\left(m,v^{n},s^{i-1}\right)\right\}}_{i=1}^{n}}E_{\Psi^{\left(C_{n},0\right)}}\left[\sum_{i=1}^{n}d\left(S_{i},{\bar{\phi }}_{i}\left(m,v^{n},s^{i-1}\right)\right)\right]-ne^{-n\gamma_{4}}D_{m} \end{array}$$

(A88) $$\begin{array}{cc} \; & \geq \min_{{\left\{{\bar{\phi }}_{i}\left(\cdot ,\cdot \right)\right\}}_{i=1}^{n}}E_{\Psi^{\left(C_{n},0\right)}}\left[\sum_{i=1}^{n}d\left(S_{i},{\bar{\phi }}_{i}\left(w_{i}\left(J\right),V_{i}\right)\right)\right]-ne^{-n\gamma_{4}}D_{m} \end{array}$$

(A89) $$\begin{array}{cc} \; & \geq n\min_{\left\{\phi (\cdot ,\cdot )\right\}}E_{P}\left[d(S,\phi (W,V))\right]-n\left(e^{-n\gamma_{4}}+e^{-n\gamma_{5}}\right)D_{m} \end{array}$$

(A90) $$\begin{array}{ccc} \; & =n\min_{{\left\{\phi (\cdot ,\cdot )\right\}}_{i=1}^{n}}E_{P}\left[d(S,\phi (W,V))\right]-o\left(1\right). & \; \end{array}$$

Here, (A86) follows from Lemma 2; (A87) follows from ([43], Property 2(b)) due to (A65) and boundedness of distortion measure; (A88) follows from the Markov chain in (A64); (A89) follows from (A67) and the fact that $\Psi_{S_{i}V_{i}|w_{i}\left(J\right)}^{\left(0\right)}=P_{SV|W}^{\left(0\right)},\;i\in \left[n\right]$.

Next, consider the case H=1 and $Q_{U}=P_{U}$. Then, similarly to above, we can write

(A91) $$\begin{array}{cc} \; & \min_{g_{i,n}^{\left(r\right)}}E\left[d\left(S^{n},{\hat{S}}^{n}\right)|H=1\right]\\ \; & =\min_{{\left\{{\bar{\phi }}_{i}\left(m,v^{n},s^{i-1}\right)\right\}}_{i=1}^{n}}E_{{\tilde{P}}^{\left(C_{n},1\right)}}\left[\sum_{i=1}^{n}d\left(S_{i},\phi_{i}\left(M,V^{n},S^{i-1}\right)\right)\right]\\ \; & \geq \min_{{\left\{{\bar{\phi }}_{i}\left(m,v^{n},s^{i-1}\right)\right\}}_{i=1}^{n}}E_{\Psi^{\left(C_{n},1\right)}}\left[\sum_{i=1}^{n}d\left(S_{i},\phi_{i}\left(M,V^{n},S^{i-1}\right)\right)\right]-ne^{-n\gamma_{4}}D_{m} \end{array}$$

(A92) $$\begin{array}{cc} \; & \geq \min_{{\left\{\phi_{i}\left(\cdot ,\cdot \right)\right\}}_{i=1}^{n}}E_{\Psi^{\left(C_{n},1\right)}}\left[\sum_{i=1}^{n}d\left(S_{i},\phi_{i}\left(w_{i},V_{i}\right)\right)\right]-ne^{-n\gamma_{4}}D_{m} \end{array}$$

(A93) $$\begin{array}{cc} \; & \geq n\min_{{\left\{\phi (\cdot ,\cdot )\right\}}_{i=1}^{n}}E_{Q}\left[d(S,\phi (W,V))\right]-n\left(e^{-n\gamma_{4}}+e^{-n\gamma_{5}}\right)D_{m}. \end{array}$$

(A94) $$\begin{array}{ccc} \; & =n\min_{{\left\{\phi (\cdot ,\cdot )\right\}}_{i=1}^{n}}E_{Q}\left[d(S,\phi (W,V))\right]-o\left(1\right). & \; \end{array}$$

If H=1 and $Q_{U}\neq P_{U}$, we have

(A95) $$\begin{array}{cc} \; & \min_{g_{i,n}^{\left(r\right)}}E\left[d\left(S^{n},{\hat{S}}^{n}\right)|H=1\right]\\ \; & \geq P_{{\tilde{P}}^{\left(C_{n},1\right)}}\left(M=0|H=1\right)\min_{{\left\{{\bar{\phi }}_{i}\left(m,v^{n},s^{i-1}\right)\right\}}_{i=1}^{n}}\sum_{i=1}^{n}E_{{\tilde{P}}^{\left(C_{n},1\right)}}\left[d\left(S_{i},\phi_{i}\left(0,V^{n},S^{i-1}\right)\right)\right]\\ \; & \geq P_{{\tilde{P}}^{\left(C_{n},1\right)}}\left(M=0|H=1\right)\left[\min_{{\left\{\phi_{i}^{\prime }\left(v\right)\right\}}_{i=1}^{n}}E_{Q}\left[\sum_{i=1}^{n}d\left(S_{i},\phi_{i}^{\prime }\left(V_{i}\right)\right)\right]-D_{m}o\left(1\right)\right] \end{array}$$

(A96) $$\begin{array}{ccc} \; & =n\min_{\left\{\phi^{\prime }\left(\cdot \right)\right\}}E_{Q}\left[d(S,\phi^{\prime }\left(V\right))\right]-o\left(1\right). & \; \end{array}$$

Here, (A96) follows from (15) in Lemma 4 and (A96) follows from (A81). Thus, since δ>0 is arbitrary, we have shown that $\left(R,\kappa ,\Delta_{0},\Delta_{1}\right)\in R_{d}\left(\epsilon \right)$, ϵ∈(0,1), provided that (18), (19), (24) and (25) are satisfied. This completes the proof of the theorem.

## Appendix E. Proof of Lemma 5

Consider the |U|+2 functions of $P_{U|W}$,

(A97) $$\begin{array}{cc} \; & P_{U}\left(u_{i}\right)=\sum_{w\in W}P_{W}\left(w\right)P_{U|W}\left(u_{i}|w\right),i=1,2,\ldots ,\left|U\right|-1, \end{array}$$

(A98) $$\begin{array}{cc} \; & H_{P}\left(U|W,Z\right)=\sum_{w}P_{W}\left(w\right)g_{1}\left(P_{U|W},w\right), \end{array}$$

(A99) $$\begin{array}{cc} \; & H_{P}\left(Y|W,Z\right)=\sum_{w}P_{W}\left(w\right)g_{2}\left(P_{U|W},w\right), \end{array}$$

(A100) $$\begin{array}{cc} \; & H_{P}\left(S|W,Y,Z\right)=\sum_{w}P_{W}\left(w\right)g_{3}\left(P_{U|W},w\right), \end{array}$$

where

$$\begin{array}{cc} g_{1}\left(P_{U|W},w\right) & =-\sum_{u,z}P_{U|W}\left(u|w\right)P_{Z|U}\left(z|u\right)\log\left(\frac{P_{U|W}\left(u|w\right)P_{Z|U}\left(z|u\right)}{\sum_{u}P_{U|W}\left(u|w\right)P_{Z|U}\left(z|u\right)}\right),\\ g_{2}\left(P_{U|W},w\right) & =-\sum_{y,z,u}P_{U|W}\left(u|w\right)P_{YZ|U}\left(y,z|u\right)\log\left(\frac{\sum_{u}P_{U|W}\left(u|w\right)P_{YZ|U}\left(y,z|u\right)}{\sum_{u}P_{U|W}\left(u|w\right)P_{Z|U}\left(z|u\right)}\right),\\ g_{3}\left(P_{U|W},w\right) & =-\sum_{s,y,z,u}P_{U|W}\left(u|w\right)P_{SYZ|U}\left(s,y,z|u\right)\log\left(\frac{\sum_{u}P_{U|W}\left(u|w\right)P_{SYZ|U}\left(s,y,z|u\right)}{\sum_{u}P_{U|W}\left(u|w\right)P_{YZ|U}\left(y,z|u\right)}\right). \end{array}$$

Thus, by the Fenchel–Eggleston–Carathéodory’s theorem [68], it is sufficient to have at most |U|−1 points in the support of *W* to preserve $P_{U}$ and three more to preserve $H_{P}\left(U|W,Z\right)$, $H_{P}\left(Y|W,Z\right)$ and $H_{P}\left(S|W,Z,Y\right)$. Noting that $H_{P}\left(Y|Z\right)$ and $H_{P}\left(U|Z\right)$ are automatically preserved since $P_{U}$ is preserved (and (Y,Z,S)−U−W holds), |W|=|U|+2 points are sufficient to preserve the R.H.S. of Equations (28)–(30). This completes the proof for the case of $R_{e}$. Similarly, considering the |U|+1 functions of $P_{W|U}$ given in (A97)–(A99) and

$$\begin{array}{cc} E_{P}\left[d\left(S,\phi (W,Y,Z)\right)\right] & =\sum_{w}P_{W}\left(w\right)g_{4}\left(w,P_{W|U}\right), \end{array}$$

where

$$\begin{array}{cc} g_{4}\left(w,P_{W|U}\right) & =\sum_{s,u,y,z}P_{U|W}\left(u|w\right)P_{YZS|U}\left(y,z,s|u\right)\;d\left(s,\phi \left(w,y,z\right)\right), \end{array}$$

similar result holds also for the case of $R_{d}$.
